# Structure-guided optimisation of fenofibrate-derived oxidative phosphorylation inhibitors to modify tumour hypoxia

**DOI:** 10.1039/d5md00742a

**Published:** 2026-04-28

**Authors:** James P. Holt-Martyn, Nicole Machado, James T. T. Coates, Rathi Puliyadi, Thomas Ashton, Elysia Traynor, Samina Aslam, Thomas K. Wise, Gonzalo Rodriguez-Berriguete, Christopher J. Schofield, Geoff S. Higgins

**Affiliations:** a Department of Oncology, University of Oxford Oxford OX3 7DQ UK gonzalo.rodriguez@oncology.ox.ac.uk geoffrey.higgins@oncology.ox.ac.uk; b Chemistry Research Laboratory, Department of Chemistry and the Ineos Institute for Antimicrobial Research, University of Oxford Oxford OX1 3TA UK christopher.schofield@chem.ox.ac.uk

## Abstract

Solid tumours frequently manifest regions of abnormally low levels of oxygen (hypoxia), which negatively impacts cancer treatment outcomes. This is particularly detrimental to radiotherapy which requires oxygen to exert maximal therapeutic effects. Tumour hypoxia can be abolished by reducing oxygen consumption rates (OCR) through inhibition of oxidative phosphorylation (OXPHOS), though to date no hypoxia modifying OXPHOS inhibitors have successfully translated into routine clinical practise. Here, we demonstrate that the well-tolerated, pro-drug fenofibrate, which has moderate OXPHOS inhibitory activity, can serve as a scaffold for OXPHOS inhibitor development. Structural modification of the four different regions of fenofibrate, that is its isopropyl-, dimethyl-, chloro-, and ketone-groups, improves potency for OCR inhibition whilst eliminating ester hydrolysis. The derivatives improve hypoxia alleviation in 3D spheroid models, without inducing cytotoxicity. Substrate-dependent oxygen consumption assays support complex I-specific inhibition as the mechanism of action. Structure activity relationship studies led to development of a lead compound (IOX7), which demonstrates improved potency for OXPHOS inhibition, a superior solubility profile, and lack of *in vitro* cytotoxicity at effective doses compared to fenofibrate. IOX7 has the potential for development as a clinically useful hypoxia-modifying OXPHOS inhibitor.

## Abstract 2

Hypoxia, or low levels of oxygen, is a common feature of solid tumours that makes them resistant to cancer therapies. Electron transport chain inhibitors are a promising strategy for O_2_ redistribution to target tumour hypoxia during radiotherapy.

## Graphical abstract

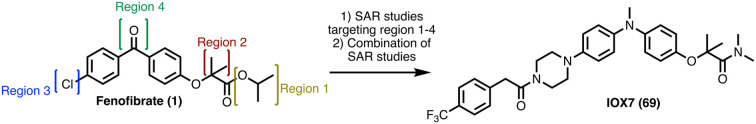

## Introduction

Radiotherapy (RT) is an essential pillar of cancer treatment, with the potential to improve curative rates in a quarter of cancer patients globally, and is predominantly used to treat solid tumours.^1^ However, the abnormal and chaotic vasculature and growth of solid tumours creates an imbalance between oxygen supply and demand, resulting in regions of oxygen deficiency called hypoxia (typically <2% O_2_).^2^ Hypoxia is a negative prognostic factor for radiation therapy as well as for other cancer treatments.^2–4^ Oxygen is required for the major cytotoxic effects of radiation, involving production of strand breaks in DNA, by chemically ‘fixing’ radiation-induced radicals to produce peroxides which are difficult for the cell to repair.^3^ In a hypoxic environment, where less oxygen is available, chemical fixation of DNA strand breaks occurs less frequently and up to three times the radiation dose is required to exert the same therapeutic effect as observed in a normoxic environment.^2,3,5^

Most approaches to alleviate tumour hypoxia have been based on directly targeting hypoxic cells, with hypoxic cytotoxins, or increasing the delivery of oxygen through hyperbaric oxygen therapy, carbogen treatment, or vascular remodelling.^3^ However, such therapies have major challenges, including hazardous delivery,^6,7^ poor perfusion to the target due to abnormal vasculature,^8^ intolerable toxicities,^9,10^ and limited efficacy confined to small patient populations, showing modest benefit in head and neck but not in brain, lung, uterine cervix, and other cancer patients.^11^ Despite more than five decades of research on targeting tumour hypoxia, as yet there has been no major translation into improved clinical practise.^12^

In the 1990s an alternative strategy to target tumour hypoxia was proposed - this focuses on oxygen redistribution rather than increases in oxygen delivery or targeting of hypoxic cells.^13^ It was proposed that modest reductions in cellular oxygen consumption, modelled to be ∼30%, should increase local oxygen availability in tumours to alleviate hypoxia and improve radiosensitivity.^14,15^

Oxygen consumption can be efficiently blunted by inhibiting oxidative phosphorylation (OXPHOS) and targeting the electron transport chain (ETC). Several ETC inhibitors have demonstrated tumour reoxygenation and metabolic radiosensitization in *in vitro* and *in vivo* cancer models.^16–19^ Papaverine and atovaquone are currently being assessed or have successfully completed clinical trials, respectively, in combination with radiation therapy (NCT05136846, NCT06834126, NCT03824327, NCT04648033 and NCT02628080). In fact, delivery of the complex III inhibitor atovaquone in non-small cell lung cancer (NSCLC) patients resulted in significant reduction of hypoxia in tumours in the absence of vascular normalization.^20^ Such an approach improves upon several of the aforementioned efforts as it does not require therapy delivery directly to hypoxic regions, unlike oxygen mimetics or hypoxic cytotoxins, and may overcome perfusion limitations. Moreover, the requirement of modest reductions in OXPHOS, delivered concurrently with radiation may enable improved safety profiles as delivery of prolonged, highly potent ETC inhibitors can be toxic.^21,22^ The balance between potency, safety and efficacy is delicate, as safe, but non-potent OXPHOS inhibitors such as metformin have failed to mediate therapeutic efficacy as chemo-radiosensitizers.^23,24^

To identify well-tolerated compounds that can also inhibit cellular oxygen consumption, we screened >1697 FDA-approved small molecules for their ability to reduce oxygen consumption rates (OCR).^17^ Fenofibrate (1), a widely used, hyperlipidaemia prodrug, was found to moderately reduce OCR in FaDu cells.^17^ Fenofibrate (1) is a known, weak inhibitor of complex I of the ETC, but its active metabolite fenofibric acid does not reduce OCR, an observation which may reflect differences in cell penetration from the carboxylic acid.^25–27^ Here, we show that fenofibrate (1) is an excellent starting scaffold for the development of well-tolerated complex I inhibitors for hypoxia alleviation in solid tumours. We describe structure activity relationship (SAR) studies on multiple regions of fenofibrate (1) leading to the development of IOX7, a potent non-ester complex I inhibitor that abolishes hypoxia without compromising cell viability.

## Results and discussion

### Fenofibrate (1) is an excellent scaffold for complex I inhibitor-development

**Fig. 1:**
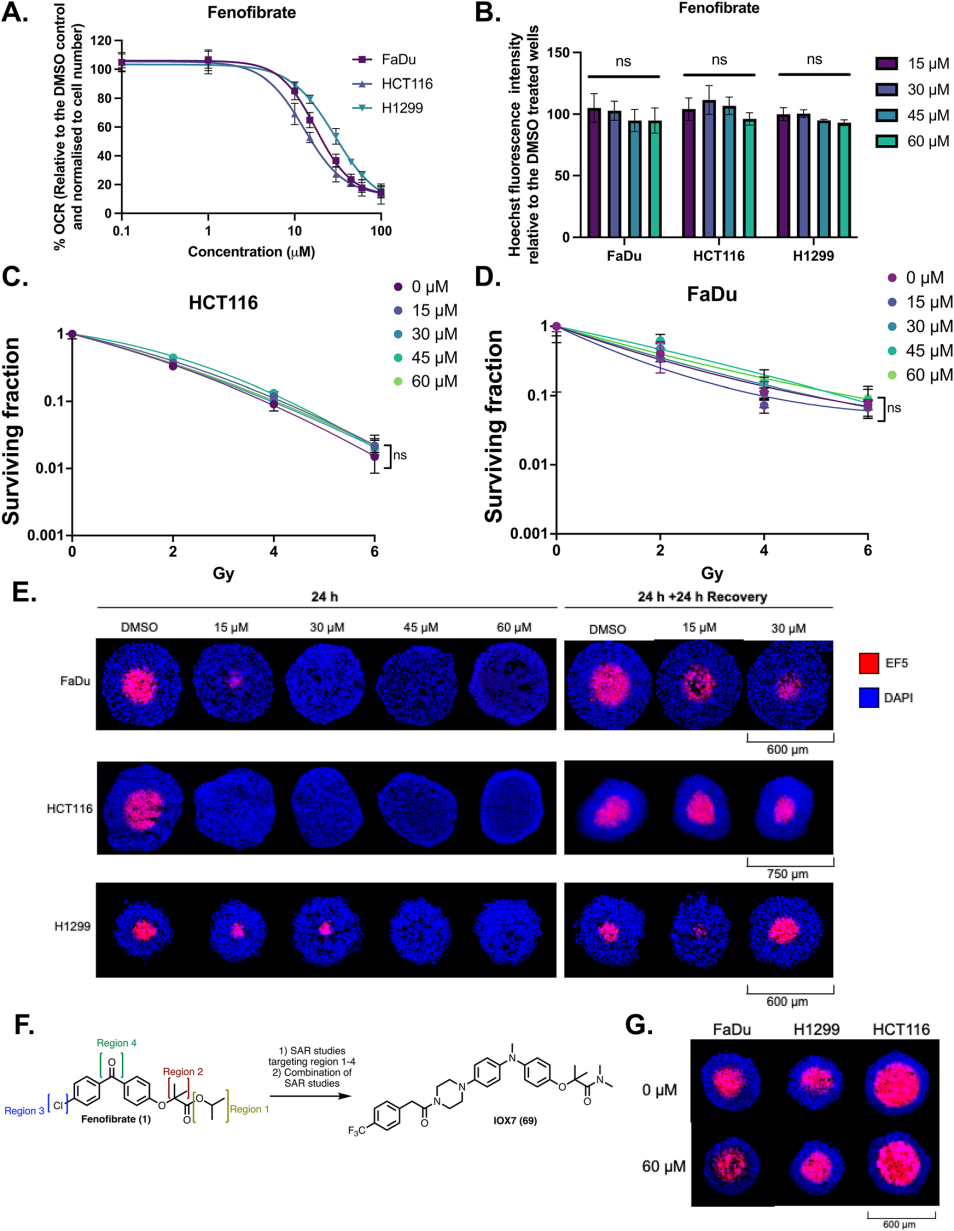
Fenofibrate (1) modifies metabolism in hypoxia *in vitro* and is a suitable scaffold for developing an OXPHOS inhibitor. (A) The oxygen consumption rates (OCRs) of FaDu, HCT116 and H1299 cells were measured 2.5 hours after port injections of fenofibrate in an XF Seahorse analyser at the indicated concentrations. The percent mean OCR is normalized to the number of cells and is shown relative to the DMSO control. IC_50_ curves are plotted as a non-linear regression. (B) Cell count plotted as relative Hoechst fluorescence intensity of cells fixed immediately after assay in (A). Error bars are standard deviation (SD, *n* = 3). One-way ANOVA with Bonferroni *post hoc* analysis was performed. (C) Surviving fractions of clonogenic assays of HCT116 treated with fenofibrate and radiation. Two-way ANOVA with Bonferroni *post hoc* analysis was performed comparing each treatment to an untreated control. (D) Same as (C) with FaDu cells. (E) Representative images of FaDu, HCT116, and H1299 spheroids treated with DMSO or fenofibrate after a 24 hour treatment, or one followed by recovery for an additional 24 hours in drug-free medium (as indicated). Hypoxia was assessed by staining central spheroid sections with EF5 (red), with DAPI as a nuclear counterstain (blue). (F) Schematic outline of the medicinal chemistry strategy – fenofibrate was divided into four regions for SAR assessment. (G) Representative images of FaDu, HCT116 and H1299 spheroids treated with DMSO or 60 μM fenofibric acid. Hypoxia was assessed by staining central spheroid sections for EF5 (red), with DAPI as a nuclear counterstain (blue). Scales are as indicated. All plotted data corresponds to an average ± SD from three independent experiments, where n.s. is not significant, ****P < 0.0001, ***P < 0.001, **P < 0.01, and *P < 0.05.

To investigate the feasibility of fenofibrate (1) as a starting point for developing a well-tolerated OXPHOS inhibitor, we carried out dose–response studies using FaDu, HCT116, and H1299 cells. These cell lines were chosen as they form highly hypoxic spheroids, which we considered a useful 3D model for subsequent experiments to assess the activity of hypoxia modifiers.^17^ Within 2.5 h of exposure, fenofibrate (1) reduced OCR in all the tested cell lines, with IC_50_ values: FaDu cells 17.90 μM (15.45–20.52, 95% CI), HCT116 cells 12.86 μM (11.14–15.33, 95% CI), H1299 cells 29.09 μM (25.27–35.01, 95% CI) (Fig. 1A). IC_50_ values fall within the plasma concentrations achieved in clinical practise for treating hyperlipidaemia (15–60 μM). Fenofibrate (1) significantly reduced OCR (Fig. 1A), with minimal impact on cell viability (Fig. 1B), demonstrating it to be a good starting point for developing an effective and safe hypoxic modifier.^28^ The sensitivity of tumours to radiation can be reduced by levels of hypoxia, but also by cell intrinsic properties such as defects in DNA repair. To further investigate whether fenofibrate (1) is a useful scaffold for developing a radiosensitizer that modifies hypoxia specifically, colony formation assays were used to rule out any cell intrinsic radiosensitization properties of fenofibrate (1) under normoxic conditions. No significant differences were observed between survival curves treated with fenofibrate (1) compared to the control in FaDu and HCT116 spheroids (Fig. 1C and D). This observation demonstrates that fenofibrate (1) is not an intrinsic radiosensitizer at the concentrations for which it significantly reduces OCR.

### Fenofibrate (1) affects spheroid hypoxia

Reduction of the OCR in 3D multicellular spheroids can lead to a decrease in hypoxia. Fenofibrate (1) completely abolishes hypoxia at 30 μM in FaDu and HCT116 spheroids and at 45 μM in H1299 spheroids (Fig. 1E, S1A and S1B). Following a 24-hour wash-out, restoration of hypoxia in spheroids confirmed hypoxia modification as a fenofibrate (1) dependent effect (Fig. 1E). None of the treatments caused significant decreases in the diameter of FaDu, HCT116, and H1299 spheroids (Fig. S1C and S1D). These results indicate that the observed decrease in the OCR induced by fenofibrate (1) corresponds to a decrease in hypoxia in a 3D *in vitro* cancer model, without influencing cytotoxicity.

### Structure activity relationship studies on different regions of fenofibrate (1)

To improve esterase stability and OCR potency, SAR studies were conducted in two stages. Initially, we investigated the effects on OCR inhibition of modifications to the four different regions of fenofibrate (1): 1, the isopropyl ester; 2, the dimethyl group; 3, the chloro group; and 4, the ketone group (Fig. 1F). We then combined the improved groups discovered in the initial SAR, aiming to produce a potent OCR inhibitor with a safety profile similar to that of fenofibrate (1).

With respect to region 1, the fenofibrate (1) metabolite fenofibric acid (2) had a weak effect on OCR compared to fenofibrate (1) (81% and 25% at 100 μM, respectively) and did not alleviate hypoxia in 3D multicellular spheroids at 60 μM (Fig. 1G and Table 1). Replacing the isopropyl ester of fenofibrate (1) with aliphatic esters such as ethyl ester (3), *tert*-butyl (4) or cyclohexyl ester (5) resulted in a similar level of OCR reduction to fenofibrate (1) (Table 1). Aromatic esters such as the phenyl (6) or benzyl (7) derivatives did not reduce OCR at any tested concentration. The methyl ketone (8) reduced OCR to a similar degree as fenofibrate (1) (88% at 10 μM) demonstrating an ester is not essential for potent activity (Table 1).

**Table 1:** 1–16 investigating the role of the isopropyl ester of fenofibrate (1) (region 1)

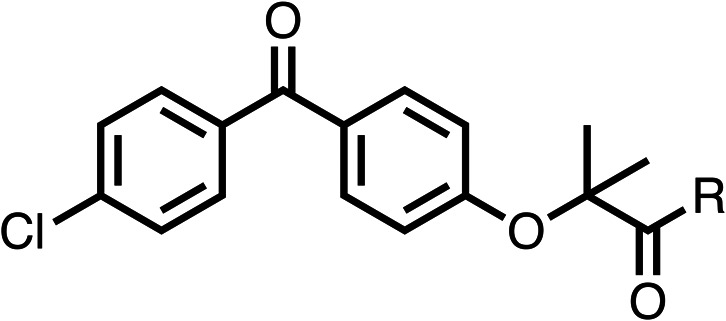 % OCR normalised to the DMSO controls															
Entry	R	100 μm	10 μm	Entry	R	100 μm	10 μm	Entry	R	100 μm	10 μm	Entry	R	100 μm	10 μm
1	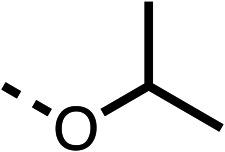	25 ± 1	94 ± 1	2	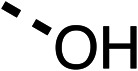	81 ± 1	>100	3	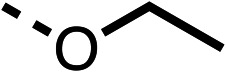	33 ± 3	>100	4	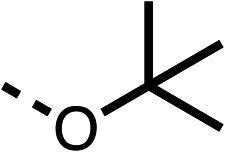	12 ± 5	81 ± 18
5	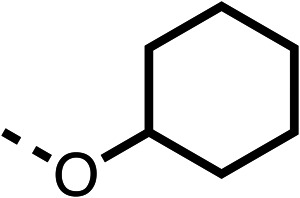	16 ± 5	>100	6	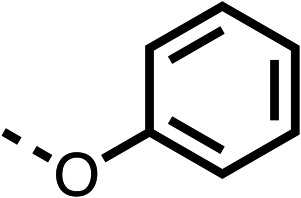	>100	>100	7	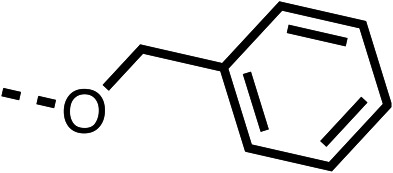	>100	>100	8	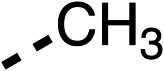	23 ± 4	88 ± 15
9	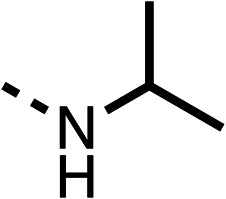	75	95	10	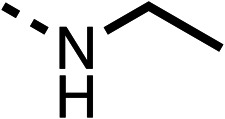	33 ± 18	99 ± 8	11	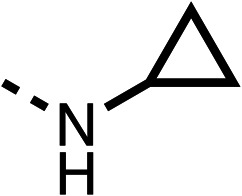	33	98	12	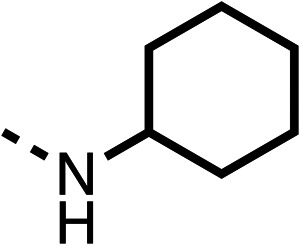	6 ± 3	22 ± 10
13	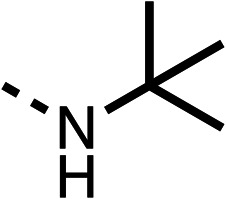	85	90	14	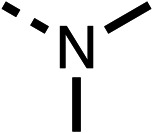	24 ± 2^a^	59 ± 2	15	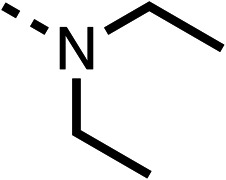	56 ± 2^a^	93 ± 2	16	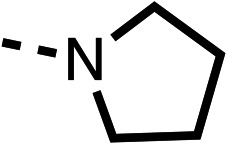	80	85

a Tested at 30 μM.

The amide (9) manifested weaker reductions in OCR (75% at 100 μM) compared to fenofibrate (1) (25% at 100 μM). The ethyl amide (10), however, resulted in the same level of OCR inhibition compared to the analogous ester (3) indicating the ester group can be replaced with an amide (33% and 33%, respectively). As the introduction of aromatic groups in the esters resulted in weaker activity (6, 7), we focused on amides with aliphatic groups. The cyclopropyl amide (11) had a similar level of OCR inhibition compared to 3 (33% and 33%, respectively, at 100 μM). The cyclohexyl amide (12) better inhibited OCRs at both 100 μM and 10 μM (6% and 22%, respectively, Table 1), though the *tert*-butyl amide derivative (13) was a relatively weak inhibitor of OCR (85% at 100 μM, Table 1). The dimethylamide derivative (14) inhibited OCR at 30 μM and 10 μM (24% and 59%, respectively, Table 1); however, increasing the size of the tertiary amide group of 14 to diethyl (15) or pyrrolidine (16) groups led to a loss of OCR inhibition at 10 μM (Table 1). In summary, the SAR studies on region 1, showed that likely metabolically more stable derivatives than fenofibrate (1), such as the cyclohexyl (12) and the dimethylamide (14) amides, manifest improved OCR inhibition activity compared to fenofibrate (1).

SAR studies on region 2, *i.e.* the dimethyl group of fenofibrate (1) (17–20, Table 2), revealed removal of both (17) or a single methyl group (18) results in a loss of OCR inhibition (88% and 77% at 100 μM, respectively) compared to the dimethyl containing fenofibrate (1) and 3 (25% and 33% at 100 μM, respectively). Replacement of the single methyl group of (18) with larger ethyl (19) or benzyl (20) groups at 100 μM resulted in similar OCR inhibition (52% and 27% at 100 μM, respectively, Table 2) compared to fenofibrate (1) and 3 (Table 1).

**Table 2:** 17–20 investigating the role of the dimethyl group of fenofibrate (1) (region 2)

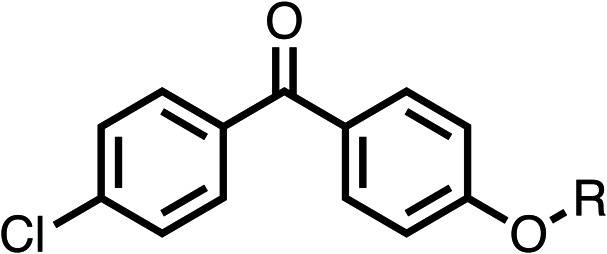 % OCR normalised to the DMSO controls															
Entry	R	100 μm	10 μm	Entry	R	100 μm	10 μm	Entry	R	100 μm	10 μm	Entry	R	100 μm	10 μm
17	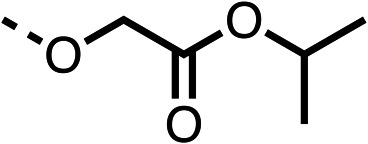	88 ± 1	98 ± 1	18	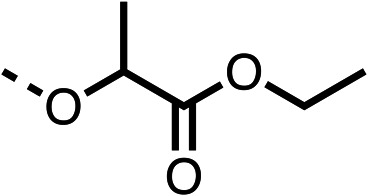	77 ± 26	>100	19	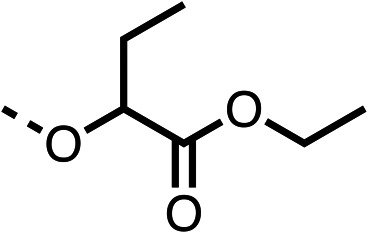	52 ± 28	84 ± 12	20	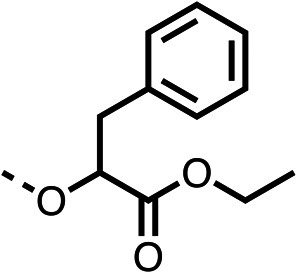	27 ± 4	89 ± 14

Due to the hydrophobic nature of fenofibrate (1), we replaced the chloride of region 3 with amino groups aiming to improve solubility (22–29). Removal of the chloro group (21) resulted in similar OCR inhibition (17% at 100 μM, Table 3) to fenofibrate (1), indicating C4 of the phenyl ring may be a good position for modification. The dimethylamine (22) and ethyl ester glycine (23) derivatives both potently inhibited OCR at 100 μM (5% and 11% respectively). However, 22 and 23 were substantially weaker OCR inhibitors at 10 μM (86% and 100% respectively, Table 3), albeit manifested similar OCR inhibition to fenofibrate (1). The benzyl amine (24) potently inhibited OCR at 100 μM (12%) but possessed moderate activity at 10 μM (54%, Table 3). The morpholine (25), 1-methyl-piperazine (26) and 1-*N*-piperazine (29) derivatives resulted in a weaker OCR inhibitory effect compared to fenofibrate (1) at 100 μM. However, *N*-acetyl-piperazine (27) and benzyoxycarbonyl piperazine (28) derivatives produced improved OCR inhibition at 10 μM (15% and 19% respectively, Table 3).

**Table 3:** 21–29 investigating the role of the chloride group of fenofibrate (1) (region 3)

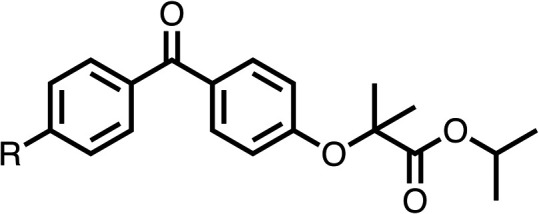 % OCR normalised to the DMSO controls											
Entry	R	100 μm	10 μm	Entry	R	100 μm	10 μm	Entry	R	100 μm	10 μm
21	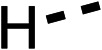	17 ± 9	85 ± 13	22	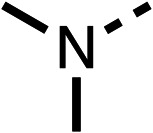	5 ± 2	86 ± 6	23	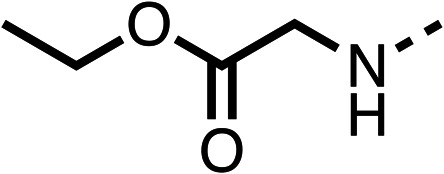	11 ± 6	100 ± 11
24	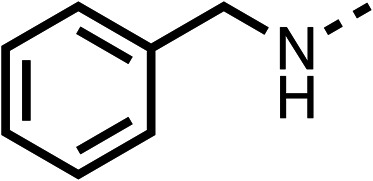	12 ± 3	54 ± 6	25	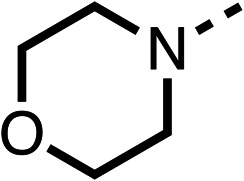	58 ± 7	88 ± 9	26	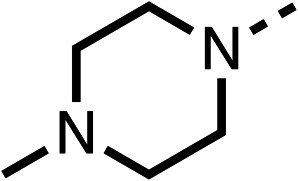	>100	>100
27	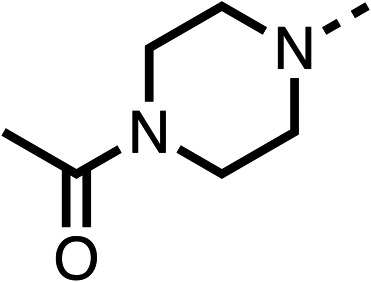	21 ± 3	15 ± 5	28	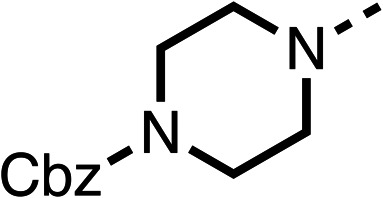	11 ± 5	19 ± 5	29	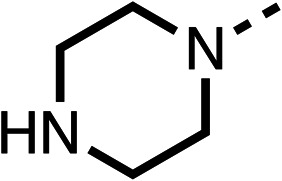	87 ± 3	70 ± 7

The potent effects on OCR inhibition of 27 and 28 led us to conduct further SAR studies focusing on the group linked to the terminal nitrogen of the piperazine ring such as heteroaryl rings and the functional group linker such as tertiary amines (30, 31), amides (32–35) and ureas (36 and 37, Table 4). The 3-methyl pyridine tertiary amine derivative (30) inhibited OCR (19% at 10 μM, Table 4) similarly to 28 (Table 3), whereas the 1-methyl imidazole derivative (31) was a weaker inhibitor of OCR (75% at 10 μM, Table 4). The 3-methyl pyridine amide derivative (32) moderately inhibited OCR (31% at 10 μM, Table 4). Interestingly the amide-pyrimidine derivative (33) was a weak inhibitor at 10 μM (73%, Table 4), consistent with the weak activity of 31. The 2-trifluorom ethyl phenyl (34) and 4-trifluoromethyl benzyl (35) amides both demonstrated potent OCR inhibition (6% and 6% at 10 μM, respectively, Table 4). The 3-pyridiyl urea derivative (36) was a moderate inhibitor of OCR (32% at 10 μM, Table 4). The glycine ethyl ester urea derivative (37) weakly inhibited OCR at 10 μM (88%, Table 4). The SAR studies on region 3 reveal significant sensitivity to OCR inhibition at this position and it is likely further optimisation is possible.

**Table 4:** 30–37 investigating the role of the group off the *N*-1 piperazine of 28 (region 3)

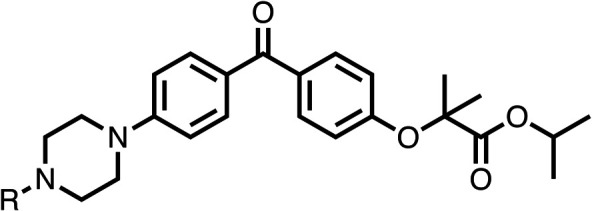 % OCR normalised to the DMSO controls											
Entry	R	100 μm	10 μm	Entry	R	100 μm	10 μm	Entry	R	100 μm	10 μm
30	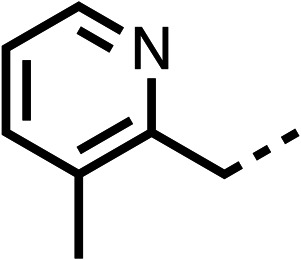	12.5 ± 6	19 ± 3	31	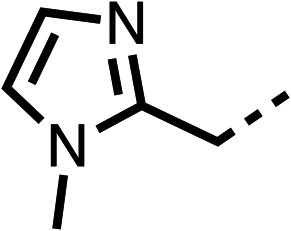	—	75	32	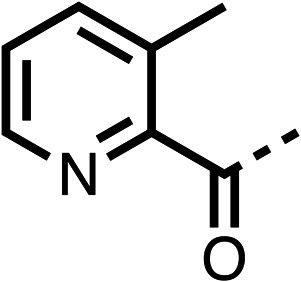	—	31 ± 12
33	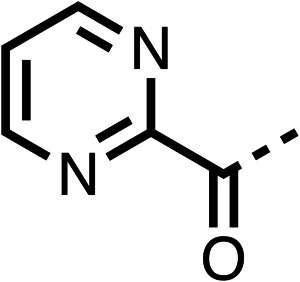	12 ± 5	73 ± 15	34	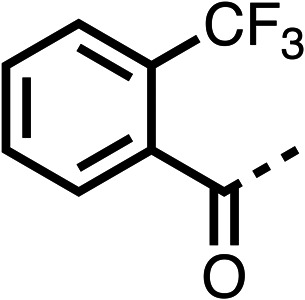	7 ± 4	6 ± 4	35	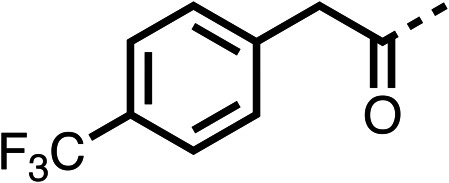	7 ± 4	6 ± 4
36	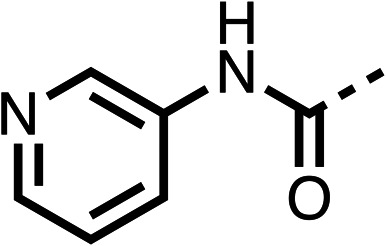	11 ± 8	32 ± 7	37	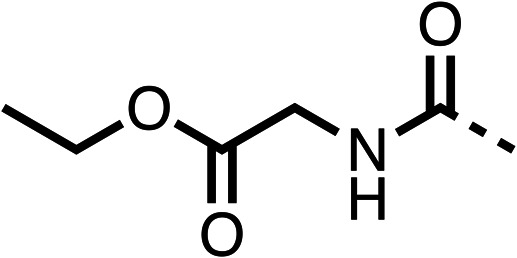	58 ± 8	88 ± 9				

We investigated the role of the ketone group (region 4) of fenofibrate (1) by replacing it with a secondary alcohol (38), a secondary amide (39), a secondary amine (40), tertiary amines (41–44), a tertiary amide (45) or urea (46, 47) functional groups (Table 5). To improve synthetic accessibility, we replaced the chloro-group of fenofibrate (1) with the dimethylamine group of 22, as they showed similar levels of efficacy towards OCR and would have improved aqueous solubility. The secondary alcohol (38) and the amide (39) produced a similar reduction of OCR as for fenofibrate (1, Table 5). The secondary (40) and the methyl tertiary amine (41) derivatives manifested improved inhibition (19% and 25% at 10 μM, respectively, Table 5) compared to fenofibrate (1). The ethyl amine (42) demonstrated a similar level of OCR inhibition (32% at 10 μM) compared to the methyl derivative (41) at 10 μM (Table 5). Introduction of larger groups such as the 3-methylpyridin-2-yl (43) and ethyl nicotinate (44) derivatives led to weaker inhibition (65% and 70% at 10 μM, respectively, Table 5) compared to 41. Addition of an acetyl group to 40 to give tertiary amide (45) resulted in substantial loss of OCR inhibition, at 10 μM reducing the OCR to only 89% (Table 5). The phenyl (46) and benzyl (47) urea derivatives, more potently inhibited OCR at 100 μM (18% and 7%, respectively). However, 46 and 47 did not inhibit OCR at 10 μM (90% and >100%, respectively). These results demonstrate that the ketone of fenofibrate (1) can be replaced with an amide (39), a secondary amine (40), or a tertiary amine (41) whilst maintaining or improving on OCR inhibition compared to fenofibrate (1).

**Table 5:** 38–47 investigating the role of the ketone of 1 (region 4)

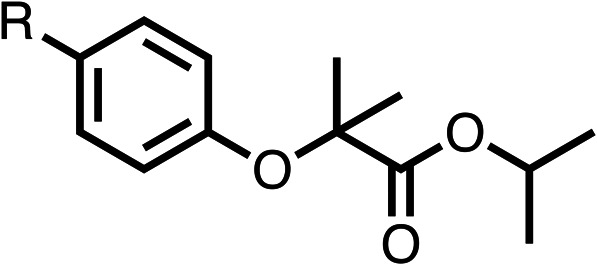 % OCR normalised to the DMSO controls											
Entry	R	100 μm	10 μm	Entry	R	100 μm	10 μm	Entry	R	100 μm	10 μm
38	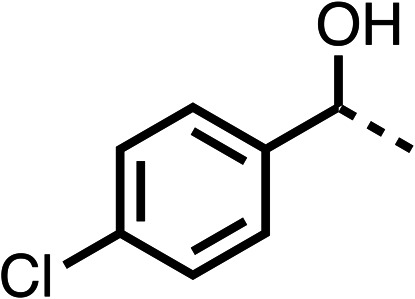	28 ± 1	96 ± 4	39	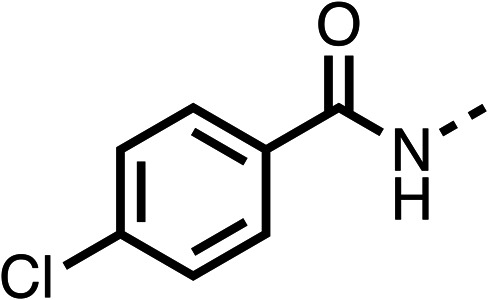	29 ± 6	73 ± 5	40	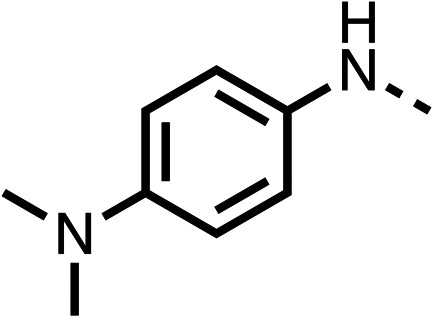	12 ± 6	19 ± 4
41	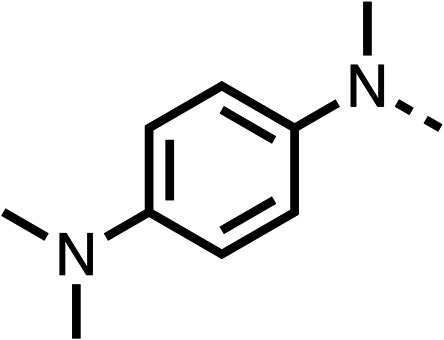	13 ± 13	25 ± 24	42	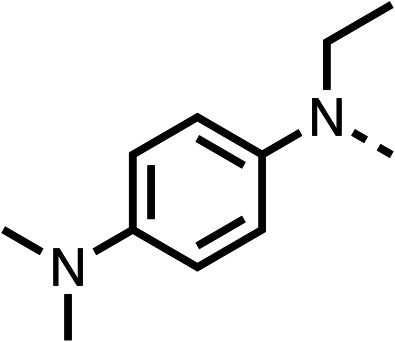	18 ± 2^a^	32 ± 2	43	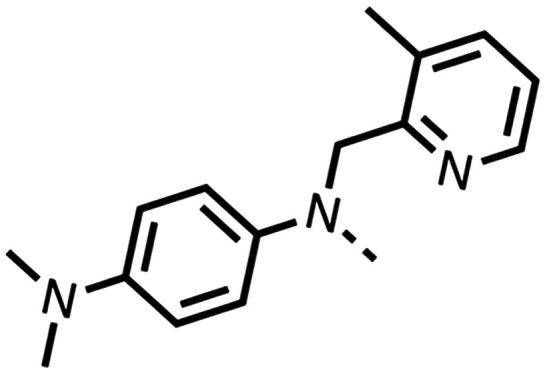	—	65 ± 13
44	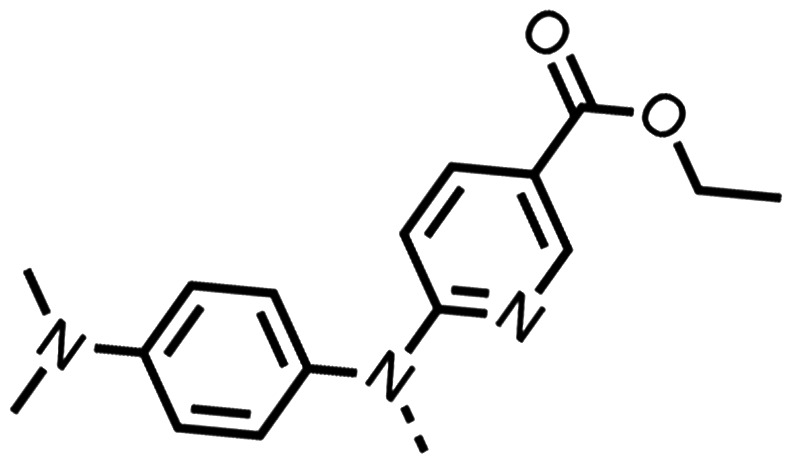	50	70	45	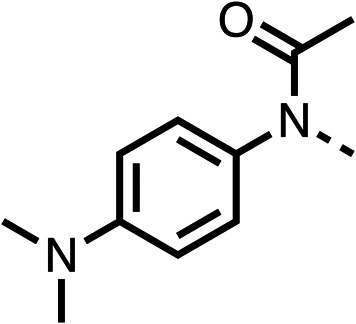	—	89 ± 11	46	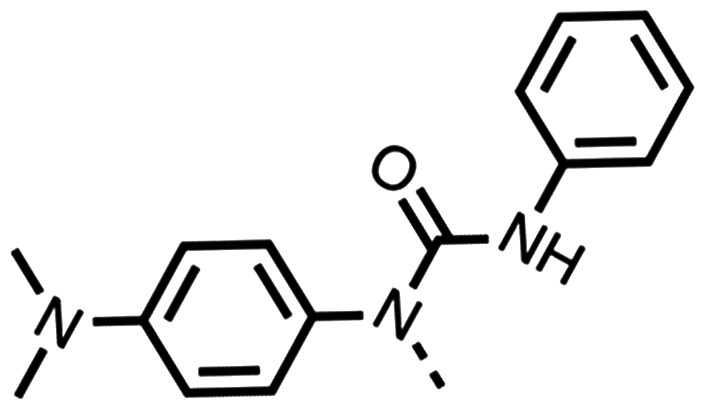	18 ± 6	90 ± 14
47	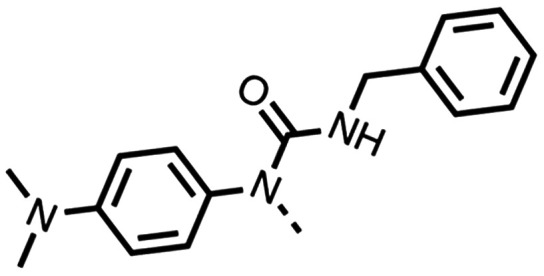	7 ± 9	>100								

a Tested at 30 μM.

**Fig. 2:**
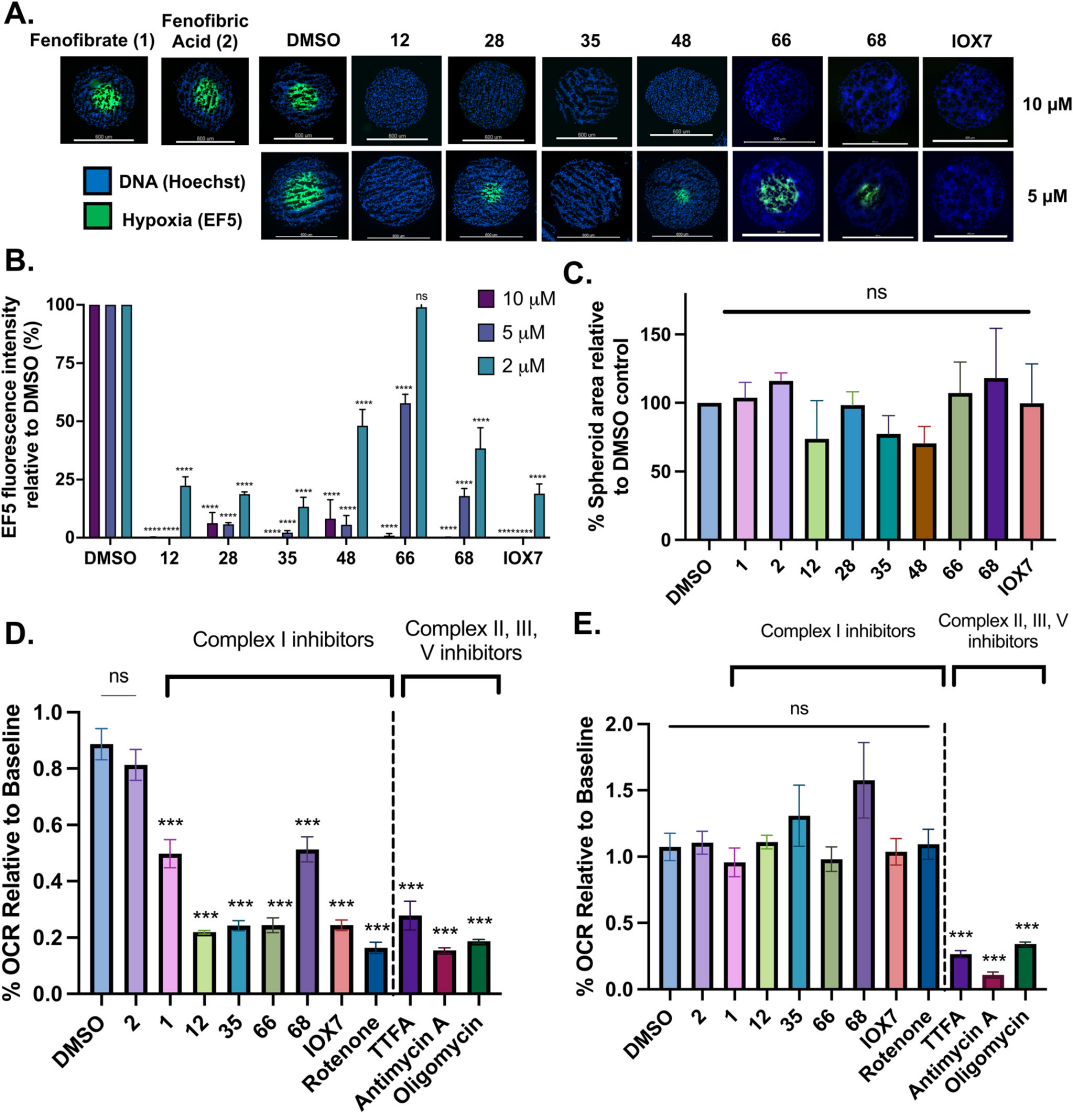
Fenofibrate derivatives abolish hypoxia in 3D spheroids through complex I inhibition. (A) Representative images of HCT116 spheroids treated with DMSO or the indicated compounds for 24 hours at 10 and 5 μM. Hypoxia was assessed by staining central spheroid sections for EF5 (green) and with DAPI as a nuclear counterstain (blue). Scale: 600 μm. (B) Hypoxia was assessed by relative EF5 fluorescence intensity in HCT116 spheroids treated with the indicated compounds for 24 hours at 10 μM, 5 μM, and 2 μM. 8–16 spheroids were analysed per treatment where data corresponds to an average ± SD of 2–3 independent experiments. (C) Percent mean spheroid area relative to DMSO controls of HCT116 spheroids treated with the indicated compounds for 24 hours at 10 μM from (B). (D) OCR relative to baseline measurements of permeabilised HCT116 cells 60 minutes after port injections of the indicated compounds, conducted in pyruvate-supplemented complex I-dependent medium or in (E) succinate- and rotenone-supplemented complex II-dependent medium. Data corresponds to an average ± SD from 2–4 independent experiments. One-way ANOVA with Bonferroni was performed for all the experiments (n.s. not significant, ****P < 0.0001, ***P < 0.001, **P < 0.01, *P < 0.05).

The 1st stage of SAR studies of fenofibrate (1) identified 12, 28 and 35 as manifesting potent OCR inhibition. 12, 28 and 35 were thus assessed in HCT116 spheroids measuring the reduction of hypoxia. 12, 28 and 35 were initially screened at 10 μM, a concentration where fenofibrate (1) is no longer effective at alleviating hypoxia (Fig. 2A and B). 12, 28 and 35 abolished spheroid hypoxia at 10 μM. 12 completely alleviated hypoxia at 5 μM, while a large reduction in hypoxia was observed at the same concentration in spheroids treated with 28 and 35 (Fig. 2A and B). 12, 28 and 35 demonstrated low levels of toxicity as measured by changes in spheroid cross-sectional area (Fig. 2C). Confirmation of mitochondrial complex I-specific inhibition was conducted using membrane-permeabilised HCT116 cells in medium either supplemented with pyruvate, a complex I-specific substrate, or succinate, a complex II-specific substrate. Complex I-specific respiration, but not complex II-specific respiration, was inhibited by 1, 28, 35 and the known complex I inhibitor rotenone (Fig. 2D and E). As a positive control, inhibitors of downstream complexes (complex II, III, and V) demonstrate inhibition of both complex I- and complex II-specific respiration (Fig. 2D and E).

The 2nd stage of SAR studies, focused on combining modifications discovered in stage one, aiming to replace the isopropyl ester of fenofibrate (1) to avoid esterase metabolism and reduce hydrophobicity whilst maintaining OCR inhibition in the 1–5 μM inhibitor range. We thus combined modification to the isopropyl ester of fenofibrate (1) with either a cyclohexyl amide (12) or dimethyl amide (14) and the replacement of the chloride of fenofibrate (1) with a piperidine group with either an *N*-1 acetyl (27) or Cbz (28) group.

48, which contains a combination of substituents from 12 and 28, potently inhibited OCR and 5 μM (24% at 10 μM and 37% at 5 μM, Table 6). In the spheroid assay, 48 significantly reduced hypoxic regions at 5 and 10 μM, with moderate reduction at 2 μM (50% reduction) (Fig. 2B). Replacement of the Cbz group of 48 with an acetyl group (49) resulted in weaker inhibition of OCR (59% at 10 μM, Table 6), as did replacing the cyclohexyl group of 49 with a dimethylamine group (50) (73% at 10 μM, Table 6). Re-addition of the Cbz group to 50 to give 51 restored OCR inhibition (34% at 10 μM and 39% at 5 μM), showing that a reduction in hydrophobicity whilst maintaining OCR potency could be achieved, as supported by the ethyl amide (52) and ketone (53) derivatives that were strong OCR inhibitors (20% and 37% at 10 μM, respectively, Table 6). Replacing the ketone of 29 with an amide (54) resulted in significant loss of OCR inhibition (75% at 10 μM) with conversion of the Cbz group of 54 to an acetyl group (55) achieving a similar level of OCR inhibition (70% at 10 μM, Table 6).

**Table 6:** Inhibition of the oxygen consumption rate (OCR) by fenofibrate (1) derivatives from stage 2 SAR studies. 48–53 were made to investigate the combination of regions 1 and 3 of fenofibrate (1). 54 and 55 were made to investigate the combination of regions 3 and 4. 56–59 were made to investigate the combination of regions 1, 2 and 3. 60–69 were made to investigate combination of regions 1, 3 and 4

% OCR normalised to the DMSO controls								
Entry	Area 1	Area 2	Area 3	Area 4	100 μm	30 μm	10 μm	5 μm
48	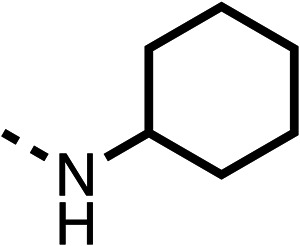	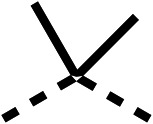	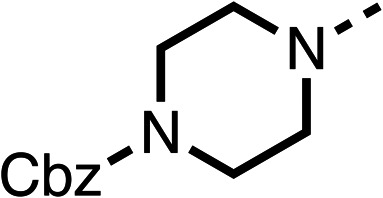	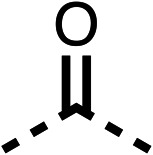	13 ± 2	17 ± 1	24 ± 5	37 ± 3
49	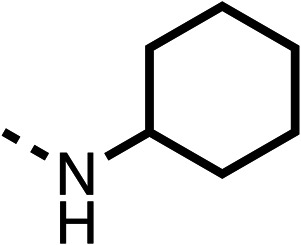	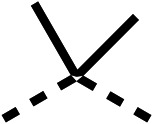	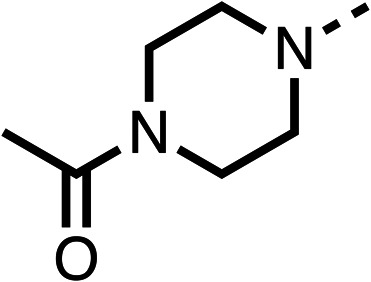	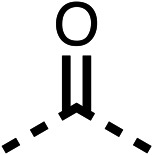	—	44 ± 3	59 ± 3	80 ± 4
50	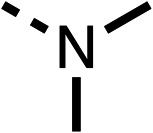	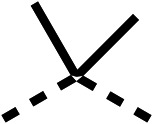	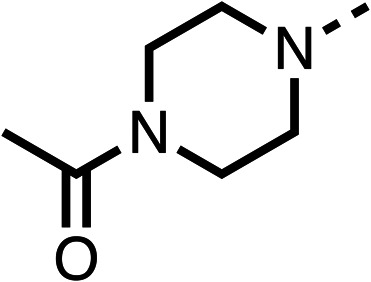	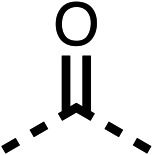	—	65 ± 5	73 ± 5	79 ± 4
51	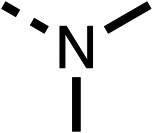	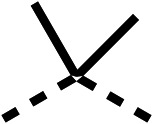	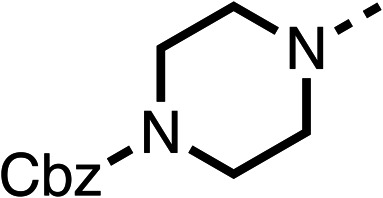	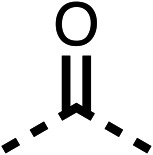	—	13 ± 2	34 ± 5	39 ± 4
52	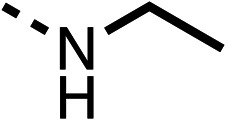	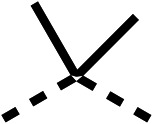	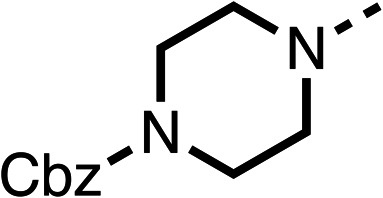	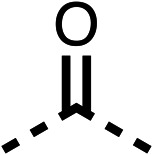	2 ± 2	—	20 ± 6	—
53	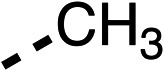	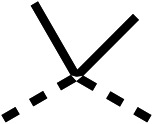	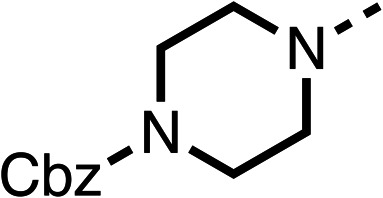	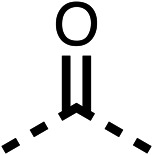	21 ± 2	—	37 ± 12	—
54	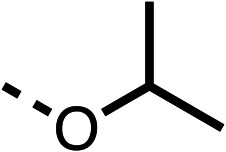	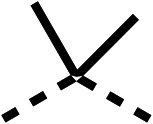	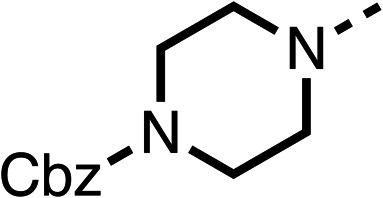	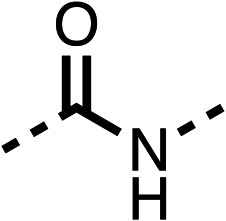	29 ± 5	49 ± 2	75 ± 7	94 ± 3
55	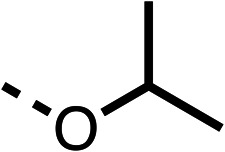	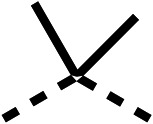	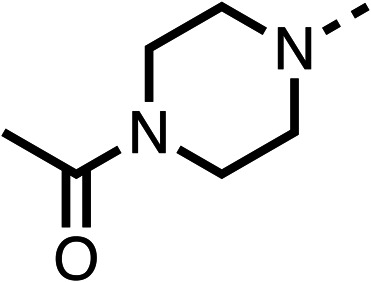	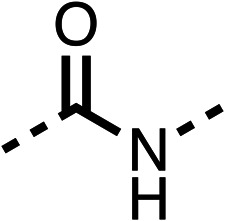	—	41 ± 2	70 ± 2	89 ± 2
56	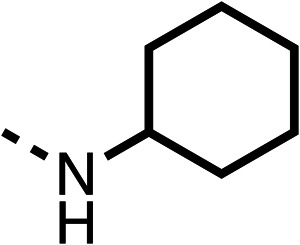	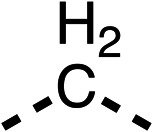	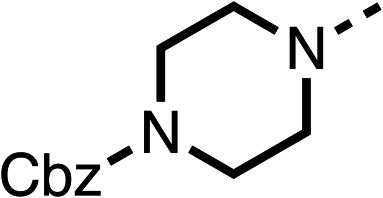	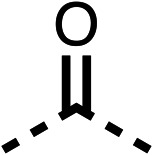	—	25 ± 3	47 ± 6	27 ± 4
57	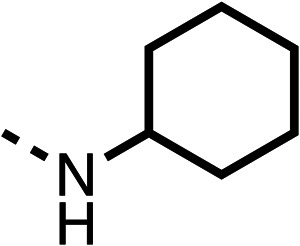	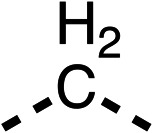	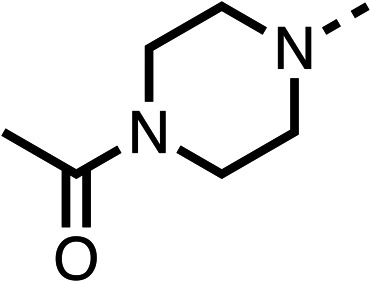	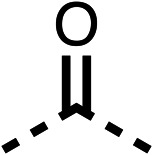	—	15 ± 2	47 ± 2	72 ± 2
58	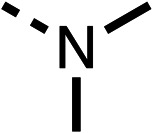	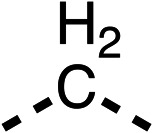	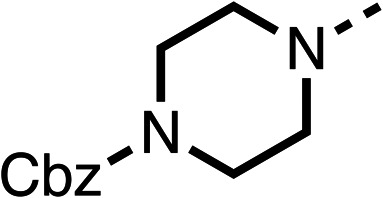	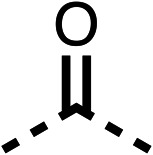	—	46 ± 2	63 ± 5	74 ± 4
59	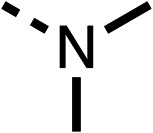	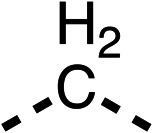	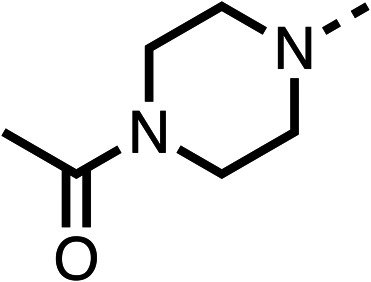	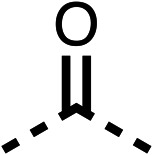	—	>100	>100	90 ± 6
60	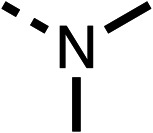	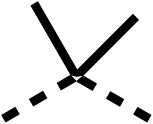	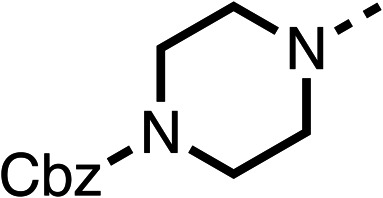	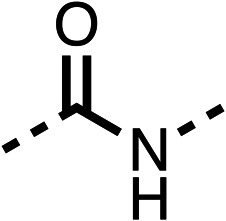	—	20 ± 3	34 ± 3	>100
61	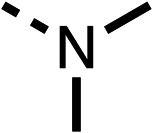	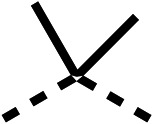	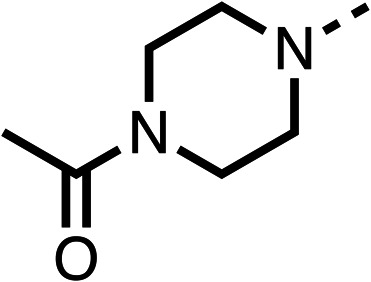	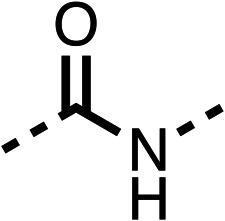	—	86 ± 4	>100	>100
62	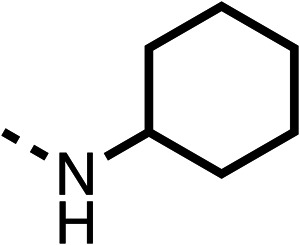	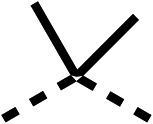	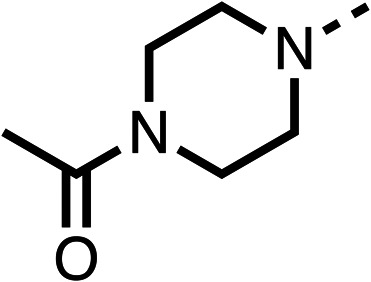	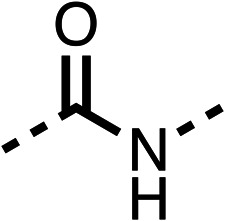	—	49 ± 4	93 ± 3	90 ± 2
63	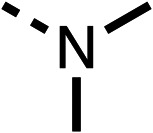	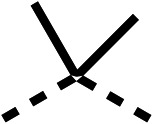	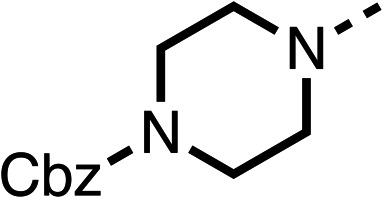	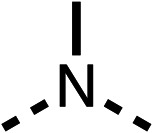	—	12 ± 1	24 ± 2	41 ± 4
64	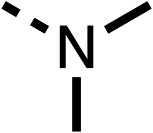	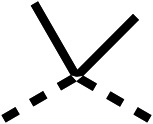	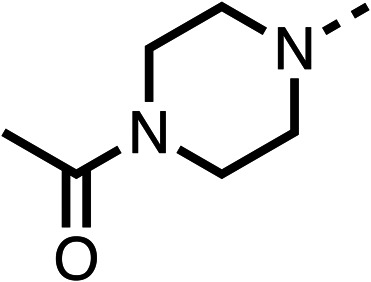	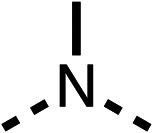	—	84 ± 3	92 ± 7	75 ± 7
65	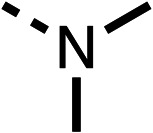	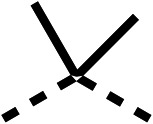	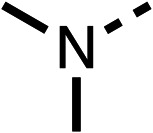	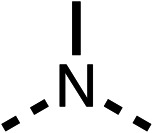	—	65 ± 5	93 ± 5	78 ± 8
66	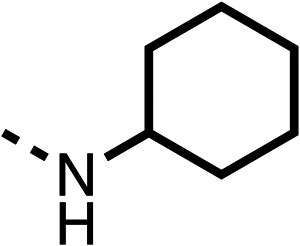	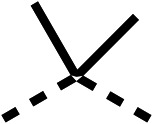	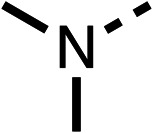	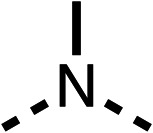	—	15 ± 2	48 ± 6	58 ± 5
67	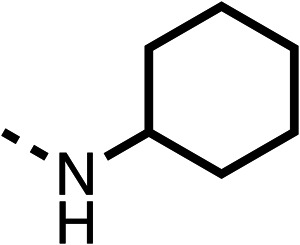	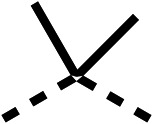	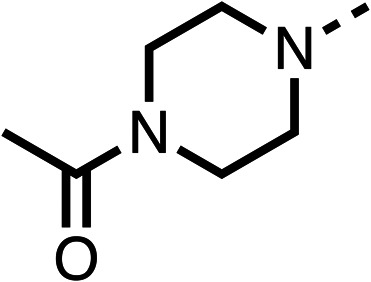	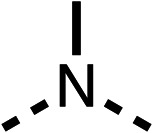	—	39 ± 3	82 ± 3	72 ± 9
68	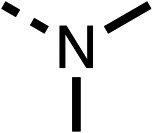	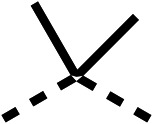	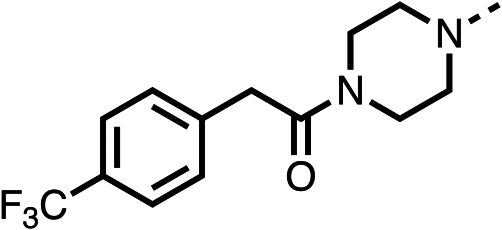	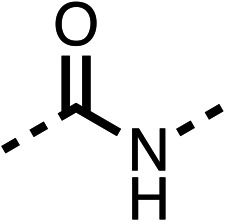	—	50 ± 5	72 ± 2	57 ± 2
69 (**IOX7**)	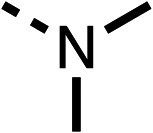	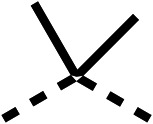	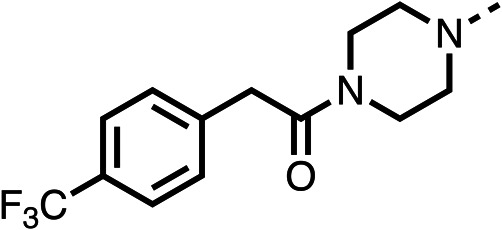	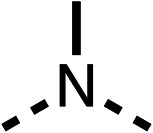	—	4 ± 0.3	29 ± 9	22 ± 2

We investigated whether the dimethyl group (region 2) is required for OCR inhibition by combining the preferred groups from regions 1 and 3. The dimethyl group of 48–51 was replaced with a methylene linker (56–59 (Table 6)). Cyclohexyl amide derivatives with either an Cbz (56) or acetyl (57) protected piperazine in region 3 moderately inhibited OCR at 10 μM (47% and 47%, respectively). At 5 μM, 56 potently inhibited OCR (27%) whereas 57 weakly inhibited OCR (72%). The Cbz dimethyl amide derivative 58 was a weak inhibitor of OCR at 10 μM (63%) whereas the *N*-1 acetyl derivative 59 did not inhibit OCR at any tested concentration (Table 6).

We then investigated whether the potency of OCR inhibition of 51 was maintained on replacing the ketone with an amide (60) or tertiary amine (63). 60 gave similar levels of OCR inhibition at 10 μM (34%, Table 6) to 51 (34%, Table 6). Replacement of the Cbz group of 60 with an acetyl (61) group resulted in loss of OCR inhibition at 30 μM (86%, Table 6). With the aim of recovering OCR inhibition, we replaced the dimethyl amide of 61 with a cyclohexyl amide (62); however, 62 only moderately inhibited OCR (49% at 30 μM, Table 6). The tertiary amine (63) resulted in a stronger inhibition at 10 μM (24%, Table 6) compared to 51 (34%). Modifications in regions 1 and 3 of 63 resulted in a similar loss of OCR inhibition as observed with the amides (60–62). Replacement of the Cbz group (63) with an acetyl group (64) resulted in near complete loss of OCR inhibition (84% and 92% at 30 μM and 10 μM respectively, Table 6). Replacement of the *N*-1-acetyl-piperazine group with a dimethylamine group (65) resulted in a small improvement in OCR inhibition at 30 μM (65%, Table 6). Modification of the dimethylamide group (65) to a cyclohexyl amide (66) resulted in an improvement of OCR inhibition at 30 μM and 10 μM concentrations (15% and 48% respectively, Table 6). However, the *N*-1-acetyl-piperazine derivative of 66 (67) was a poor OCR inhibitor at 10 μM (82%, Table 6).

To complete the second stage of SAR studies, the Cbz group of 60 and 63 was modified to the 4-trifluoromethyl benzyl amide of 35 to give amide (68) and tertiary amine derivatives (69, **IOX7**). 68 moderately inhibited OCR (57 at 5 μM, Table 6), an improvement over the Cbz derivative (60). 69 was a more potent OCR inhibitor (22% at 5 μM) compared to the Cbz derivative 63 (41% at 5 μM, Table 6). In the spheroid assay 66, 68 and 69 abolished hypoxia at 10 μM (Fig. 2A and B). At 5 μM, 66 partially reduced the hypoxic region in spheroids, whereas this effect was more pronounced with 68 and 69 (Fig. 2A and B). At 2 μM, 68 and 69 significantly reduced the hypoxic region (60% and 80% reduction, respectively, Fig. 2B). The cross-section surface area of spheroids treated with 66, 68 or 69 at 10 μM were equal to the DMSO control indicating low toxicity (Fig. 2C). 66, 68, and 69 were shown to specifically inhibit complex I (Fig. 2D), confirming their mechanism of action.

## Conclusion

The SAR studies reveal that fenofibrate (1) is an excellent scaffold for developing a well-tolerated, inhibitor of complex I which can modify tumour hypoxia. SAR studies sought to overcome its esterase-mediated hydrolysis of fenofibrate (1), while improving upon its potency for OCR inhibition. Modifications of regions 1, the isopropyl ester; 3, the chloro group; and 4, the ketone group, led to improved OCR inhibition and abolishment of hypoxic regions in spheroids at low concentrations (12 and 35). However, these modifications resulted in solubility issues due to the hydrophobic nature of these compounds. In addition, 35 contained an isopropyl ester and is therefore liable to esterase hydrolysis. To resolve these issues, we combined the SAR studies from regions 1, 3, and 4 resulting in IOX7 (69), which has improved solubility whilst maintaining potent OCR inhibition.

Targeting cellular respiration is a potential treatment for reducing hypoxia to improve resistance to other treatments, particularly radiotherapy, and can also be used to target cancers that are sensitive to OXPHOS inhibition. Recently developed complex I inhibitors have failed in clinical trials (BAY87-2243, ASP4132, and IACS-010759) when used as monotherapies in cancers predicted to be sensitive to OXPHOS inhibition.^21,29^ The failures of these three compounds was due to dose-limiting toxicities, which were proposed to be due the high potency of OXPHOS inhibition, which are ∼1000-fold greater than the compounds presented here.^22,30^ To date only three OXPHOS inhibitors, metformin, atovaquone, and papaverine, all of which are re-purposed drugs, have been assessed as hypoxia modifiers in clinical trials with radiotherapy. Studies with metformin, a weak complex I inhibitor requiring millimolar concentrations, have failed likely due to lack of on-target potency and poor drug uptake.^23,24,31^ Trials with the moderately potent OXPHOS inhibitors papaverine (complex I inhibitor) and atovaquone (complex III inhibitor) are ongoing. The novel compounds developed in our work appear to have a suitable potency for OXPHOS inhibition in cells, in the micromolar range, and can successfully eliminate hypoxia in 3D models without inducing cytotoxicity, demonstrating their potential as non-toxic and effective hypoxia modifiers for *in vivo* studies.

Overall, the results presented here demonstrate the feasibility of utilising the fenofibrate (1) scaffold to develop complex I inhibitors with optimised potency and cytotoxicity profiles. Ongoing work involves translating the results *in vivo* by assessing the safety and efficacy of IOX7 in murine models.

## Author contributions

TA performed initial screen identifying fenofibrate as an oxidative phosphorylation inhibitor. JPHM developed chemical modification strategy. JPHM, SA, and TKW synthesized the compounds. TA and JTTC performed *in vitro* experiments characterizing fenofibrate as a metabolic radiosensitizer. NM, JTTC, RP, ET and GRB conducted *in vitro* experiments characterizing fenofibrate derivatives. JPHM and NM wrote the inital manuscript draft. GRB, GSH, CJS revised the manuscript and supervised the project. GSH and CJS conceptualized the project.

## Conflicts of interest

There are no conflicts of interest to declare.

## Supplementary Material

MD-OLF-D5MD00742A-s001

## Data Availability

The data supporting this article, including chemical synthesis protocols and NMR data have been included as part of the supplementary information (SI). Supplementary information is available. See DOI: https://doi.org/10.1039/d5md00742a.
