# Supplementary material for: Structure-guided optimisation of fenofibrate-derived oxidative phosphorylation inhibitors to modify tumour hypoxia
Source: RSC Med Chem. 2026 Apr 28;17(6):2925–35. doi: 10.1039/d5md00742a (PMC13123558; doi:10.1039/d5md00742a)
Supplement: MD-017-D5MD00742A-s001 [file MD-017-D5MD00742A-s001.pdf]

Supplementary Information (SI) for RSC Medicinal Chemistry.
This journal is © The Royal Society of Chemistry 2026

Supplementary Information

Structure-guided optimisation of fenofibrate-derived oxidative phosphorylation inhibitors modify tumour hypoxia

James P. Holt-Martyn[2†], Nicole Machado[1†], James T. T. Coates[1], Rathi Puliyadi[1], Thomas Ashton[1], Elysia Traynor[1], Samina Aslam[2], Thomas. K Wise[2], Gonzalo Rodriguez-Berriguete[1*], Christopher J. Schofield[2*], and Geoff S. Higgins[1*]

[1]Department of Oncology, University of Oxford, Oxford OX3 7DQ, United Kingdom.

[2] Chemistry Research Laboratory, Department of Chemistry and the Ineos Institute for Antimicrobial Research, University of Oxford, Oxford OX1 3TA, United Kingdom.

[†] These authors have contributed equally
* Corresponding authors

Materials and Methods
Cell culture

All cell lines were from the American Type Culture Collection (ATCC). STR profiling (DNA Diagnostics Centre, UK) and mycoplasma testing (Lonza) are conducted routinely for these cell lines. Cells were cultured at 37C with 5% CO_2 in Dulbecco's modified Eagle's medium (DMEM, Sigma) supplemented with 10% foetal bovine serum (FBS, Lonza) and 1% Penicillin-Streptomycin (PenStrep, Sigma).

OCR Seahorse Assays

The oxygen consumption rate (OCR) was measured using an automated Seahorse XF96 Analyzer (Agilent). For fenofibrate (**1**) (Sigma) dose response curves, cell culture media of cells seeded in 96-well XF Cell Culture plates (Agilent) was replaced with pre-warmed Seahorse Assay Medium (Agilent) with 5mM glucose (Sigma), 5mM sodium pyruvate (Sigma) and 4mM L-glutamine (Sigma) to a final pH of 7.4; the mixture and placed in a non-CO_2 incubator for 40-60 minutes prior to assay. Following three baseline measurements, port injections of fenofibrate (**1**) were conducted at varying concentrations and measurements are recorded for up to 2.5 hours. For screening derivative compounds, HCT116 or FaDu cells are treated for 24 hours with respective compounds prior to replacing medium with pre-warmed Seahorse Assay Medium formulated as aforementioned and placed in a non-CO_2 incubator for 40-60 minutes before taking an average of three OCR measurements. Immediately after all assays, the cell plates are fixed with 4% aqueous paraformaldehyde (PFA_{aq}), a nuclear stain was conducted with 1 μg/mL Hoechst 33258 (Sigma) and cells were counted by fluorescent images taken with Celigo Imaging Cytometer (Nexcelom Bioscience).

For complex I and II dependant respiration assays, HCT116 cells were seeded in 96-well XF Cell Culture plates (Agilent) overnight. Cell culture media was replaced with 3X MAS buffer, supplemented with 4mM adenosine 5-diphosphate (Sigma, #01905), and 1nM XF PMP (Agilent, #102504-100) either 10mM pyruvic acid (Sigma, #107360), 1mM malic acid (Sigma, #240176) for complex I dependant respiration or 10mM succinate acid (Sigma, #S9512) and 2μM rotenone (Sigma, #R8875) for complex II dependant respiration as previously described.(1) Following three baseline measurements, port injections of test compounds or known inhibitors were conducted. Fenofibrate (**1**), fenofibric acid (**2**) (Sigma) and derivative compounds are injected at 30μM. The known inhibitors used for the assay were 2μM rotenone for complex I, 800μM 2-thenoyltrifluoroacetone (TTFA) (Cayman chemical company,

#15517) for complex II, 2μM antimycin A (Sigma, #A8674) for complex III and 2μM oligomycin (Sigma, # 75351) for complex V.

Clonogenic assays

Cells were plated as single cells in 6-well plates, where seeding densities were optimised for each cell line; increasing cell numbers were used for higher ionising radiation (IR) doses to account for cell death. Following treatment with fenofibrate (**1**), cells were irradiated in a Cs-137 irradiator (GSR D1 from Gamma Service; dose rate 1.2 Gy/min) and colonies were grown for 8 to 14 days, stained with crystal violet, and counted using the Gelcount automated colony counter (Oxford Optronics). The plating efficiency (PE = average colony number/cells plated) and the surviving fraction (SF = $PE_{IR\ Dose}/PE_{0\ Gy}$) at a given IR dose was calculated. Survival data were fitted according to a linear quadratic equation.

Spheroid Assays

FaDu spheroids were grown using the liquid overlay technique, and HCT116 and H1299 spheroids were grown in Ultra Low Attachment U-bottom plates (Grenier Bio-One, #650970). Cells were seeded high-glucose (4g/L) DMEM (10% FBS 1% PenStrep) and grown until they reached an average diameter of 550-650μm, which was confirmed and recorded with a GelCount Colony Counter (Oxford Optronix). After treatments, spheroids are incubated in 200 μM EF5 (Merk Millipore, #152721-37-4) for 6 hours and fixed in 4% PFA_{aq} overnight at 4°C prior to cryoprotecting spheroids in 30 % sucrose for a minimum of 2.5 hours at 4˚C. Spheroids were embedded in O.C.T. Compound (VWR, TissueTek) and stored in -80°C freezer until sectioning and staining.

Immunohistochemistry

All sectioning was conducted using a cryostat (Leica Biosystems) at -22°C. Spheroid sections were taken at 5 μm and stored in -20°C until processed. Sections were hydrated in 1 x PBS, 0.3% Tween 20 (Sigma) (1 x PBST), blocked with TNB blocking reagent (PerkinElmer, #FP1020) for 60 minutes, washed with 1 x PBST and incubated with 70 μL of 66 μg/mL anti-EF5 488 antibody (Merck Millipore, #EF5010 or #EF5012) overnight at 4°C. Samples were washed 3 times with 1 x PBST, incubated in 1 μg/mL Hoechst 33258 for 5 minutes and mounted on slides with ProLong Diamond Antifade (Life Technologies). Slides were imaged using fluorescence microscopy (Nikon 90i, Nikon) and analysed with Imaris Microscopy

Image Analysis Software (Oxford Instruments). For spheroid analyses, the Imaris cell detection function was applied to outline spheroids, where spheroid areas were classified as cells and hypoxic cores classified as nuclei. Samples that were folded, broken, and deformed were excluded from analysis. For all images, the same look up table (LUT) settings were applied to all fluorescent channels for each experiment.

The following formula was used where S represents individual spheroids.

Mean EF5 fluorescence intensity relative to control:

$$\frac{Average_{Treatment}\left(\frac{(Area\ of\ EF5\ x\ EF5\ intensity\ per\ S)}{(Total\ area\ per\ S)}\right)}{Average_{Control}\left(\frac{(Area\ of\ EF5\ x\ EF5\ intensity\ per\ S)}{(Total\ area\ per\ S)}\right)}\ x\ 100\%$$

Statistical Analysis

Statistical analysis was performed with Prism 10 software (GraphPad Software Inc.). All values are presented as mean ±s.d. Two-way ANOVAs with Bonferroni post-hoc correction were used on clonogenic survival curve analysis. For all other experiments, one-way ANOVAs were performed to assess statistical significance with Bonferroni post-hoc correction. P values < 0.05 were considered significant. All *in vitro* experiments were repeated between 2-4 times as indicated. The linear quadratic model, $S = \exp(\alpha D - \beta D^2)$, was used to fit clonogenic survival graphs, with S denoting survival and D the IR dose in Gy. GraphPad Prism (RRID:SCR_000306) was used for all statistical calculations and curve fitting.

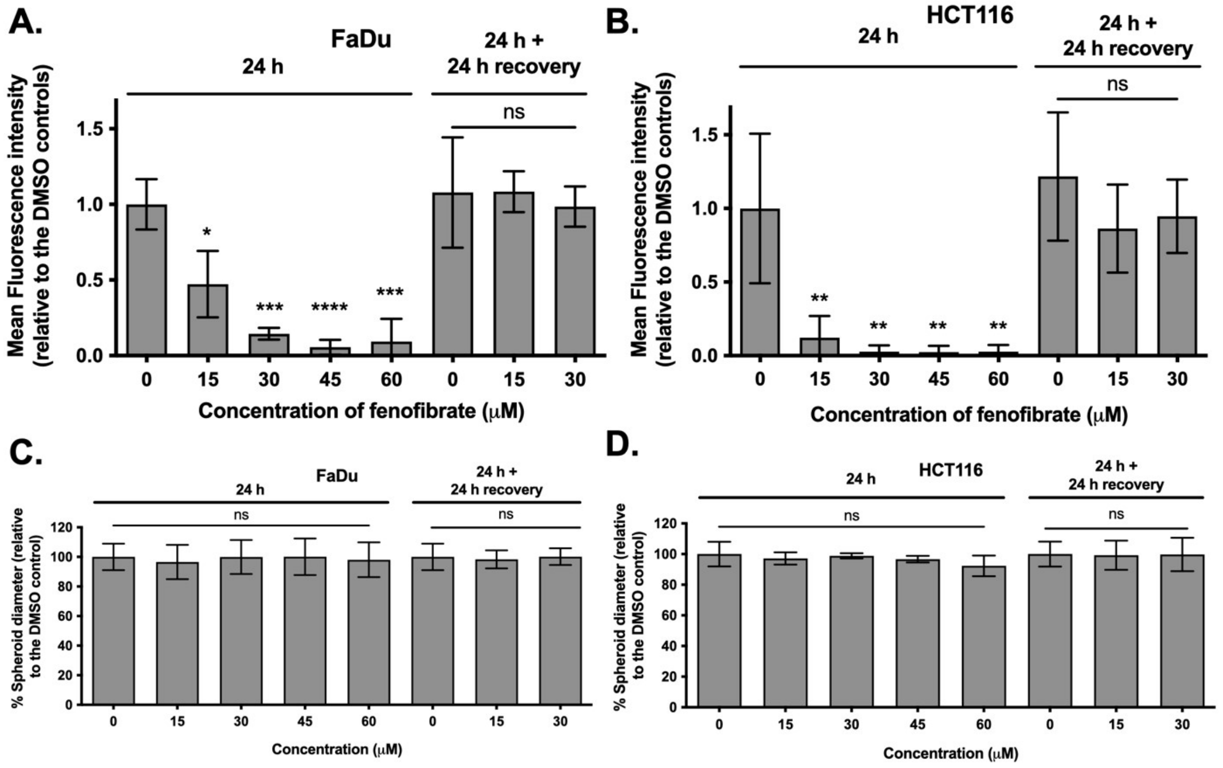

Supporting Figure S1. Fenofibrate (1) alleviates spheroid hypoxia without affecting spheroid size

- **(A)** Mean EF5 fluorescence intensity relative to DMSO controls of FaDu spheroids treated with DMSO or fenofibrate (1) either for 24 hours, or for 24 hours followed by recovery for 24 hours in drug-free medium, as indicated.
- **(B)** Same as (A) with HCT116 spheroids.
- **(C)** Percent spheroid diameter relative to DMSO control in FaDu spheroids from **(A)**.
- **(D)** Percent spheroid diameter relative to DMSO control in FaDu spheroids from **(B)**.

Data corresponds to an average ± SD from three independent experiments. One-way ANOVA with Bonferroni was performed for all the experiments (n.s. not significant, ****P<0.0001, ***P<0.001, **P<0.01, *P<0.05).

Synthetic Routes

A)

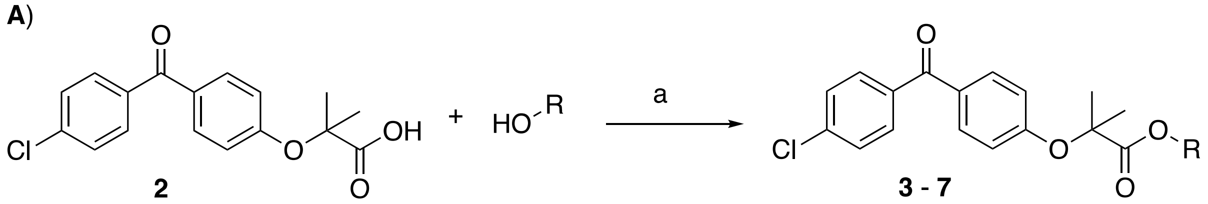

B)

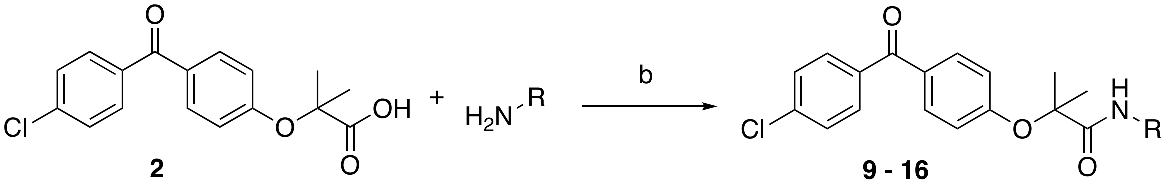

Supporting Figure S2. Synthesis of derivatives 3-7 and 9-16. SAR series investigating the isopropyl group (Region 1 of fenofibrate (1)) [a,b]**. A**) Compounds **3-7**, were prepared in one step from fenofibric acid (**2**) using the coupling reagent COMU and the corresponding alcohol (yields: 22% - 63%). **B**) The amides (**9** - **16**) were synthesised using the coupling reagent HATU and the corresponding amine (yields: 33% - 89%). [a] Reagents and conditions: (a) COMU, DIPEA, DMF, rt, 22 - 63%; (b) HATU, DIPEA, DMF, rt, 33 - 89%. [b] Chemical structures of R groups are shown in Table 1.

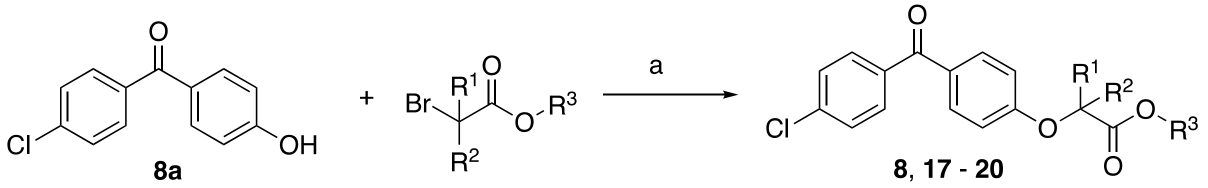

Supporting Figure S3. Synthesis of derivatives 8, 17–20. SAR series investigating the role of the dimethyl group (Region 2 of fenofibrate (1))[a,b]. Compounds **8**, **17-20** were synthesised in one step *via* an S_N2 reaction using the commercially sourced (4-chlorophenyl)(4-hydroxyphenyl)methanone (**8a**) with the corresponding α-halo carbonyl (yields: 16% - 94%). [a] Reagents and conditions: (a) Cs_2CO_3, DMAc, 80°C, 16 - 94%. [b] Chemical structures of R groups are shown in Table 2.

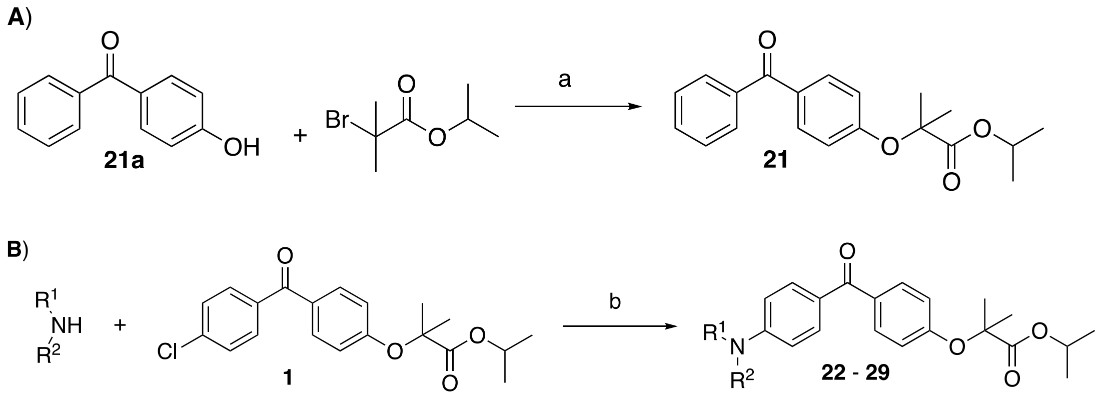

Supporting Figure S4. Synthesis of derivatives 21-28. Initial SAR series investigating the chloro group (Region 3 of fenofibrate (1))[a,b]. A) **21** was synthesized in one step *via* an S_N2 reaction using commercially sourced (4-chlorophenyl) (4-hydroxyphenyl) methanone (**21a**) and isopropyl 2-bromo-2-methylpropanoate (yield 16%). **B**) Compounds **22-28** were prepared in one step via a palladium catalysed amination with **1**. The corresponding amine and specific ligand depending on whether a 1° or 2° amine was used, i.e. Brettphos for primary amines and RuPhos for secondary amines (yields: 17% - 86%). [a] Reagents and conditions: (a) Cs_2CO_3, DMAc, 80°C, 25%; (b) Pd Ligand (BrettPhos for 1° amines and RuPhos for 2° amines), Cs_2CO_3, ^{t}BuOH, 80°C, 17 - 86%. [b] Chemical structures of R groups are shown in Table 3.

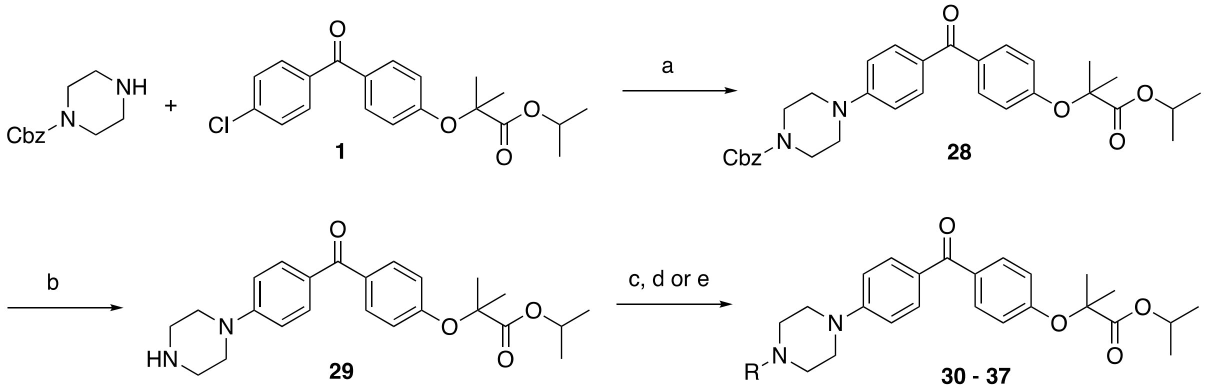

Supporting Figure S5. Synthesis of derivatives 29-37. SAR series investigating the role of the group off the *N*-1 piperazine of compound 28 (Region 3 of fenofibrate (1)) [a,b]. Compounds **29** – **37** were prepared in three steps. **28** was prepared *via* a palladium catalysed amination with the commercially available **1** and benzyl piperazine-1-carboxylate. **28** underwent a palladium catalysed hydrogenation to the corresponding secondary amine **29**. The final step varied dependent on which functional group was used. The tertiary amines, **30** and **31**, were prepared *via* a reductive amination using sodium borohydride and the corresponding

aldehyde (yield 45% and 37% respectively). The amide derivatives, **32-35**, were prepared using the coupling reagent HATU and the corresponding carboxylic acid (yields: 56% - 63%). The urea derivatives, **36** and **37**, were prepared using the corresponding isocyanate (66% and 72% respectively).[a] Reagents and conditions: (a) RuPhos Pd G3, Cs_2CO_3, ᵗBuOH, 80°C, 34%; (b) H_2, Pd/C, MeOH, rt, 86%; (c) HATU, DIPEA, DMF, rt, 56 - 68%; (d) aldehyde, $NaBH(OAc)_3$, CH_2Cl_2, rt, 16hr, 46%; (e) isocyanate, DIPEA, DMAc, rt, 16hr, 36 - 71%. [b] Chemical structures of R groups are shown in Table 4.

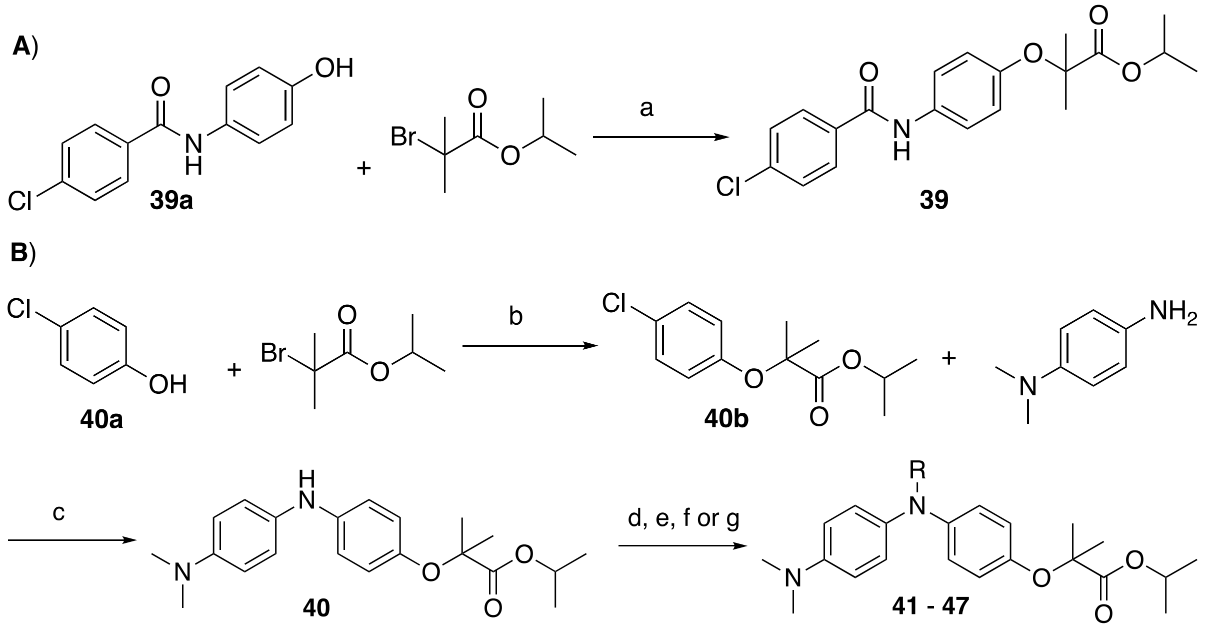

Supporting Figure S6. Synthesis of Compounds 39–47. SAR series investigating the role of the ketone (Region 4 of fenofibrate (1))[a,b]. A) The amide derivative **39** was prepared *via* S_N2 reaction with the commercially available **39a** and isopropyl 2-bromo-2-methylpropanoate (yield 56%). **B**) **40** was prepared in 2 steps from commercially sourced 4-chlorophenol (**40a**). Etherification of **40a** with the isopropyl 2-bromo-2-methylpropanoate to give **40b** (yield 76%). A palladium catalysed amination with *N*1,*N*1-dimethylbenzene-1,4-diamine gave **40** (yield 65%). The final step for **41–47** varied depending on the functional group. The tertiary amine derivatives, **41–44**, were synthesised via a reductive amination of **40** with the corresponding aldehyde (yields: 42% – 71%). **44** was prepared via a palladium catalysed amination with ethyl 6-chloronicotinate and **40** (27%). The amide derivative **45** was prepared with using acetyl chloride (76%). The urea derivatives, **46** and **47**, were prepared using the corresponding isocyanate with **40** (yield 15% and 19% respectively). **38** was from Santa Cruz Biotechnology. [a] Reagents and conditions: (a) Cs_2CO_3, DMAc, 80°C, 56%; (b) Cs_2CO_3, DMAc, 80°C, 76%;

(c) BrettPhos Pd G3, Cs_2CO_3, ^tBuOH, 65%; (d) aldehyde, $NaBH(OAc)_3$, CH_2Cl_2, rt, 16hr, 42 - 71%; (e) RuPhos Pd G3, Cs_2CO_3, ^tBuOH, 80°C, 27%; (f) AcCl, DIPEA (76%); (g) Isocyanate, DIPEA, DMAc, rt, 16hr, 15 and 19%. [b] Chemical structures of R groups are shown in Table 5.

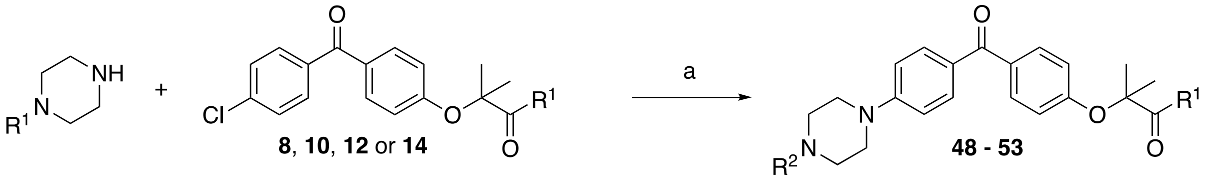

Supporting Figure S7. Synthesis of derivatives 48–53. SAR series investigating the combination of regions 1 and 3 of fenofibrate (1)[a,b]. The corresponding ketone (**8**) or amide (**10**, **12** or **14**) derivatives were used. Compounds **48–53** were synthesised *via* a palladium catalysed amination using RuPhos Pd G3 and the corresponding substituted piperazine derivative to give the target compounds (yields ranging from 34% - 58%). [a] Reagents and conditions: (a) RuPhos Pd G3, Cs_2CO_3, ^tBuOH, 80°C, 34% - 58%. [b] Chemical structures of R groups are shown in Table 6.

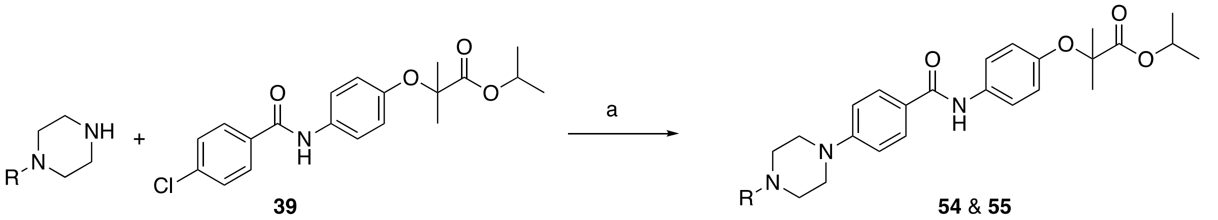

Supporting Figure S8. Synthesis of derivatives 54 and 55. SAR series investigating the combination of regions 3 and 4 of fenofibrate (1) [a,b]. Compounds **54** and **55** were prepared in one step from t amide **39** *via* the palladium catalysed amination using RuPhos Pd G3 and the corresponding substituted piperazine (yield 31% and 15% respectively). [a] Reagents and conditions: (a) RuPhos Pd G3, Cs_2CO_3, ^tBuOH, 80°C, 15 and 31%. [b] Chemical structures of R groups are shown in Table 6.

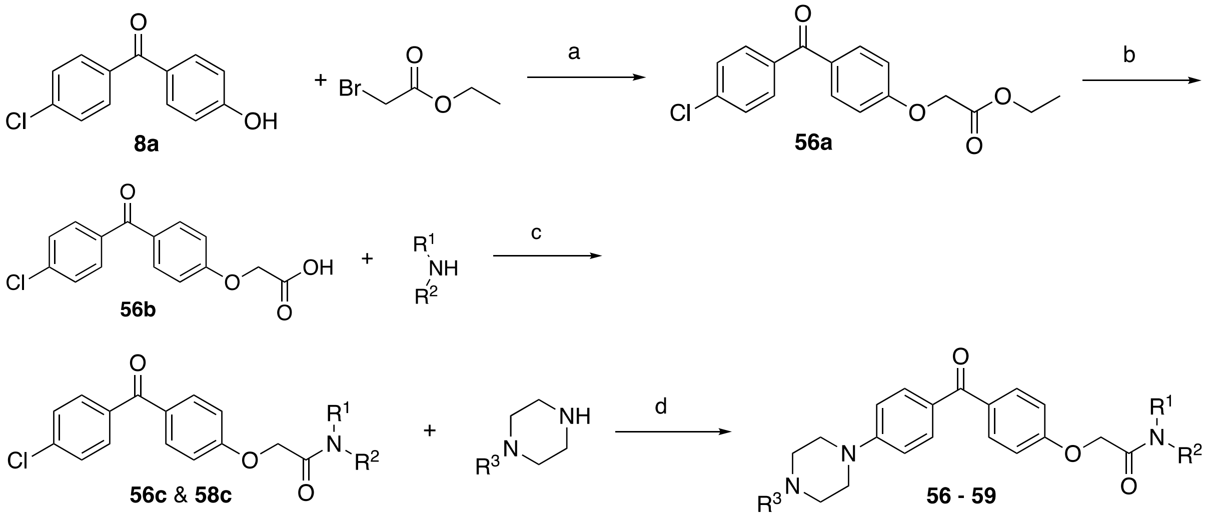

Supporting Figure S9. Synthesis of 56–59. SAR series investigating the combination of regions 1, 2 and 3 of fenofibrate (1)[a,b]. Compounds **56–59** were prepared in three steps from commercially-sourced (4-chlorophenyl) (4-hydroxyphenyl)methanone (**8a**). S_N2 addition with ethyl 2-bromoacetate gave **56a**. Base catalysed ester hydrolysis with lithium hydroxide gave the carboxylic acid **56b**. Amide coupling using HATU and the corresponding amine to give the amides **56c** and **58c** (yield 54% and 63% respectively). Introduction of the corresponding substituted piperazine using a palladium catalysed amination gave target compounds **56–59** (yields: 24% – 35%). [a] Reagents and conditions: (a) Cs_2CO_3, DMAc, 80°C, 75%; (b) LiOH, THF: H_2O (10:1), rt, 89%; (c) HATU, DIPEA, DMAc, rt, 54% and 63%; (d) RuPhosPdG3, Cs_2CO_3, ^{t}BuOH, 80°C, 24% - 35%. [b] Chemical structures of R groups are shown in Table 6.

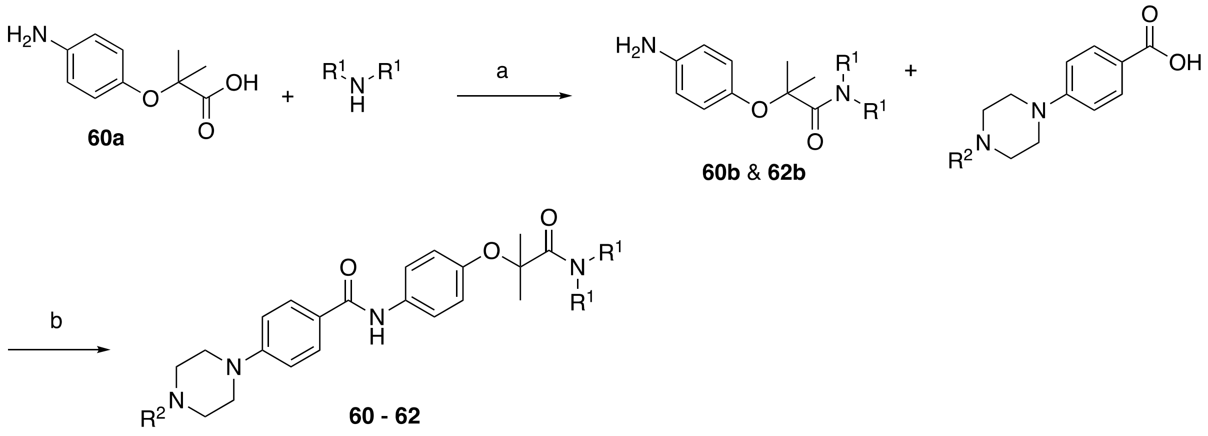

Supporting Figure S10. Synthesis of derivatives 60-62. SAR series investigating combination of regions 1, 3 and 4 of fenofibrate (1): amide analogues. [a,b]. Compounds **60-62** were prepared in two steps from the commercially sourced 2-(4-aminophenoxy)-2-methylpropanoic acid (**60a**). Amide coupling of **60a** using the coupling agent HATU and the corresponding amines gave **60b** and **62b** (yield 55% and 67% respectively). An additional amide coupling of the corresponding 4-(piperazin-1-yl) benzoic acid gave the targeted compounds **60-62** (yields: 34% - 41%). [a] Reagents and conditions: (a) HATU, DIPEA, DMAc, rt, 55 and 67%; (b) HATU, DIPEA, DMAc, rt, 34 - 41%. [b] Chemical structures of R groups are shown in Table 6.

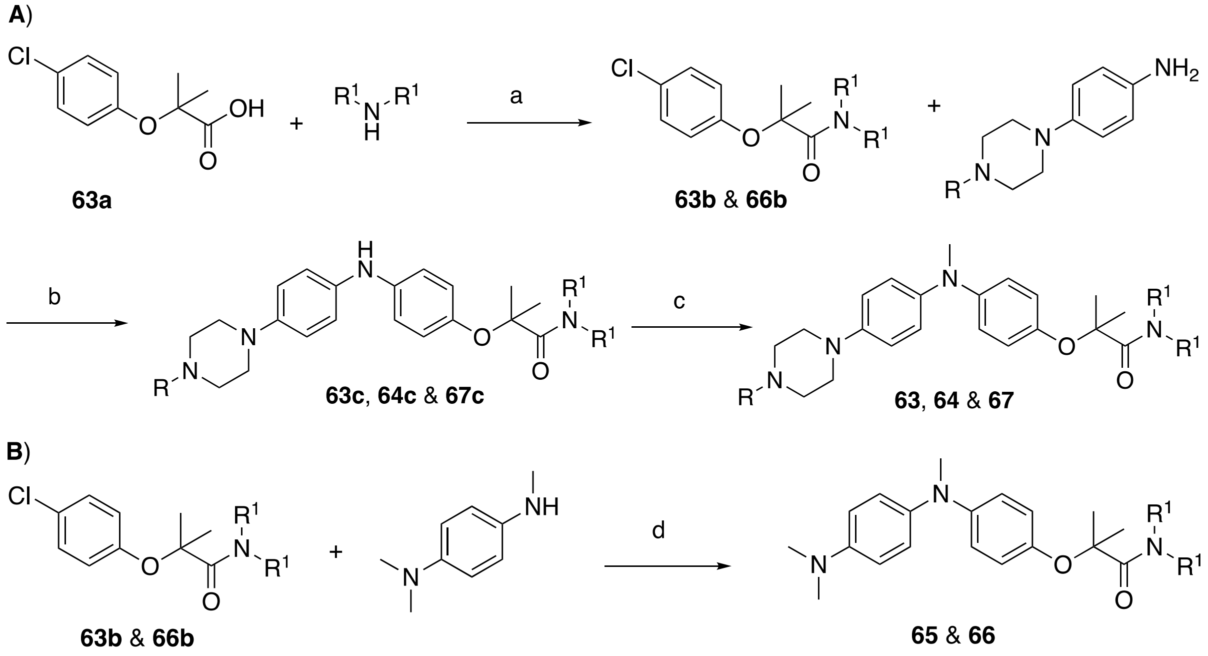

Supporting Figure S11. Synthesis of derivatives 63-67. SAR series investigating the combination of region 1, 3 and 4 of fenofibrate (1)): tertiary amine analogues. [a,b]. **A)** Compounds **63**, **64** and **67** were prepared in three steps from the commercially sourced Clofibric acid **63a**. An amide coupling of **63a** was carried out using HATU and the corresponding amines to give the amides **63b** and **66b**. Followed by a palladium amination with either **63b** or **66b** and the corresponding 4-(piperazin-1-yl) aniline gave the secondary amines intermediates **63c**, **64c** and **67c** (yields: 49% - 64%). Conversion to the tertiary amine was *via* a reductive amination using formaldehyde and sodium triacetoxyborohydride to give **63**, **64** and **67** (yields: 34% – 62%). **B**) Compounds **65** and **66** were prepared via an additional step using commercially available N^1, N^1, N^4-trimethylbenzene-1,4-diamine, which was coupled with either **63b** or **66b** *via* palladium catalysed amination to give target compounds (yields 37 % and 56% respectively).

[a] Reagents and conditions: (a) HATU, DIPEA, DMAc, rt, 84% and 68%; (b) BrettPhosPdG3, Cs_2CO_3, ^{t}BuOH, 80°C, 49% - 64%; (c) formaldehyde, $NaBH(OAc)_3$, CH_2Cl_2, 50°C, 16hr, 34% - 62%; (d) RuPhosPdG3, Cs_2CO_3, ^{t}BuOH, 80°C, 37% and 56%. [b] Chemical structures of R groups are shown in Table 6.

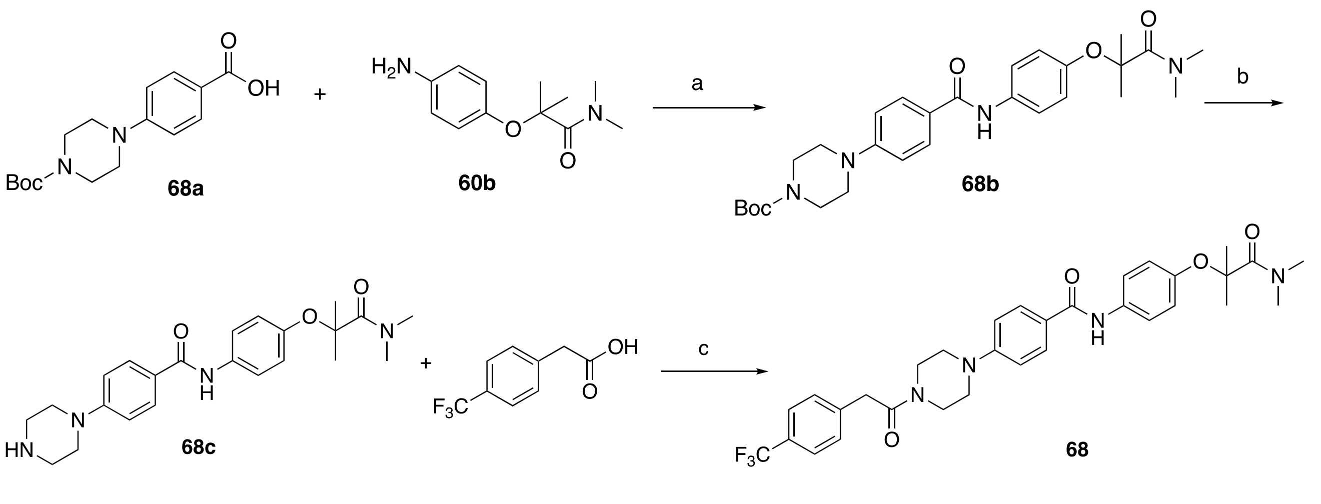

Supporting Figure S12. Synthesis of 68[a]. Compound **68** were prepared in three steps from the commercially sourced 4-(4-(*tert*-butoxycarbonyl)piperazin-1-yl)benzoic acid **68a**. Amide coupling with **60b** using HATU gave **68b**. Acid catalysed Boc deprotection gave **68c**. Amide coupling with 2-(4-(trifluoromethyl)phenyl)acetic acid using HATU gave the target compound **68** (yield 35%). [a] Reagents and conditions: (a) HATU, DIPEA, DMAc, rt, 45%; (b) trifluoroacetic acid, CH_2Cl_2, 3hr, rt, 94%; (c) HATU, DIPEA, DMF, rt, 35%.

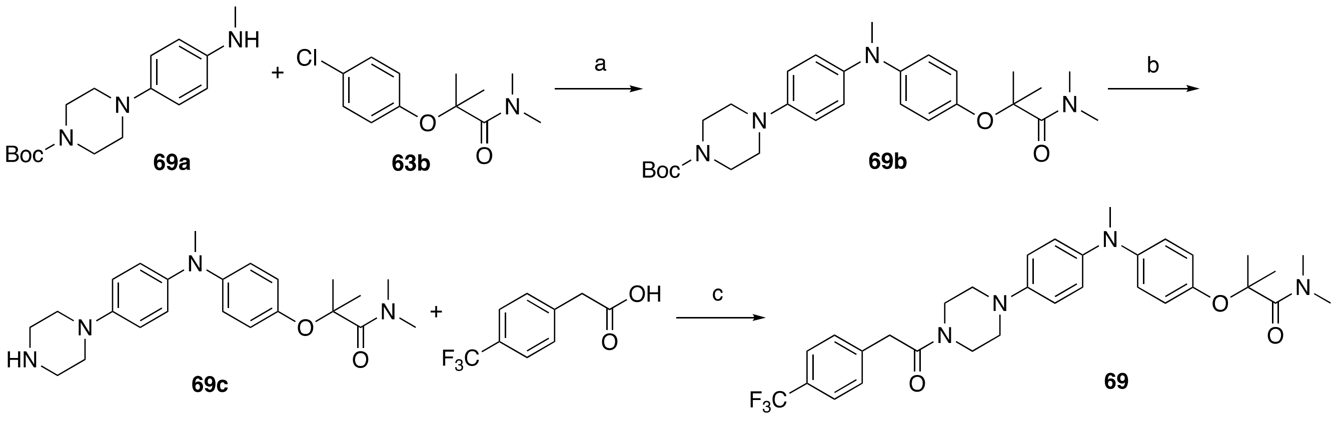

Supporting Figure S13. Synthesis of derivative 69[a]. Compound **69** were prepared in three steps from the commercially sourced *tert*-butyl 4-(4-(methylamino)phenyl)piperazine-1-carboxylate **69a** (yield 64%). Palladium coupling amination with **63b** gave **69b** (yield 86%). Acid catalysed Boc deprotection gave **69c**. Amide coupling with 2-(4-(trifluoromethyl)phenyl)acetic acid using HATU gave the target compound **69** (yield 32%). [a] Reagents and conditions: (a) RuPhos Pd G3, Cs_2CO_3, ^{t}BuOH, 80°C, 64%; (b) trifluoroacetic acid, CH_2Cl_2, 3hr, rt, 86%; (c) HATU, DIPEA, DMF, rt, 32%.

General Synthesis Information

All reagents were purchased from commercial sources (Sigma-Aldrich, Inc.; Fluorochem Ltd; Tokyo Chemical Industries) and were used as received. Anhydrous solvents (Sigma-Aldrich, Inc.) were kept under an atmosphere of nitrogen. Purifications were performed using a Biotage Isolera One purification machine or a Biotage Selekt purification machine (wavelengths monitored: 254 and 280 nm) equipped with pre-packed Biotage® Sfär Duo flash chromatography cartridges. The cartridge type and size as well as solvent gradients (in column volumes, CV) used, are specified in the individual experimental procedures. HPLC grade solvents (Sigma-Aldrich Inc.) were used for purifications, reaction workups, and extractions.

Thin layer chromatography (TLC) was carried out using Merck silica gel 60 F254 TLC plates and visualized using UV light. Melting points (m.p.) were determined using a Stuart SMP-40 automated melting point apparatus. Infrared (IR) spectroscopy was performed using a Bruker Tensor-27 Fourier transform infrared (FT-IR) spectrometer. High-resolution mass spectrometry (HRMS) was performed using electrospray ionization (ESI) mass spectrometry (MS) in the positive or negative ionization mode employing a Thermo Scientific Exactive mass spectrometer (ThermoFisher Scientific); data are presented as a mass-to-charge ratio (*m/z*).

Nuclear magnetic resonance (NMR) spectroscopy was performed using a Bruker AVANCE AVIIIHD 600 machine equipped with a 5 mm BB-F/1H Prodigy N_2 cryoprobe. Chemical shifts for 1H NMR are reported in parts per million (ppm) downfield from tetramethylsilane and are referenced to the residual protium in the NMR solvent ($CDCl_3$: δ = 7.26 ppm; DMSO-*d*6: δ = 2.50 ppm). For ^{13}C NMR, chemical shifts are reported in the scale relative to the NMR solvent (CDCl3: δ = 77.2 ppm; DMSO-d^6: δ = 39.5 ppm). For ^{19}F NMR, chemical shifts are reported relative to $CFCl_3$. NMR data are reported as follows: chemical shift, multiplicity (s: singlet, d:

doublet, dd: doublet of doublets, t: triplet, q: quartet, m: multiplet, br: broad signal), coupling constant (*J*, Hz; accurate to 0.5 Hz), and integration. All compounds are >95% pure by NMR.

General Synthetic Procedures

General Procedure A – Amide Coupling

The carboxylic acid (1 equiv.) and *N,N*-diisopropylethylamine (DIPEA) (2.5 equiv.) were dissolved in *N,N*-dimethylacetamide (DMAc) (0.1 M), followed by the addition of propylphosphonic anhydride solution (T3P, 2 equiv., unless another amide coupling agent is stated). The resultant reaction mixture was stirred at room temperature for 30 mins, when the amine (1.2 equiv) was added. The resultant mixture was stirred overnight at room temperature. Water (200 mL) was then added, and the mixture was extracted with ethyl acetate (EtOAc) (3 x 30 mL). The organic fractions were combined, washed with brine, and dried with anhydrous sodium sulfate (Na_2SO_4). The crude compound was then purified using flash column chromatography using over 20 column volumes to give the desired compound .

General Procedure B – Pd-catalysed Amination

The aryl halide (1 equiv), amine (1.2 equiv), caesium carbonate (Cs_2CO_3, 2 equiv), Pd-ligand conjugate (0.1 equiv) were added to a dried microwave vial. The resultant mixture was flushed with N_2 which was removed under vacuum; the procedure was repeated 3 times. The reaction mixture was dissolved in an anhydrous solvent (0.1 M) and heated 16 h at 80°C in a sandbath. The reaction mixture was allowed to cool to room temperature. Water (200 mL) was then added and the mixture was extracted with EtOAc (3 x 30 mL). The organic fractions were combined then washed with brine and dried with anhydrous Na_2SO_4. The crude mixture was then purified using flash column chromatography to give the desired compound.

Synthetic Procedures and Compound Characterisations

***Tert*-Butyl 2-(4-(4-chlorobenzoyl)phenoxy)-2-methylpropanoate – (4)**

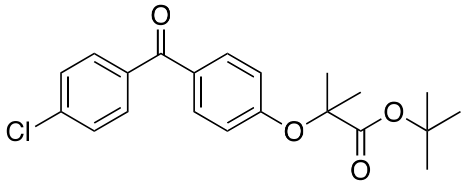

Fenofibric acid (**2**) (100 mg, 0.314 mmol), di-tert-butyl dicarbonate (101 mg, 0.47 mmol) and 4-dimethylaminopyridine (DMAP) (57 mg, 0.47 mmol) were dissolved in anhydrous tert-butanol (3 mL) under N_2. The resultant mixture was stirred at room temperature overnight. Water (30 mL) was then added, and the mixture was extracted with EtOAc (3 x 30 mL). The organic fractions were combined, washed with brine, dried (Na_2SO_4) and the solvent was removed in *vacuo*. The crude mixture was then purified using flash column chromatography using (EtOAc 0 % - 50%, in cyclohexane) over 15 column volumes to give **4** (84 mg, 0.22 mmol, 48%).

^{1}H NMR (400 MHz, DMSO) δ 7.78 – 7.68 (m, 4H), 7.64 – 7.59 (m, 2H), 6.94 – 6.87 (m, 2H), 1.58 (s, 6H), 1.38 (s, 9H). **^{13}C NMR** (101 MHz, DMSO) δ 193.22, 171.66, 159.39, 137.00, 136.31, 131.83, 131.14, 129.36, 128.59, 117.01, 81.71, 79.42, 27.31 (3C, $C(CH_3)_3$), 25.06 (2C, $C(CH_3)_2$). **IR** (film): $\tilde{v}$ = 1723, 1700 cm^{-1}. **HRMS** (ESI-TOF) calc'd for $C_{21}H_{24}O_4{}^{35}Cl$ $[M+H]^+$: 375.1358, found: 375.1364.

Cyclohexyl 2-(4-(4-chlorobenzoyl)phenoxy)-2-methylpropanoate – (5)

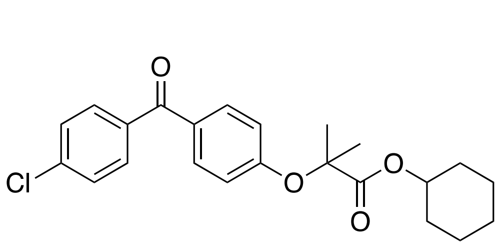

General Procedure A using fenofibric acid (**2**) (100 mg, 0.314 mmol), (1-cyano-2-ethoxy-2-oxoethylidenaminooxy)dimethylamino-morpholino-carbenium hexafluorophosphate (COMU) (399 mg, 0.943 mmol), *N*, *N*-diisopropylethylamine (DIPEA) (116 mg, 0.943 mmol) and cyclohexanol (94 mg, 0.943 mmol) gave **5** (79 mg, 0.197 mmol, 63 %) as a colourless oil.

m.p. 85-92°C. **^{1}H NMR** (400 MHz, DMSO-d_6) δ 7.74 – 7.53 (m, 6H), 6.86 (d, J = 8.5 Hz, 2H), 4.48 (tq, J = 7.0, 3.5 Hz, 1H), 2.05 – 1.41 (m, 16H). **^{13}C NMR** (101 MHz, DMSO-d_6) δ 193.21, 171.95, 159.28, 157.67, 137.04, 136.25, 131.85, 131.15, 129.49, 128.59, 117.05, 79.13, 73.07, 39.52, 30.60 (2C, $(CH_2)_2$), 25.10 (2C, $C(CH_3)_2$), 24.59, 22.81 (2C, $(CH_2)_2$). **IR** (film): ṽ = 1733, 1656, 1597 cm^{-1}. **HRMS** (ESI-TOF) calc'd for $C_{25}H_{26}O_4{}^{35}Cl$ $[M+H]^+$: 401.1514, found : 401.1510.

Phenyl 2-(4-(4-chlorobenzoyl)phenoxy)-2-methylpropanoate – (6)

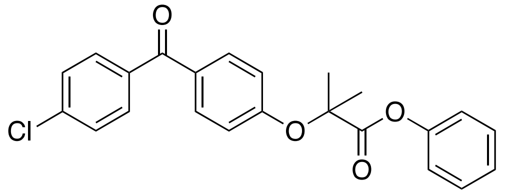

General Procedure A using fenofibric acid (**2**) (100 mg, 0.314 mmol), COMU (399 mg, 0.943 mmol), DIPEA (116 mg, 0.943 mmol) and phenol (90 mg, 0.943 mmol) gave **6** (48 mg, 0.121 mmol, 39 %) as a colourless oil.

^{1}H NMR (400 MHz, DMSO-d_6) δ 7.86 – 7.55 (m, 6H), 7.50 – 7.23 (m, 3H), 7.15 – 7.03 (m, 4H), 1.79 (s, 6H). **^{13}C NMR** (101 MHz, DMSO-d_6) δ 193.25, 171.70, 158.99, 150.07, 137.08, 136.22, 131.19, 129.67, 128.59, 126.34, 121.40, 117.28, 79.09, 25.09 (2C, $C(CH_3)_2$). **IR** (film): ṽ = 1728, 1651, 1595 cm^{-1}. **HRMS** (ESI-TOF) calc'd for $C_{23}H_{20}O_4{}^{35}Cl$ $[M+H]^+$: 395.1044, found: 395.1042.

Benzyl 2-(4-(4-chlorobenzoyl)phenoxy)-2-methylpropanoate – (7)

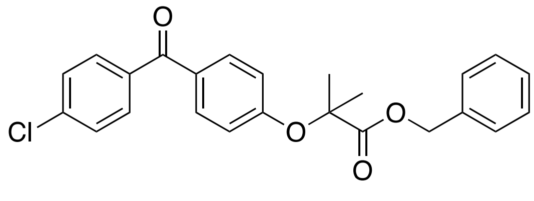

General Procedure A using fenofibric acid (**2**) (100 mg, 0.314 mmol), COMU (399 mg, 0.943 mmol), DIPEA (116 mg, 0.943 mmol) and benzyl alcohol (101 mg, 0.943 mmol) gave **7** (39 mg, 0.0955 mmol, 31%) as a colourless oil.

^{1}H NMR (400 MHz, Chloroform-*d*) δ 7.74 – 7.48 (m, 4H), 7.48 – 7.13 (m, 8H), 6.87 – 6.65 (m, 2H), 5.16 (s, 2H), 1.64 (s, 6H). **^{13}C NMR** (101 MHz, Chloroform-*d*) δ 194.38, 173.70, 159.81, 138.62, 136.66, 135.40, 132.27, 131.48, 130.58, 129.47, 129.20, 129.08, 128.84, 128.83, 128.79, 128.76, 117.54, 79.72, 67.63, 25.73 (2C, $C(CH_3)_2$). **IR** (film): ṽ = 1730, 1655, 1593 cm^{-1}. **HRMS** (ESI-TOF) calc'd for $C_{24}H_{21}O_4{}^{35}Cl^{23}Na$ $[M+H]^+$: 431.1015, found: 431.1015.

3-(4-(4-Chlorobenzoyl)phenoxy)-3-methylbutan-2-one – (8)

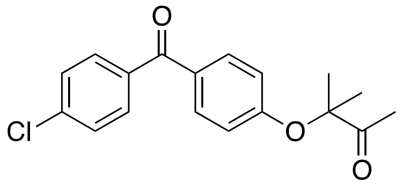

A solution of 4-chlorophenone (426 mg, 1.84 mmol), caesium carbonate (Cs_2CO_3) (994 mg, 3.06 mmol) and *N,N*-dimethylacetamide (DMAc) (10 mL) was prepared; 3-bromo-3-methyl-2-butanone (230 mg, 1.53 mmol) was then added. The resultant mixture was stirred at room temperature overnight. Water (30 mL) was then added, and the mixture was extracted with EtOAc (3 x 30 mL). The organic fractions were combined, washed with brine, dried (Na_2SO_4) and the solvent was removed in *vacuo*. The crude mixture was then purified using flash column chromatography using (EtOAc 0 %- 50%, in cyclohexane) over 15 column volumes to give **8** (308 mg, 0.97 mmol, 53%).

^{1}H NMR (400 MHz, DMSO) δ 7.78 – 7.67 (m, 4H), 7.64 – 7.58 (m, 2H), 6.92 – 6.84 (m, 2H), 2.24 (s, 3H), 1.52 (s, 6H). **^{13}C NMR** (101 MHz, DMSO) δ 209.66, 193.21, 159.15, 137.11, 136.18, 132.07, 131.23, 129.74, 128.62, 117.38, 84.42, 24.50 (2C, $C(CH_3)_2$), 23.66. **IR** (film): ṽ = 1774, 1691 cm^{-1}. **HRMS** (ESI-TOF) calc'd for $C_{18}H_{18}O_3{}^{35}Cl$ $[M+H]^+$: 317.0939, found: 317.0941.

2-(4-(4-Chlorobenzoyl)phenoxy)-*N*-isopropyl-2-methylpropanamide – (9)

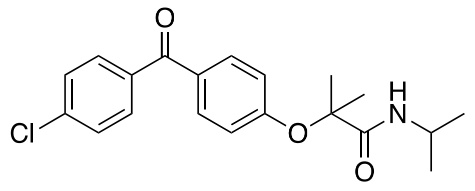

General Procedure A using fenofibric acid (**2**) (80 mg, 0.25 mmol), 1-[bis(dimethylamino)methylene]-1H-1,2,3-triazolo[4,5-b]pyridinium 3-oxide hexafluorophosphate (HATU) (190 mg, 0.50 mmol), DIPEA (258 µL, 1.50 mmol) and isopropylamine (32 µL, 0.375 mmol) gave **9** (55 mg, 0.155 mmol, 62%) as a colourless oil.

^{1}H NMR (400 MHz, DMSO) δ 7.84 (d, *J* = 8.0 Hz, 1H), 7.75 – 7.67 (m, 4H), 7.64 – 7.58 (m, 2H), 6.99 – 6.92 (m, 2H), 3.93 (dhept, *J* = 8.0, 6.5 Hz, 1H), 1.51 (s, 6H), 1.00 (d, *J* = 6.5 Hz, 6H). **^{13}C NMR** (101 MHz, DMSO) δ 193.25, 171.49, 159.23, 137.00, 136.25, 131.55, 131.11, 129.65, 128.56, 118.23, 80.62, 24.97 (2C, $C(CH_3)_2$), 21.86 (2C, $NH(CH_3)_2$). **IR** (film): ṽ = 1751, 1696 cm^{-1}. **HRMS** (ESI-TOF) calc'd for $C_{20}H_{23}O_3{}^{35}Cl$ $[M+H]^+$: 360.1361, found: 360.1363.

2-(4-(4-Chlorobenzoyl)phenoxy)-N-ethyl-2-methylpropanamide – (10)

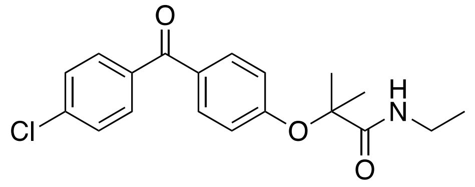

General Procedure A using fenofibric acid (**2**) (250 mg, 0.78 mmol), HATU) 745 mg, 1.96 mmol), DIPEA (386 µL, 2.34 mmol) and Ethylamine (2M in THF (780 µL, 1.56 mmol)) gave **10** (219 mg, 0.63 mmol, 81%) as a white solid.

m.p. 137-139°C. **^{1}H NMR** (400 MHz, DMSO-d_6) δ 8.16 (t, J = 6.0 Hz, 1H), 7.76 – 7.56 (m, 6H), 7.01 – 6.90 (m, 2H), 3.12 (qd, J = 7.0, 6.0 Hz, 2H,), 1.51 (s, 6H), 0.95 (t, J = 7.0 Hz, 3H). **^{13}C NMR** (101 MHz, DMSO-d_6) δ 193.24, 172.28, 159.21, 137.06, 136.22, 131.63, 131.16, 129.69, 128.58, 118.24, 80.65, 33.65, 25.01 (2C, $C(CH_3)_2$), 14.49. **IR** (film): ṽ = 1679, 1649 cm^{-1}. **HRMS** (ESI-TOF) calc'd for $C_{19}H_{21}O_3N^{35}Cl$ $[M+H]^+$: 346.1207, found : 346.1207.

2-(4-(4-Chlorobenzoyl)phenoxy)-*N*-cyclopropyl-2-methylpropanamide – (11)

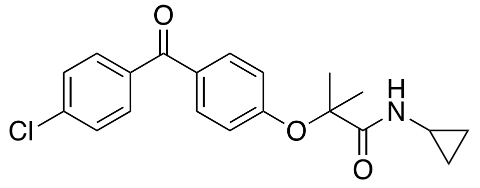

General Procedure A using fenofibric acid (**2**) (80 mg, 0.25 mmol), HATU (190 mg, 0.50 mmol), DIPEA (258 µL, 1.50 mmol) and cyclopropylamine (26 µL, 0.375 mmol) gave **11** (38 mg, 0.107 mmol, 43%) as a colourless oil.

^{1}H NMR (400 MHz, DMSO) δ 8.13 (d, *J* = 4.0 Hz, 1H), 7.75 – 7.68 (m, 4H), 7.64 – 7.58 (m, 2H), 6.97 – 6.89 (m, 2H), 2.64 (ddt, *J* = 11.0, 7.0, 4.0 Hz, 1H), 1.50 (s, 6H), 0.62 – 0.54 (m, 2H), 0.44 – 0.35 (m, 2H). **^{13}C NMR** (101 MHz, DMSO) δ 193.25, 173.90, 159.22, 137.01, 136.24, 131.61, 131.13, 129.66, 128.57, 118.08, 80.53, 24.91(2C, $C(CH_3)_2$), 22.62, 5.42 (2C, $(CH_2)_2$). **HRMS** (ESI-TOF) calc'd for $C_{20}H_{21}O_3N^{35}Cl$ $[M+H]^+$: 358.1204, found: 358.1206.

2-(4-(4-Chlorobenzoyl)phenoxy)-N-cyclohexyl-2-methylpropanamide – (12)

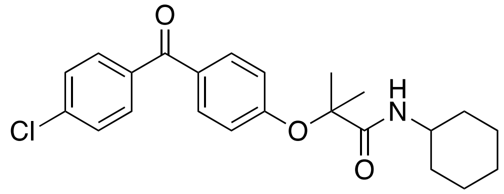

General Procedure A using fenofibric acid (**2**) (250 mg, 0.78 mmol), T3P (623 mg, 1.96 mmol), DIPEA (386 µL, 2.34 mmol) and cyclohexylamine (178 µL, 1.56 mmol) gave **12** (278 mg, 0.69 mmol, 89%) as a white solid.

m.p. 137-139°C· **^{1}H NMR** (400 MHz, DMSO-d_6) δ 7.85 (d, J = 8.0 Hz, 1H), 7.74 – 7.52 (m, 6H), 7.00 – 6.90 (m, 2H), 3.65 – 3.53 (m, 1H), 1.67 – 1.58 (m, 4H), 1.51 (s, 6H), 1.29 – 0.95 (m, 6H). **^{13}C NMR** (101 MHz, DMSO-d_6) δ 193.29, 171.49, 159.26, 137.05, 136.27, 131.63, 131.19, 129.59, 128.62, 118.15, 80.62, 47.96, 31.96 (2C, $(CH_2)_2$), 25.06 (2C, $C(CH_3)_2$), 24.87 (2C, $(CH_2)_2$). **IR** (film): ṽ = 3643, 1720 ,1650, 1596 cm^{-1}. **HRMS** (ESI-TOF) calc'd for $C_{23}H_{27}O_3N^{35}Cl$ $[M+H]^+$: 400.1674, found : 400.1674

_N_-(_tert_-Butyl)-2-(4-(4-chlorobenzoyl)phenoxy)-2-methylpropanamide – (13)

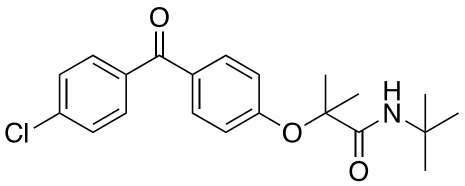

General Procedure A using fenofibric acid (**2**) (80 mg, 0.25 mmol), HATU (190 mg, 0.50 mmol), DIPEA (258 µL, 1.50 mmol) and tert-butylamine (39 µL, 0.375 mmol) gave **13** (31 mg, 0.0825 mmol, 33%) as a colourless oil.

^{1}H NMR (400 MHz, DMSO) δ 7.75 – 7.67 (m, 4H), 7.63 – 7.57 (m, 2H), 7.19 (s, 1H), 7.02 – 6.95 (m, 2H), 1.51 (s, 6H), 1.24 (s, 9H). **^{13}C NMR** (101 MHz, DMSO) δ 193.25, 171.97, 159.12, 137.01, 136.24, 131.57, 131.12, 129.75, 128.56, 118.27, 81.09, 50.43, 28.16 (3C, $C(CH_3)_3$), 24.86 (2C, $C(CH_3)_2$). **IR** (film): ṽ = 1756, 1694 cm^{-1}. **HRMS** (ESI-TOF) calc'd for $C_{21}H_{25}O_3{}^{35}Cl$ [M+H]$^+$: 374.1517, found: 374.1519.

2-(4-(4-Chlorobenzoyl)phenoxy)-_N_,_N_,2-trimethylpropanamide – (14)

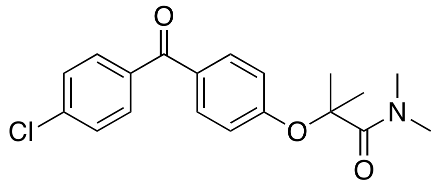

General Procedure A using fenofibric acid (**2**) (80 mg, 0.25 mmol), HATU (190 mg, 0.50 mmol), DIPEA (258 µL, 1.50 mmol) and dimethylamine (25 µL, 0.375 mmol) gave **14** (58 mg, 0.16 mmol, 64%) as a colourless oil.

^{1}H NMR (400 MHz, DMSO) δ 7.77 – 7.68 (m, 4H), 7.64 – 7.57 (m, 2H), 6.95 – 6.87 (m, 2H), 3.04 (s, 3H), 2.84 (s, 3H), 1.61 (s, 6H). **^{13}C NMR** (101 MHz, DMSO) δ 193.14, 171.00, 159.14, 137.01, 136.19, 132.09, 131.15, 129.61, 128.56, 116.30, 81.11, 36.77 (2C, $N(CH_3)_2$), 25.45 (2C, $C(CH_3)_2$). **IR** (film): ṽ = 1698, 1688 cm^{-1} **HRMS** (ESI-TOF) calc'd for $C_{19}H_{21}O_3N^{35}Cl$ [M+H]$^+$: 346.1204, found: 346.1204.

2-(4-(4-Chlorobenzoyl)phenoxy)-*N*,*N*-diethyl-2-methylpropanamide – (15)

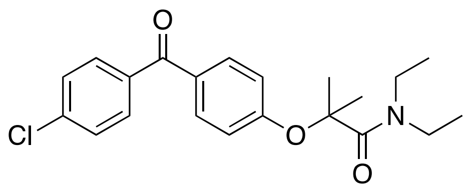

General Procedure A using fenofibric acid (**2**) (80 mg, 0.25 mmol), HATU (190 mg, 0.50 mmol), DIPEA (258 µL, 1.50 mmol) and diethylamine (39 µL, 0.375 mmol) gave **15** (61 mg, 0.162 mmol, 65%) as a colourless oil.

^{1}H NMR (400 MHz, DMSO) δ 7.75 – 7.65 (m, 4H), 7.63 – 7.56 (m, 2H), 6.97 – 6.91 (m, 2H), 3.56 (q, *J* = 7.0 Hz, 2H), 3.27 (q, *J* = 7.0 Hz, 2H), 1.60 (s, 6H), 0.98 (t, *J* = 7.0 Hz, 3H), 0.85 (t, *J* = 7.0 Hz, 3H). **^{13}C NMR** (101 MHz, DMSO) δ 193.14, 170.17, 159.33, 137.02, 136.19, 131.91, 131.12, 129.55, 128.56, 116.65, 81.27, 40.82 (2C, CH_2), 25.86 (2C, $C(CH_3)_2$), 13.53, 12.02. **IR** (film): ṽ = 1698, 1688 cm^{-1}. **HRMS** (ESI-TOF) calc'd for $C_{21}H_{25}O_3N^{35}Cl$ $[M+H]^+$: 374.1517, found: 374.1517.

2-(4-(4-Chlorobenzoyl)phenoxy)-2-methyl-1-(pyrrolidin-1-yl)propan-1-one – (16)

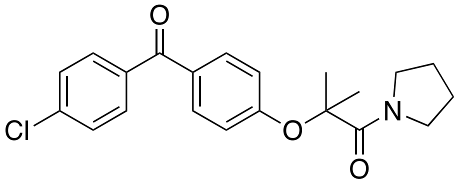

General Procedure A using fenofibric acid (**2**) (80 mg, 0.25 mmol), HATU (190 mg, 0.50 mmol), DIPEA (258 µL, 1.50 mmol) and pyrrolidine (31 µL, 0.375 mmol) gave **16** (52 mg, 0.14 mmol, 56%) as a colourless oil.

^{1}H NMR (400 MHz, DMSO) δ 7.76 – 7.68 (m, 4H), 7.64 – 7.57 (m, 2H), 6.95 – 6.90 (m, 2H), 3.46 – 3.35 (m, 4H), 1.75 – 1.60 (m, 4H), 1.58 (s, 6H). **^{13}C NMR** (101 MHz, DMSO) δ 193.16, 169.97, 159.22, 137.00, 136.21, 132.09, 131.15, 129.60, 128.57, 116.44, 80.86, 47.30, 46.34, 26.39, 24.80 (2C, $(CH_3)_2$), 22.45. **HRMS** (ESI-TOF) calc'd for $C_{21}H_{23}O_3N^{35}Cl$ $[M+H]^+$: 372.1361, found: 372.1363.

Isopropyl 2-(4-(4-chlorobenzoyl)phenoxy)acetate – (17)

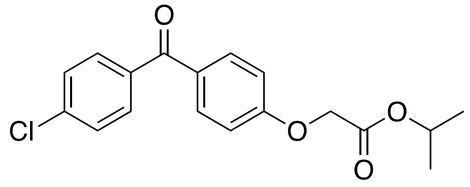

General Procedure A using **56b** (50 mg, 0.172 mmol), COMU (221 mg, 0.517 mmol), DIPEA (88 µL, 0.517 mmol) and isopropanol (395 µL, 5.17 mmol) gave **17** (26 mg, 0.078 mmol, 46%) as a colourless oil.

^{1}H NMR (400 MHz, DMSO-*d*$_6$) δ 7.80 – 7.56 (m, 6H), 7.12 – 7.05 (m, 2H,), 5.01 (hept, J = 6.0 Hz, 1H), 4.88 (s, 2H), 1.22 (d, J = 6.0 Hz, 6H). **^{13}C NMR** (101 MHz, DMSO-*d*$_6$) δ 193.25, 167.75, 161.42, 137.03, 136.30, 132.07, 131.17, 129.64, 128.60, 114.52, 64.85, 62.62, 20.13 (2C, $CH(CH_3)_2$). **IR** (film): ṽ = 1729, 1654, 1597 cm^{-1}. **HRMS** (ESI-TOF) calc'd for $C_{18}H_{18}O_4{}^{35}Cl$ [M+H]$^+$: 333.0888, found : 333.0889.

Ethyl 2-(4-(4-chlorobenzoyl)phenoxy)propanoate – (18)

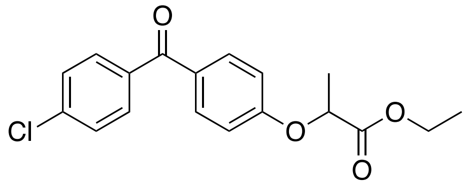

A mixture of (4-chlorophenyl)(4-hydroxy-phenyl)methanone (300 mg, 1.29 mmol), Ethyl 2-bromopropanoate (280 mg, 1.55 mmol), Cs_2CO_3 (504 mg, 1.55 mmol) and *N,N*-dimethylacetamide (10 mL) was prepared. The resultant mixture was heated at 80°C overnight. Water (30 mL) was then added, and the mixture was extracted with EtOAc (3 x 30 mL). The organic fractions were combined, washed with brine, dried (Na_2SO_4) and the solvent was removed in *vacuo*. The crude mixture was then purified using flash column chromatography using (EtOAc 0 %- 25%, in cyclohexane) over 10 column volumes to give **18** (402 mg, 1.21 mmol, 94 %) as a white solid.

m.p. 123-126°C. **^{1}H NMR** (400 MHz, DMSO-*d*$_6$) δ 7.79 – 7.56 (m, 6H), 7.08 – 6.99 (m, 2H), 5.13 (q, J = 6.5 Hz, 1H), 4.18 – 4.13 (q, J = 7.0 Hz, 2H), 1.55 (d, J = 6.5 Hz, 3H), 1.17 (t, J =

7.0 Hz, 3H). **^{13}C NMR** (101 MHz, DMSO-d_6) δ 193.21, 170.92, 161.04, 137.04, 136.27, 132.14, 131.17, 129.58, 128.60, 128.60, 114.75, 71.74, 61.01, 39.52, 18.10, 13.98. **IR** (film): ṽ = 1733, 1653, 1593 cm^{-1}. **HRMS** (ESI-TOF) calc'd for $C_{18}H_{18}O_4{}^{35}Cl$ $[M+H]^+$: 333.0885, found: 333.0885.

Ethyl 2-(4-(4-chlorobenzoyl)phenoxy)butanoate – (19)

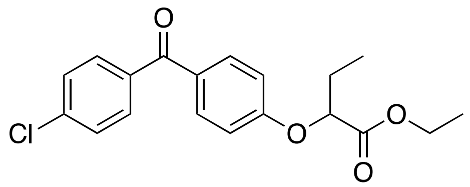

A mixture of (4-chlorophenyl)(4-hydroxy-phenyl) methanone (300 mg, 1.29 mmol), Ethyl 2-bromobutanoate (302 mg, 1.55 mmol), Cs_2CO_3 (504 mg, 1.55 mmol) and *N,N*-dimethylacetamide (10 mL) was prepared. The resultant mixture was heated at 80°C overnight. Water (30 mL) was then, and the mixture was extracted with EtOAc (3 x 30 mL). The organic fractions were combined, washed with brine, dried (Na_2SO_4) and the solvent was removed in *vacuo*. The crude mixture was then purified using flash column chromatography using (EtOAc 0 % - 25%, in cyclohexane) over 10 column volumes to give **19** (375 mg, 1.08 mmol, 84%) as a white solid.

m.p. 114-116°C. **^{1}H NMR** (400 MHz, DMSO-d_6) δ 7.80 – 7.55 (m, 6H, Ar), 7.07 – 6.99 (m, 2H, Ar), 4.96 (t, J = 7.0, 5.0 Hz, 1H), 4.16 (qt, J = 7.0, 3.5 Hz, 2H), 1.95 (dq, J = 14.5, 7.0 Hz, 2H), 1.18 (t, J = 7.0 Hz, 3H), 1.00 (t, J = 7.0 Hz, 3H). **^{13}C NMR** (101 MHz, DMSO-d_6) δ 193.19, 170.27, 161.35, 137.06, 136.27, 132.15, 131.18, 129.60, 128.58, 114.76, 76.42, 60.93, 39.52, 25.41, 14.03, 9.22. **IR** (film): ṽ = 1731, 1656, 1597 cm^{-1}. **HRMS** (ESI-TOF) calc'd for $C_{19}H_{20}O_4{}^{35}Cl$ $[M+H]^+$: 347.1044, found: 347.1043.

Ethyl 2-(4-(4-chlorobenzoyl)phenoxy)-3-phenylpropanoate – (20)

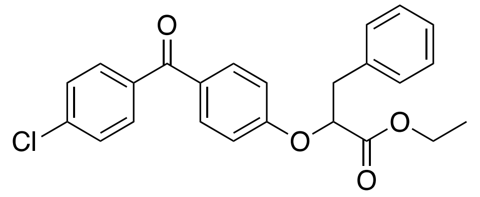

A mixture of (4-chlorophenyl)(4-hydroxyphenyl)methanone (300 mg, 1.29 mmol), ethyl 2-bromo-3-phenylpropanoate (396 mg, 1.55 mmol), Cs_2CO_3 (504 mg, 1.55 mmol) and *N,N*-dimethylacetamide (10 mL) was prepared. The resultant mixture was heated at 80°C overnight. Water (30 mL) was then added, and the mixture was extracted with EtOAc (3 x 30 mL). The organic fractions were combined, washed with brine, dried (Na_2SO_4) and the solvent was removed in *vacuo*. The crude mixture was then purified using flash column chromatography using (EtOAc 0 %- 25%, in cyclohexane) over 15 column volumes gave **20** (82 mg, 0.20 mmol, 16%) with the colourless oil.

^{1}H NMR (400 MHz, DMSO-d_6) δ 7.74 – 7.55 (m, 6H), 7.37 – 7.18 (m, 5H), 7.07 – 6.98 (m, 2H), 5.27 (t, J = 7.0, 5.5 Hz, 1H), 4.10 (d, J = 7.0 Hz, 2H), 3.24 (dd, J = 7.0, 2.0 Hz, 2H), 1.11 (t, J = 7.1 Hz, 3H). **^{13}C NMR** (101 MHz, DMSO-d_6) δ 193.24, 169.78, 161.09, 137.08, 136.03, 132.15, 131.21, 128.62, 128.29, 126.81, 114.83, 76.27, 61.05, 39.52, 37.96, 13.94. **IR** (film): ṽ = 1727, 1652, 1595 cm^{-1}. **HRMS** (ESI-TOF) calc'd for $C_{24}H_{22}O_4{}^{35}Cl$ $[M+H]^+$: 409.1201, found: 409.1202.

Isopropyl 2-(4-benzoylphenoxy)-2-methylpropanoate – (21)

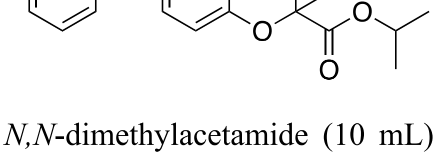

A mixture of (4-hydroxyphenyl)(phenyl)methanone (300 mg, 1.51 mmol), isopropyl 2-bromo-2-methylpropanoate (380 mg, 1.81 mmol), Cs_2CO_3 (588 mg, 1.81 mmol) and *N,N*-dimethylacetamide (10 mL) was prepared. The resultant mixture was heated at 80°C overnight. Water (30 mL) was added, and the mixture was extracted with EtOAc (3 x 30 mL).

The organic fractions were combined, washed with brine, dried (Na_2SO_4) and the solvent was removed in *vacuo*. The crude mixture was then purified using flash column chromatography using (EtOAc 0 %- 25%, in cyclohexane) over 15 column volumes gave **21** (123 mg, 0.377 mmol, 25 %) as a white solid.

m.p. 85-88°C. **^{1}H NMR** (400 MHz, DMSO-d_6) δ 7.76 – 7.50 (m, 7H), 6.93 – 6.86 (m, 2H), 4.95 (hept, J = 6.0 Hz, 1H), 1.60 (s, 6H), 1.14 (d, J = 6.0 Hz, 6H). **^{13}C NMR** (101 MHz, DMSO-d_6) δ 194.32, 172.12, 159.07, 137.59, 132.13, 131.82, 129.83, 129.25, 128.44, 117.04, 79.02, 68.90, 25.02 (2C, $C(CH_3)_2$), 21.20 (2C, $CH(CH_3)_2$). **IR** (film): ṽ = 1728, 1672, 1593 cm^{-1}. **HRMS** (ESI-TOF) calc'd for $C_{20}H_{23}O_4$ $[M+H]^+$: 327.1590, found: 327.1588.

Isopropyl 2-(4-(4-(dimethylamino)benzoyl)phenoxy)-2-methylpropanoate – (22)

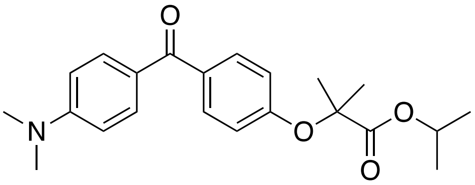

General Procedure B using fenofibrate (**1**) (200 mg, 0.555 mmol), RuPhos Pd G3 (23 mg, 0.0277 mmol), Cs_2CO_3 (541 mg, 1.66 mmol) and dimethylamine (2M in THF (333 µL, 0.666 mmol)) gave **22** (34 mg, 0.092 mmol, 17%) as a white solid.

m.p. 91-93°C. **^{1}H NMR** (400 MHz, DMSO-d_6) δ 7.79 – 7.47 (m, 4H), 7.02 – 6.39 (m, 4H), 4.98 (hept, J = 6.0 Hz, 1H), 3.03 (s, 6H), 1.59 (s, 6H), 1.16 (d, J = 6.0 Hz, 6H). **^{13}C NMR** (101 MHz, DMSO-d_6) δ 192.39, 172.30, 157.92, 153.02, 131.89, 130.98, 123.99, 116.98, 110.70, 78.90, 68.83, 39.52 (2C, $N(CH_3)_2$), 25.07 (2C, $C(CH_3)_2$), 21.24 (2C, $CH(CH_3)_2$). **IR** (film): ṽ = 1732, 1594 cm^{-1}. **HRMS** (ESI-TOF) calc'd for $C_{22}H_{28}O_4N$ $[M+H]^+$: 370.2013, found: 370.2006.

Isopropyl 2-(4-(4-((2-ethoxy-2-oxoethyl)amino)benzoyl)phenoxy)-2-methylpropanoate – (23)

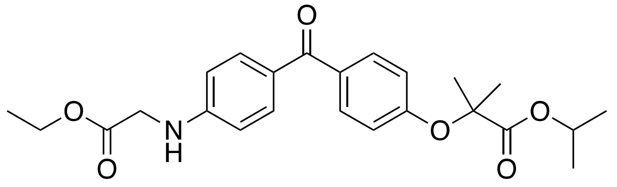

General Procedure B using fenofibrate (**1**) (200 mg, 0.555 mmol), BrettPhos Pd G3 (25 mg, 0.0277 mmol), Cs_2CO_3 (541 mg, 1.66 mmol) and glycine ethyl ester (92 mg, 0.666 mmol) gave **23** (212 mg, 0.49 mmol, 89%) as a clear oil.

^{1}H NMR (400 MHz, Chloroform-*d*) δ 7.73 – 7.62 (m, 4H), 6.86 – 6.81 (m, 2H), 6.61 – 6.55 (m, 2H), 5.07 (hept, J = 6.0 Hz, 1H), 4.25 (q, J = 7.0 Hz, 2H), 3.94 (s, 2H), 1.63 (s, 6H), 1.29 (t, J = 7.0 Hz, 3H), 1.18 (d, J = 6.0 Hz, 6H). **^{13}C NMR** (101 MHz, Chloroform-*d*) δ 193.96, 173.06, 170.11, 158.40, 150.23, 132.39, 131.61, 131.20, 127.08, 118.94, 116.93, 111.36, 78.99, 77.16, 68.99, 61.38, 44.80, 25.11 (2C, $C(CH_3)_2$), 21.26 (2C, $CH(CH_3)_2$), 13.93. **IR** (film): ṽ = 3352, 1731, 1599 cm^{-1}. **HRMS** (ESI-TOF) calc'd for $C_{24}H_{30}O_6N$ [M+H$^+$: 428.2068, found: 428.2066.

Isopropyl 2-(4-(4-(benzylamino)benzoyl)phenoxy)-2-methylpropanoate – (24)

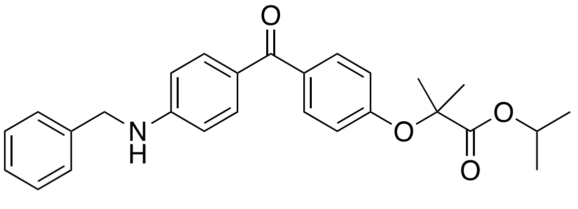

General Procedure B using fenofibrate (**1**) (200 mg, 0.55 mmol), BrettPhos Pd G3 (25 mg, 0.0277 mmol), Cs_2CO_3 (541 mg, 1.66 mmol) and benzylamine (71 mg, 0.66 mmol), *tert* butanol (0.1 M) gave **24** (194 mg, 0.45 mmol, 82%) as a clear oil.

^{1}H NMR (400 MHz, Chloroform-*d*) δ 7.81 – 7.47 (m, 4H), 7.29 – 7.17 (m, 5H), 6.78 – 6.74 (m, 2H), 6.54 – 6.50 (m, 2H), 5.00 (hept, J = 6.0 Hz, 1H), 4.74 (t, J = 5.5 Hz, 1H), 4.31 (d, J =

5.5 Hz, 2H), 1.56 (s, 6H), 1.12 (d, J = 6.0 Hz, 6H). **^{13}C NMR** (101 MHz, Chloroform-*d*) δ 193.89, 173.09, 158.28, 151.42, 138.11, 132.44, 131.80, 131.14, 128.49, 127.05, 116.93, 111.24, 78.97, 68.98, 47.27, 25.11 (2C, $C(CH_3)_2$), 21.27 (2C, $CH(CH_3)_2$). **IR** (film): ṽ = 3339, 1729, 1598 cm^{-1}. **HRMS** (ESI-TOF) calc'd for $C_{27}H_{30}O_4N$ [M+H]$^+$: 432.2169, found: 432.2159.

Isopropyl 2-methyl-2-(4-(4-morpholinobenzoyl)phenoxy)propanoate – (25)

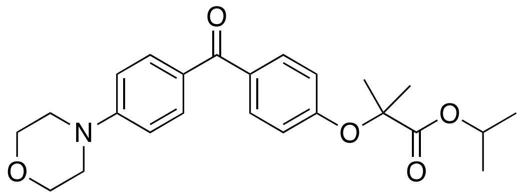

General Procedure B using fenofibrate (**1**) (100 mg, 0.277 mmol), RuPhosPd G3 (23 mg, 0.0277 mmol), Cs_2CO_3 (225 mg, 0.69 mmol), morpholine (35 µL, 0.415 mmol) and tert-butanol (0.1 M) to give **25** (40 mg, 0.096 mmol, 35%) as a yellow oil.

^{1}H NMR (400 MHz, DMSO) δ 7.67 – 7.56 (m, 4H), 7.06 – 7.00 (m, 2H), 6.91 – 6.82 (m, 2H), 4.98 (hept, *J* = 6.0 Hz, 1H), 3.74 (dd, *J* = 5.0 Hz, 4H), 3.30 (dd, *J* = 5.0, 5.0 Hz, 4H), 1.60 (s, 6H), 1.15 (d, *J* = 6.0 Hz, 6H). **^{13}C NMR** (101 MHz, DMSO) δ 192.65, 172.28, 158.24, 153.71, 131.65, 131.19, 126.71, 117.02, 113.03, 78.95, 68.86, 65.88 (2C, $O(CH_2)_2$), 46.86 (2C, $N(CH_2)_2$), 25.06 (2C, $C(CH_3)_2$), 21.23 (2C, $CH(CH_3)_2$). **HRMS** (ESI-TOF) calc'd for $C_{27}H_{30}O_4N$ [M+H]$^+$: 412.2118, found: 412.2120.

Isopropyl 2-methyl-2-(4-(4-(4-methylpiperazin-1-yl)benzoyl)phenoxy)propanoate – (26)

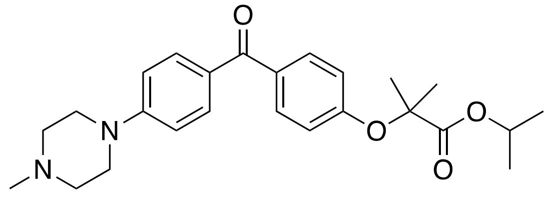

General Procedure B using fenofibrate (**1**) (100 mg, 0.277 mmol), RuPhosPd G3 (23 mg, 0.0277 mmol), Cs_2CO_3 (225 mg, 0.69 mmol), 1-

methylpiperazine (46 µL, 0.415 mmol) and tert-butanol (0.1 M) to give **26** (54 mg, 0.127 mmol, 46%) as a yellow oil.

^{1}H NMR (400 MHz, DMSO) δ 7.67 – 7.58 (m, 4H), 7.02 – 6.98 (m, 2H), 6.89 – 6.83 (m, 2H), 4.98 (hept, *J* = 6.0 Hz, 1H), 3.33 (t, *J* = 5.0 Hz, 4H), 2.43 (d, *J* = 5.0 Hz, 4H), 2.21 (s, 3H), 1.59 (s, 6H), 1.14 (d, *J* = 6.0 Hz, 6H). **^{13}C NMR** (101 MHz, DMSO) δ 192.57, 172.28, 158.17, 153.53, 131.71, 131.31, 131.14, 126.18, 117.01, 113.11, 78.93, 68.85, 54.30 (2C, $NCH_3(CH_2)_2$), 46.51 (2C, $N(CH_2)_2$), 45.71, 25.06 (2C, $C(CH_3)_2$), 21.24 (2C, $CH(CH_3)_2$). **HRMS** (ESI-TOF) calc'd for $C_{25}H_{33}O_4N_2$ $[M+H]^+$: 425.2435, found: 425.2436.

Isopropyl 2-(4-(4-(4-acetylpiperazin-1-yl)benzoyl)phenoxy)-2-methylpropanoate – (27)

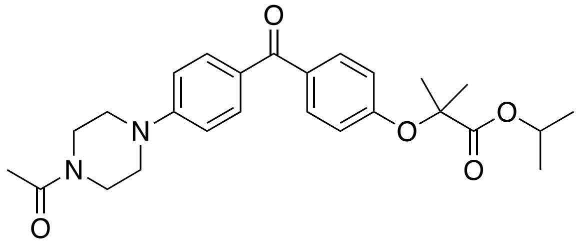

General Procedure A using **29** (50 mg, 0.121 mmol), DIPEA (63 µL, 0.365 mmol), HATU (63 mg, 0.166 mmol), acetic acid (9.5 µL, 0.166 mmol) in N,N-dimethylacetamide (3 mL) gave **27** (37 mg, 0.0823 mmol, 68%) as a yellow oil.

^{1}H NMR (400 MHz, DMSO) δ 7.67 – 7.61 (m, 4H), 7.07 – 7.01 (m, 2H), 6.91 – 6.84 (m, 2H), 4.98 (h, *J* = 6.0 Hz, 1H), 3.67 – 3.57 (m, 4H), 3.44 – 3.33 (m, 4H), 2.06 (s, 3H), 1.60 (s, 6H), 1.16 (d, *J* = 6.0 Hz, 6H). **^{13}C NMR** (101 MHz, DMSO) δ 193.06, 172.75, 168.91, 158.67, 153.66, 132.20, 131.63, 126.89, 117.48, 113.71, 79.41, 69.32, 47.15 (2C, $CON(CH_2)_2$), 45.50 (2C, $N(CH_2)_2$), 25.53 (2C, $C(CH_3)_2$), 21.70 (3C, $CH(CH_3)_2$ & $COCH_3$). **HRMS** (ESI-TOF) calc'd for $C_{26}H_{33}O_5N_2$ $[M+H]^+$: 453.2384, found: 453.2386.

Benzyl 4-(4-(4-((1-isopropoxy-2-methyl-1-oxopropan-2-yl)oxy)benzoyl)phenyl)piperazine-1-carboxylate – (28)

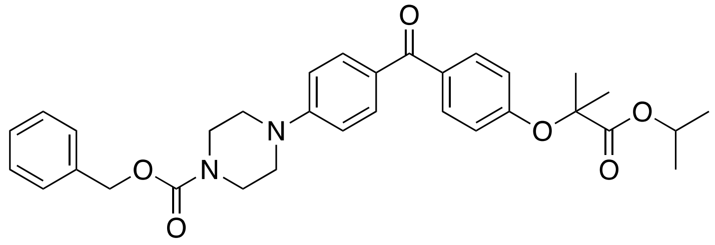

General Procedure B using fenofibrate (**1**) (200 mg, 0.555 mmol), RuPhos Pd G3 (23 mg, 0.0277 mmol), Cs_2CO_3 (541 mg, 1.66 mmol) and Benzyl piperazine-1-carboxylate (145 mg, 0.666 mmol) gave **28** (102 mg, 0.187 mmol, 34%) as a yellow oil.

^{1}H NMR (400 MHz, DMSO-*d*$_6$) δ 7.69 – 7.58 (m, 4H), 7.43 – 7.28 (m, 5H), 7.07 – 6.96 (m, 2H), 6.92 – 6.80 (m, 2H), 5.11 (s, 2H), 4.97 (hept, J = 6.0 Hz, 1H,), 3.62 – 3.51 (m, 4H), 3.40 – 3.37 (m, 4H), 1.59 (s, 6H), 1.15 (d, J = 6.0 Hz, 6H). **^{13}C NMR** (101 MHz, DMSO-*d*$_6$) δ 192.58, 172.27, 158.23, 154.45, 153.20, 136.81, 131.73, 131.19, 128.46, 127.91, 127.65, 116.99, 113.38, 78.93, 68.86, 66.38, 46.43 (2C, $CON(CH_2)_2$), 42.95 (2C, $N(CH_2)_2$), 25.07 (2C, $CH(CH_3)_2$), 21.24 (2C, $CH(CH_3)_2$). **IR** (film): ṽ = 2981, 1703, 1644, 1599 cm^{-1}. **HRMS** (ESI-TOF) calc'd for $C_{32}H_{37}O_6N_2$ $[M-H]^+$: 545.2646, found: 545.2643.

Isopropyl 2-methyl-2-(4-(4-(piperazin-1-yl)benzoyl)phenoxy)propanoate – (29)

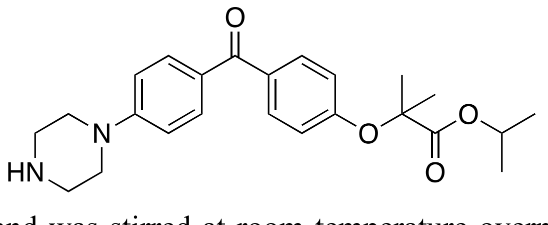

28 (1 g, 1.83 mmol) was dissolved in MeOH (50 mL); 10% Pd/C (193 mg) was then added. The mixture was degassed three times (vacuum/H_2) and was stirred at room temperature overnight. The reaction mixture was filtered through a celite pad and the pad was washed with MeOH (30 mL). The filtrate was concentrated to give **29** (645 mg, 1.57 mmol, 86 %) as a yellow oil.

^{1}H NMR (400 MHz, DMSO) δ 7.67 – 7.58 (m, 4H), 7.08 – 6.98 (m, 2H), 6.91 – 6.84 (m, 2H), 4.98 (h, *J* = 6.0 Hz, 1H), 3.74 (dd, *J* = 5.9, 3.8 Hz, 4H), 3.29 (dd, *J* = 6.0, 3.7 Hz, 4H), 1.59 (s, 6H), 1.15 (d, *J* = 6.3 Hz, 6H). **^{13}C NMR** (101 MHz, DMSO) δ 192.63, 172.26, 158.22, 153.69, 131.62, 131.16, 126.71, 117.02, 113.01, 78.94, 68.84, 65.87 (2C, $N(CH_3)_2$), 46.85 (2C, $NH(CH_3)_2$), 25.05 (2C, $C(CH_3)_2$), 21.22 (2C, $CH(CH_3)_2$). **HRMS** (ESI-TOF) calc'd for $C_{24}H_{31}O_4N_2$ $[M+H]^+$: 411.2278, found: 411.2286.

Isopropyl 2-methyl-2-(4-(4-(4-((3-methylpyridin-2-yl)methyl)piperazin-1-yl)benzoyl)phenoxy)propanoate – (30)

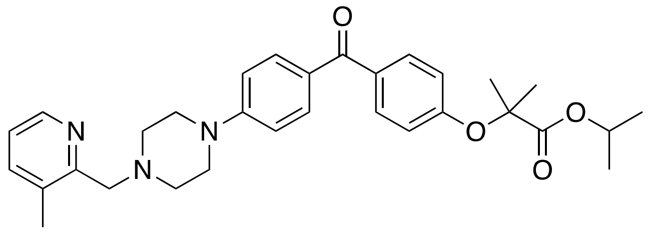

A solution of **29** (70 mg, 0.17 mmol), CH_2Cl_2 (2 mL) and acetic acid (0.01 mL) was prepared; 3-methylpyridine-2-carbaldehyde (19 µL, 0.170 mmol) was then added. The resultant mixture was stirred at room temperature for 10 minutes before the addition of sodium triacetoxyborohydride (104 mg, 0.495 mmol). The reaction mixture was stirred at room temperature for 16 h. A mixture saturated sodium bicarbonate solution and CH_2Cl_2 (20 mL (1:1)) was added. The organic and aqueous phases were separated, and the aqueous phase was extracted with CH_2Cl_2 (2 x 20 mL). The organic fractions were combined, dried (Na_2SO_4) and the solvent was removed in *vacuo*. The crude mixture was then purified using flash column chromatography using (MeOH 0% - 20%, in CH_2Cl_2) over 20 column volumes to give **30** (49 mg, 0.0952 mmol, 56%) as a yellow oil.

^{1}H NMR (400 MHz, DMSO) δ 8.31 (ddd, *J* = 4.8, 1.8, 0.7 Hz, 1H), 7.65 – 7.52 (m, 5H), 7.21 (dd, *J* = 7.6, 4.8 Hz, 1H), 7.01 – 6.95 (m, 2H), 6.89 – 6.83 (m, 2H), 4.98 (p, *J* = 6.3 Hz, 1H), 3.64 (s, 2H), 3.29 (d, *J* = 5.5 Hz, 4H), 2.56 – 2.51 (m, 4H), 2.40 (s, 3H), 1.59 (s, 6H), 1.15 (d, *J* = 6.3 Hz, 6H). **^{13}C NMR** (101 MHz, DMSO) δ 192.47, 172.17, 158.10, 155.91, 153.43,

145.64, 137.77, 132.90, 131.58, 131.36, 130.97, 126.17, 122.46, 117.10, 113.01, 78.94, 68.74, 62.42, 52.49 (2C, $CH_2N(CH_3)_2$), 46.67 (2C, $N(CH_3)_2$), 25.00 (2C, $C(CH_3)_2$), 21.14 (2C, $CH(CH_3)_2$), 17.88. **HRMS** (ESI-TOF) calc'd for $C_{31}H_{38}O_4N_3$ $[M+H]^+$: 516.2857, found: 516.2859.

Isopropyl-2-methyl-2-(4-(4-(4-((1-methyl-1*H*-imidazol-2-yl)methyl)piperazin-1-yl)benzoyl)phenoxy)propanoate – (31)

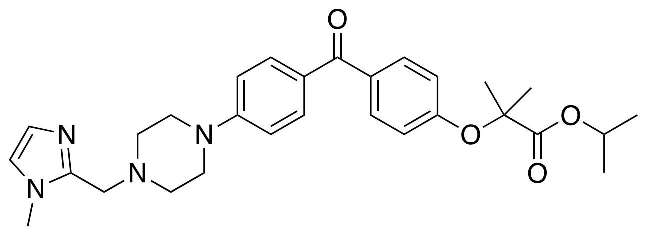

A solution of **29** (70 mg, 0.17 mmol), CH_2Cl_2 (2 mL) and acetic acid (0.01 mL) was prepared; 1-methyl-1H-imidazole-2-carbaldehyde (19 mg, 0.170 mmol) was then added. The resultant mixture was stirred at room temperature for 10 minutes before the addition of sodium triacetoxyborohydride (104 mg, 0.495 mmol). The reaction mixture was stirred at room temperature for 16 h. A mixture saturated sodium bicarbonate solution and CH_2Cl_2 (20 mL (1:1)) was added. The organic and aqueous phases were separated, and the aqueous phase was extracted with CH_2Cl_2 (2 x 20 mL). The organic fractions were combined, dried (Na_2SO_4) and the solvent was removed in *vacuo*. The crude mixture was then purified using flash column chromatography using (MeOH 0% - 20%, in CH_2Cl_2) over 20 column volumes to give **31** (38 mg, 0.0765 mmol, 45%) as a yellow oil.

^{1}H NMR (400 MHz, DMSO) δ 7.66 – 7.54 (m, 4H), 7.09 (d, *J* = 1.2 Hz, 1H), 7.02 – 6.95 (m, 2H), 6.89 – 6.84 (m, 2H), 6.77 (d, *J* = 1.2 Hz, 1H), 4.98 (p, *J* = 6.2 Hz, 1H), 3.67 (s, 3H), 3.57 (s, 2H), 3.34 – 3.27 (m, 4H), 2.56 – 2.43 (m, 4H), 1.59 (s, 6H), 1.15 (d, *J* = 6.2 Hz, 6H). **^{13}C NMR** (101 MHz, DMSO) δ 192.56, 172.26, 158.17, 153.48, 144.04, 131.69, 131.31, 131.12, 126.22, 122.03, 117.03, 113.12, 78.94, 68.84, 53.66, 52.20 (2C, $CH_2N(CH_3)_2$), 46.63 (2C,

N(CH_3)$_2$), 32.52, 25.06 (2C, C(CH_3)$_2$), 21.22 (2C, CH(CH_3)$_2$). **HRMS** (ESI-TOF) calc'd for $C_{29}H_{37}O_4N_4$ [M+H]$^+$: 505.2809, found: 505.2810.

Isopropyl 2-methyl-2-(4-(4-(4-(3-methylpicolinoyl)piperazin-1-yl)benzoyl)phenoxy)-propanoate – (32)

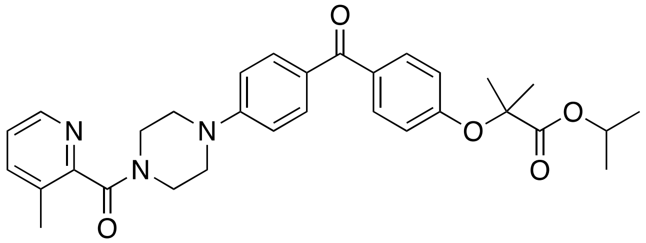

General Procedure A using **29** (50 mg, 0.121 mmol), DIPEA (63 µL, 0.365 mmol), HATU (63 mg, 0.166 mmol), 3-methylpicolinic acid (22 mg, 0.166 mmol) in N,N-dimethylacetamide (0.1 M) gave **32** (24 mg, 0.0447 mmol, 37%) as a yellow oil.

^{1}H NMR (400 MHz, DMSO) δ 8.42 (ddd, *J* = 4.8, 1.6, 0.7 Hz, 1H), 7.76 (ddd, *J* = 7.9, 1.7, 0.8 Hz, 1H), 7.66 – 7.58 (m, 4H), 7.38 (dd, *J* = 7.8, 4.8 Hz, 1H), 7.09 – 7.00 (m, 2H), 6.94 – 6.81 (m, 2H), 4.98 (p, *J* = 6.2 Hz, 1H), 3.86 – 3.75 (m, 2H), 3.52 – 3.46 (m, 2H), 3.26 (dd, *J* = 6.8, 3.6 Hz, 2H), 2.26 (s, 3H), 1.59 (s, 6H), 1.15 (d, *J* = 6.2 Hz, 6H). **^{13}C NMR** (151 MHz, DMSO) δ 192.60, 172.24, 166.57, 158.23, 153.47, 153.13, 146.33, 138.50, 131.67, 131.15, 130.12, 126.72, 123.94, 117.04, 113.52, 78.95, 68.83, 47.09, 46.61, 45.34, 40.47, 25.05 (2C, C(CH_3)$_2$), 21.22 (2C, CH(CH_3)$_2$), 17.13. **HRMS** (ESI-TOF) calc'd for $C_{31}H_{36}O_5N_3$ [M+H]$^+$: 530.2649, found: 530.2650.

Isopropyl 2-methyl-2-(4-(4-(4-(pyrimidine-2-carbonyl)piperazin-1-yl)benzoyl)phenoxy)propanoate – (33)

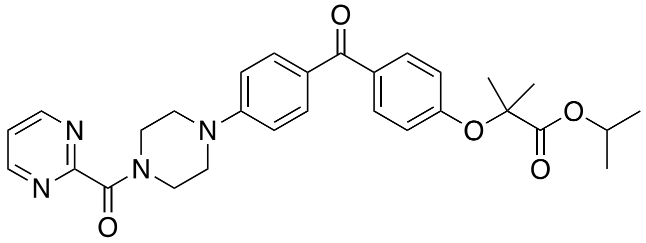

General Procedure A using **29** (50 mg, 0.121 mmol), DIPEA (63 µL, 0.365 mmol), HATU (63 mg, 0.166 mmol), pyrimidine-2-carboxylic acid (21 mg,

0.166 mmol) in N,N-dimethylacetamide (0.1 M) gave **33** (35 mg, 0.0677 mmol, 56%) as a yellow oil.

^{1}H NMR (400 MHz, DMSO) δ 8.93 (d, *J* = 5.0 Hz, 2H), 7.69 – 7.56 (m, 5H), 7.06 – 6.99 (m, 2H), 6.90 – 6.82 (m, 2H), 4.97 (hept, *J* = 6.0 Hz, 1H), 3.84 – 3.77 (m, 2H), 3.54 – 3.47 (m, 2H), 1.59 (s, 7H), 1.15 (d, *J* = 6.0 Hz, 6H). **^{13}C NMR** (101 MHz, DMSO) δ 193.09, 172.74, 165.05, 162.02, 158.71, 158.25, 153.56, 132.18, 131.66, 131.63, 127.21, 122.31, 117.48, 114.00, 79.41, 69.32, 47.43, 46.91, 45.93, 41.16, 25.52 (2C, $C(CH_3)_2$), 21.70 (2C, $CH(CH_3)_2$). **HRMS** (ESI-TOF) calc'd for $C_{29}H_{33}O_5N_4$ $[M+H]^+$: 517.2445, found: 517.2448.

Isopropyl-2-methyl-2-(4-(4-(4-(2-(trifluoromethyl)benzoyl)piperazin-1-yl)benzoyl)phenoxy)propanoate – (34)

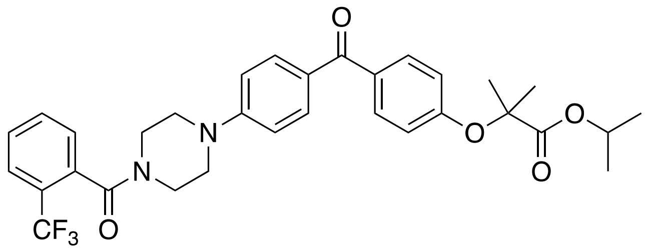

General Procedure A using **29** (50 mg, 0.121 mmol), DIPEA (63 μL, 0.365 mmol), HATU (63 mg, 0.166 mmol), 2-(Trifluoromethyl)benzoic acid (31 mg, 0.166 mmol) in N,N-dimethylacetamide (0.1 M) gave **34** (44 mg, 0.076 mmol, 63%) as a yellow oil.

^{1}H NMR (400 MHz, DMSO) δ 7.86 – 7.82 (m, 1H), 7.80 – 7.74 (m, 1H), 7.69 (dt, *J* = 7.7, 1.2 Hz, 1H), 7.67 – 7.60 (m, 4H), 7.57 – 7.52 (m, 1H), 7.17 – 6.91 (m, 2H), 6.93 – 6.77 (m, 2H), 4.98 (p, *J* = 6.3 Hz, 1H), 3.99 – 3.67 (m, 2H), 3.60 – 3.41 (m, 2H), 1.60 (s, 6H), 1.16 (d, *J* = 6.2 Hz, 6H). **^{13}C NMR** (101 MHz, DMSO) δ 192.48, 172.08, 166.10, 158.14, 152.94, 132.77, 131.49, 131.22, 130.94, 129.46, 127.47, 126.77, 126.45 (q, $^{2}J\_CF$ = 31.8 Hz), 117.13, 113.43, 78.94, 68.68, 46.43, 46.32, 45.86, 40.70, 24.96 (2C, $CH(CH_3)_2$), 21.08 (2C, $CH(CH_3)_2$). **^{19}F NMR** (376 MHz, DMSO) δ -58.51. **HRMS** (ESI-TOF) calc'd for $C_{32}H_{34}O_5N_2F_3$ $[M+H]^+$: 583.2414, found: 583.2442.

Isopropyl 2-methyl-2-(4-(4-(4-(2-(4-(trifluoromethyl)phenyl)acetyl)piperazin-1-yl)benzoyl)phenoxy)propanoate – (35)

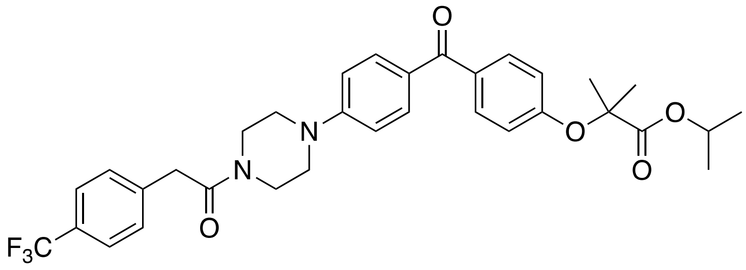

General Procedure A using **29** (50 mg, 0.121 mmol), DIPEA (63 µL, 0.365 mmol), HATU (63 mg, 0.166 mmol), 4-(Trifluoromethyl)phenyl acetic acid (34 mg, 0.166 mmol) in *N,N*-dimethylacetamide (0.1 M) gave **35** (42 mg, 0.0714 mmol, 59%) as a yellow oil.

^{1}H NMR (400 MHz, DMSO) δ 7.74 – 7.59 (m, 6H), 7.48 (d, *J* = 8.0 Hz, 2H), 7.07 – 7.00 (m, 2H), 6.91 – 6.85 (m, 2H), 4.99 (h, *J* = 6.0 Hz, 1H), 3.92 (s, 2H), 3.67 (dt, *J* = 20.3, 5.2 Hz, 4H), 1.60 (s, 6H), 1.15 (d, *J* = 6.0 Hz, 6H). **^{13}C NMR** (101 MHz, DMSO) δ 192.57, 172.24, 168.45, 158.20, 153.09, 131.68, 131.24, 131.12, 130.16, 126.49, 124.96, 117.04, 113.22, 78.94, 68.82, 46.63, 46.31, 44.67, 40.82, 25.04 (2C, $C(CH_3)_2$), 21.20 (2C, $CH(CH_3)_2$). **^{19}F NMR** (376 MHz, DMSO) δ -60.80. **HRMS** (ESI-TOF) calc'd for $C_{33}H_{36}O_5N_2F_3$ $[M+H]^+$: 597.2571, found: 597.2573.

Isopropyl 2-methyl-2-(4-(4-(4-(pyridin-3-ylcarbamoyl)piperazin-1-yl)benzoyl)-phenoxy)propanoate – (36)

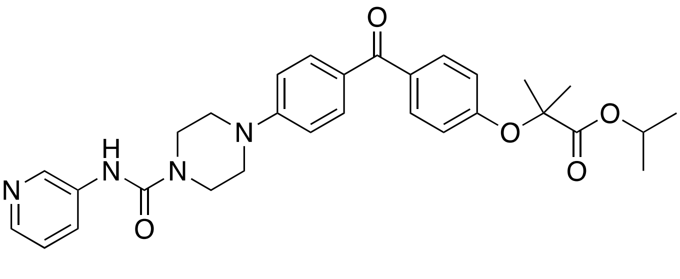

General Procedure A using **29** (50 mg, 0.121 mmol), DIPEA (63 µL, 0.365 mmol), pyridine-3-isocyanate (29 mg, 0.242 mmol) in *N,N*-dimethylacetamide (0.1 M) gave **36** (46 mg, 0.086 mmol, 71%) as a yellow oil.

^{1}**H NMR** (400 MHz, DMSO) δ 8.82 (s, 1H), 8.66 (d, *J* = 2.6 Hz, 1H), 8.16 (dd, *J* = 4.7, 1.5 Hz, 1H), 7.90 (ddd, *J* = 8.4, 2.6, 1.5 Hz, 1H), 7.64 (dd, *J* = 8.2, 1.3 Hz, 3H), 7.28 (ddd, *J* = 8.3, 4.6, 0.7 Hz, 1H), 7.11 – 7.03 (m, 2H), 6.92 – 6.82 (m, 2H), 4.98 (h, *J* = 6.0 Hz, 1H), 3.64 (dd, *J* = 6.8, 3.8 Hz, 4H), 3.47 – 3.41 (m, 4H), 1.60 (s, 6H), 1.15 (d, *J* = 6.0 Hz, 6H). ^{13}**C NMR** (101 MHz, DMSO) δ 192.62, 172.28, 154.79, 153.30, 141.39, 137.14, 131.72, 131.17, 126.50, 123.24, 117.04, 113.28, 78.96, 68.86, 46.45 (2C, CON(CH_3)$_2$), 43.25 (2C, N(CH_3)$_2$), 25.08 (2C, C(CH_3)$_2$), 21.25 (2C, CH(CH_3)$_2$). **HRMS** (ESI-TOF) calc'd for $C_{30}H_{35}O_5N_4$ [M+H]$^+$: 531.2602, found: 531.2612.

Isopropyl 2-(4-(4-chlorobenzamido)phenoxy)-2-methylpropanoate – (39)

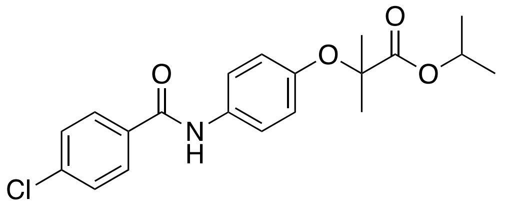

A mixture of 4-Chloro-*N*-(4-hydroxyphenyl)benzamide (300 mg, 1.21 mmol) and Cs_2CO_3 (786 mg, 1.42 mmol) in *N,N*-dimethylacetamide (10 mL) was prepared; isopropyl 2-bromo-2-methylpropanoate (303 mg, 1.45 mmol) was then added. The reaction mixture was heated at 120°C for 16 hr. The reaction mixture was allowed to cool to room temperature. Water (30 mL) was then added, and the mixture was extracted with EtOAc (3 x 30 mL). The organic fractions were combined, washed with brine, dried (Na_2SO_4) and the solvent was removed in *vacuo*. The crude mixture was then purified using flash column chromatography using (EtOAc 0 %- 100%, in cyclohexane) over 10 column volumes gave **39** (254 mg, 0.677 mmol, 56%) as a colourless oil.

^{1}**H NMR** (400 MHz, DMSO) δ 10.24 (s, 1H), 8.01 – 7.90 (m, 2H), 7.69 – 7.54 (m, 4H), 6.84 – 6.79 (m, 2H), 4.98 (h, *J* = 6.0 Hz, 1H), 1.50 (s, 6H), 1.18 (d, *J* = 6.0 Hz, 6H). ^{13}**C NMR** (101 MHz, DMSO) δ 172.70, 164.10, 151.21, 136.29, 133.67, 133.38, 129.55, 128.45, 121.54,

119.15, 78.87, 68.53, 25.01 (2C, $C(CH_3)_2$), 21.32 (2C, $CH(CH_3)_2$). **HRMS** (ESI-TOF) calc'd for $C_{20}H_{23}NO_4{}^{35}Cl$ $[M+H]^+$: 376.1310, found: 376.1318.

Isopropyl 2-(4-chlorophenoxy)-2-methylpropanoate – (40b)

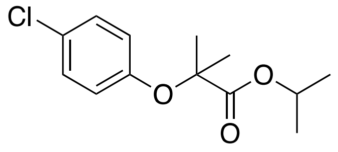

A mixture of 4-chlorophenol (2 g, 0.0156 mol), isopropyl 2-bromo-2-methylpropanoate (3.7 mL, 0.023 mol) and Cs_2CO_3 (10 g, 0.0312 mol) in *N,N*-dimethylacetamide (50 mL) was prepared. The resultant mixture was heated at 80°C overnight. Water (200 mL) was then added, and the mixture was extracted with EtOAc (3 x 50 mL). The organic fractions were combined, washed with brine, dried (Na_2SO_4) and the solvent was removed in *vacuo*. The crude mixture was then purified using flash column chromatography using (EtOAc 0 %- 25%, in cyclohexane) over 10 column volumes to give **40b** (3.03 g, 0.0118 mol, 76 %) as a colourless oil.

^{1}H NMR (400 MHz, $CDCl_3$) δ 7.19 – 7.15 (m, 2H), 6.80 – 6.74 (m, 2H), 5.07 (hept, *J* = 6.0 Hz, 1H), 1.56 (s, 6H), 1.20 (d, *J* = 6.0 Hz, 6H). **^{13}C NMR** (101 MHz, $CDCl_3$) δ 173.47, 154.14, 129.01, 127.00, 120.32, 116.68, 78.83, 69.11, 25.26 (2C, $CH(CH_3)_2$), 21.54 (2C, $CH(CH_3)_2$). **HRMS** (ESI-TOF) calc'd for $C_{13}H_{17}O_3{}^{35}Cl$ $[M+H]^+$: 257.0939, found: 257.0940

Isopropyl 2-(4-((4-(dimethylamino)phenyl)amino)phenoxy)-2-methylpropanoate – (40)

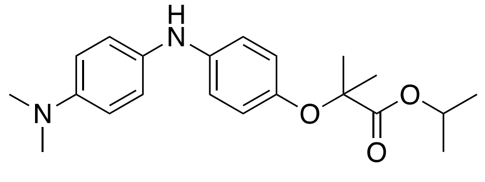

General Procedure B using **40b** (100 mg, 0.39 mmol), BrettPhos Pd G3 (35 mg, 0.039 mmol), Cs_2CO_3 (253 mg, 0.78 mmol), 4-(dimethylamino)aniline (53 mg, 0.39 mmol) and tert-butanol (1.5 mL) gave **40** (90 mg, 0.253 mmol, 65%) as a blue oil.

^{1}H NMR (400 MHz, DMSO) δ 7.46 (s, 1H), 6.95 – 6.89 (m, 2H), 6.79 – 6.73 (m, 2H), 6.72 – 6.66 (m, 4H), 4.94 (h, *J* = 6.0 Hz, 1H), 2.81 (s, 6H), 1.42 (s, 6H), 1.19 (d, *J* = 6.0 Hz, 6H). **^{13}C**

NMR (101 MHz, DMSO) δ 172.86, 146.78, 145.72, 141.25, 133.66, 120.38, 115.20, 114.02, 79.07, 68.28, 40.96 (2C, $N(CH_3)_2$), 24.99 (2C, $C(CH_3)_2$), 21.37 (2C, $CH(CH_3)_2$). **HRMS** (ESI-TOF) calc'd for $C_{21}H_{29}N_2O_3$ $[M+H]^+$: 357.2173, found: 357.2173.

Isopropyl 2-(4-((4-(dimethylamino)phenyl)(methyl)amino)phenoxy)-2-methylpropanoate – (41)

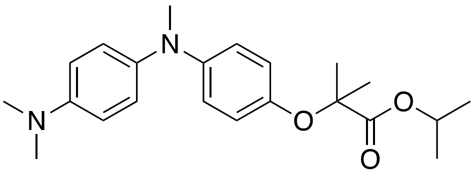

A solution of **40** (50 mg, 0.14 mmol), CH_2Cl_2 (2 mL) and acetic acid (0.01 mL) was prepared; paraformaldehyde (6.3 mg, 0.21 mmol) was then added. The resultant mixture was heated to 50°C for 10 minutes before the addition of sodium triacetoxyborohydride (88 mg, 0.42 mmol). The reaction mixture was stirred at room temperature for 16 h. A mixture saturated $NaHCO_3$ solution and CH_2Cl_2 (20 mL (1:1)) was added. The organic and aqueous phases were separated, and the aqueous phase was then extracted with CH_2Cl_2 (2 x 20 mL). The organic fractions were combined, dried (Na_2SO_4) and the solvent was removed in *vacuo*. The crude mixture was then purified using flash column chromatography using (MeOH 0% - 20%, in CH_2Cl_2) over 20 column volumes to give **41** (39 mg, 0.106 mmol, 76%) as a colourless oil.

^{1}H NMR (400 MHz, DMSO) δ 6.96 – 6.91 (m, 2H), 6.74 – 6.66 (m, 4H), 6.64 – 6.59 (m, 2H), 4.94 (p, *J* = 6.2 Hz, 1H), 3.10 (s, 3H), 2.85 (s, 6H), 1.42 (s, 6H), 1.18 (d, *J* = 6.2 Hz, 6H). **^{13}C NMR** (101 MHz, DMSO) δ 172.81, 147.34, 146.92, 145.48, 138.67, 125.13, 120.85, 116.02, 113.63, 79.02, 68.27, 40.54 (3C, $N(CH_3)_2$ & NCH_3), 24.98 (2C, $C(CH_3)_2$), 21.34 (2C, $CH(CH_3)_2$). **HRMS** (ESI-TOF) calc'd for $C_{22}H_{31}N_2O_3$ $[M+H]^+$: 371.2324, found: 371.2324.

Isopropyl 2-(4-((4-(dimethylamino)phenyl)(ethyl)amino)phenoxy)-2-methylpropanoate – (42)

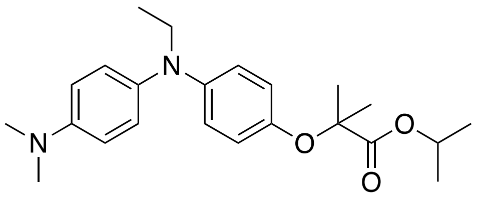

A solution of **40** (50 mg, 0.14 mmol), CH_2Cl_2 (2 mL) and acetic acid (0.01 mL) was prepared; acetaldehyde (9.98 mg, 0.21 mmol) was then added. The resultant mixture was stirred at room temperature for 5 minutes before the addition of sodium triacetoxyborohydride (88 mg, 0.42 mmol). The reaction mixture was stirred at room temperature for 16 h. A mixture saturated $NaHCO_3$ solution and CH_2Cl_2 (20 mL (1:1)) was added. The organic and aqueous phases were separated, and the aqueous phase was extracted with CH_2Cl_2 (2 x 20 mL). The organic fractions were combined, dried (Na_2SO_4) and the solvent was removed in *vacuo*. The crude mixture was then purified using flash column chromatography using (MeOH 0%- 20%, in CH_2Cl_2) over 20 column volumes to give **42** (31 mg, 0.0798 mmol, 57%) as a colourless oil.

^{1}H NMR (400 MHz, DMSO) δ 7.09 – 6.41 (m, 8H), 4.94 (h, *J* = 6.0 Hz, 1H), 3.55 (s, 2H), 2.86 (s, 6H), 1.41 (s, 6H), 1.18 (d, *J* = 6.0 Hz, 6H), 1.06 (t, *J* = 7.0 Hz, 3H). **^{13}C NMR** (101 MHz, DMSO) δ 172.77, 146.47, 144.42, 136.47, 130.33, 126.39, 121.00, 115.74, 115.71, 113.64, 79.01, 68.21, 24.96 (2C, $C(CH_3)_2$), 21.30 (2C, $CH(CH_3)_2$). **HRMS** (ESI-TOF) calc'd for for $C_{23}H_{33}N_2O_3$ $[M+H]^+$: 385.2480, found: 385.2482.

Isopropyl 2-(4-((4-(dimethylamino)phenyl)((3-methylpyridin-2-yl)methyl)amino)phenoxy)-2-methylpropanoate – (43)

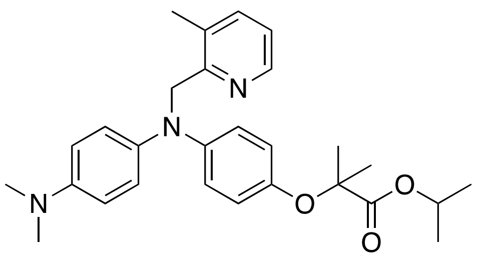

A solution of **40** (50 mg, 0.14 mmol), CH_2Cl_2 (2 mL) and acetic acid (0.01 mL) was prepared; 3-pyridine-methyl aldehyde (25 mg, 0.21 mmol) was then added. The resultant mixture was stirred at room temperature for 5 minutes before the addition of sodium triacetoxyborohydride (88 mg, 0.42 mmol). The reaction mixture was stirred at room temperature for 16 h. A mixture saturated sodium bicarbonate solution and CH_2Cl_2 (20 mL (1:1)) was added. The organic and aqueous phases were separated, and the aqueous phase was extracted with CH_2Cl_2 (2 x 20 mL). The organic fractions were combined, dried (Na_2SO_4) and the solvent was removed in *vacuo*. The crude mixture was then purified using flash column chromatography using (MeOH 0%- 20%, in CH_2Cl_2) over 20 column volumes to give **43** (27 mg, 0.0588 mmol, 42%) as a yellow oil.

^{1}H NMR (400 MHz, DMSO) δ 8.31 – 8.22 (m, 1H), 7.52 – 7.47 (m, 1H), 7.17 – 7.10 (m, 1H), 7.04 – 6.99 (m, 2H), 6.73 – 6.50 (m, 6H), 4.92 (d, *J* = 6.0 Hz, 1H), 4.86 (s, 2H), 2.83 (s, 6H), 2.27 (s, 3H), 1.41 (s, 6H), 1.17 (d, *J* = 6.0 Hz, 6H). **^{13}C NMR** (101 MHz, DMSO) δ 173.27, 156.63, 147.80, 147.21, 146.48, 145.73, 138.13, 137.76, 132.06, 126.87, 122.51, 121.05, 116.74, 113.77, 79.43, 68.71, 56.39, 40.93 (2C, $N(CH_3)_2$), 25.46 (2C, $C(CH_3)_2$), 21.80 (2C, $CH(CH_3)_2$), 18.32. **HRMS** (ESI-TOF) calc'd for $C_{28}H_{36}N_3O_3$ $[M+H]^+$: 462.2751, found: 462.2748.

Ethyl 6-((4-(dimethylamino)phenyl)(4-((1-isopropoxy-2-methyl-1-oxopropan-2-yl)oxy)phenyl)amino)nicotinate – (44)

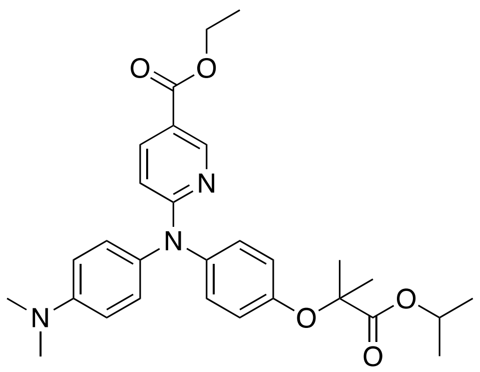

General Procedure B using **40** (50 mg, 0.14 mmol), RuPhos Pd G3 (11.5 mg, 0.014 mmol), Cs_2CO_3 (113 mg, 0.35 mmol), 6-chloro-ethylnicotinate (39 mg, 0.21 mmol) and tert-butanol (3 mL) gave **44** (19 mg, 0.0378 mmol, 27%) as a yellow oil.

^{1}H NMR (400 MHz, DMSO) δ 8.55 (dd, J = 2.4, 0.8 Hz, 1H), 7.89 (dd, J = 9.0, 2.4 Hz, 1H), 7.19 – 7.14 (m, 2H), 7.11 – 7.06 (m, 2H), 6.81 – 6.72 (m, 4H), 6.40 (dd, J = 9.0, 0.8 Hz, 1H), 4.95 (p, J = 6.3 Hz, 1H), 4.24 (q, J = 7.1 Hz, 2H), 2.90 (s, 6H), 1.52 (s, 6H), 1.26 (t, J = 7.1 Hz, 3H), 1.16 (d, J = 6.3 Hz, 6H). **^{13}C NMR** (101 MHz, DMSO) δ 172.97, 165.34, 161.56, 153.20, 150.46, 149.37, 139.23, 138.25, 133.44, 128.93, 128.46, 119.66, 113.70, 108.92, 79.29, 69.06, 60.57, 41.41 (2C, $N(CH_3)_2$), 25.55 (2C, $C(CH_3)_2$), 21.73 (2C, $CH(CH_3)_2$), 14.71. **HRMS** (ESI-TOF) calc'd for $C_{29}H_{36}N_3O_5$ $[M+H]^+$: 506.2649, found: 506.2644.

Isopropyl 2-(4-(*N*-(4-(dimethylamino)phenyl)acetamido)phenoxy)-2-methylpropanoate – (45)

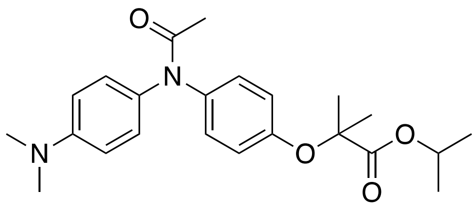

A solution of **40** (50 mg, 0.14 mmol), DIPEA (36 µL, 0.21 mmol) and anhydrous CH_2Cl_2 (2 mL) under anhydrous conditions was prepared; acetyl chloride (15 µL, 0.21 mmol) was then added. The reaction mixture was stirred at room temperature for 16 h. A mixture saturated $NaHCO_3$ solution and CH_2Cl_2 (20 mL (1:1)) was added. The organic and aqueous phases were separated, and the

aqueous phase was extracted with CH_2Cl_2 (2 x 20 mL). The organic fractions were combined, dried (Na_2SO_4) and the solvent was removed in *vacuo*. The crude mixture was then purified using flash column chromatography using (EtOAc 0% - 100%, in CH_2Cl_2) over 20 column volumes to give **45** (15 mg, 0.0378 mmol, 27%) as a yellow oil.

^{1}H NMR (400 MHz, DMSO) δ 7.38 – 6.98 (m, 4H), 6.85 – 6.60 (m, 4H), 4.94 (d, *J* = 6.0 Hz, 1H), 2.88 (s, 6H), 1.86 (s, 3H), 1.50 (s, 6H), 1.14 (d, *J* = 6.0 Hz, 6H). **^{13}C NMR** (101 MHz, DMSO) δ 219.00, 172.52, 152.62, 149.42, 137.64, 132.20, 128.83, 127.26, 118.54, 112.67, 78.75, 68.59, 25.03 (2C, $C(CH_3)_2$), 23.22, 21.26 (2C, $CH(CH_3)_2$). **HRMS** (ESI-TOF) calc'd for $C_{23}H_{31}N_2O_4$ $[M+H]^+$: 399.2273, found: 399.2275.

Isopropyl 2-(4-(1-(4-(dimethylamino)phenyl)-3-phenylureido)phenoxy)-2-methylpropanoate – (46)

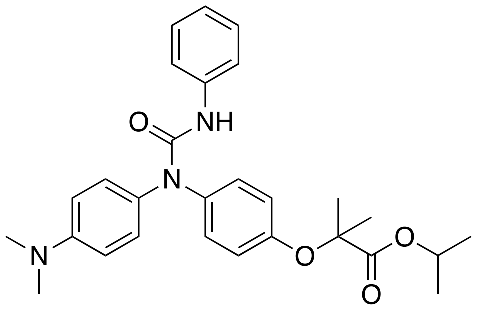

A solution of **40** (50 mg, 0.14 mmol), DIPEA (48 µL, 0.28 mmol) and *N,N*-dimethylacetamide (2 mL) under anhydrous conditions was prepared; phenyl isocyanate (28 mg, 0.21 mmol) was then added. The reaction mixture was stirred at room temperature for 16 h. A mixture saturated sodium bicarbonate solution and CH_2Cl_2 (20 mL (1:1)) was added. The organic and aqueous phases were separated, and the aqueous phase was extracted with CH_2Cl_2 (2 x 20 mL). The organic fractions were combined, dried (Na_2SO_4) and the solvent was removed in *vacuo*. The crude mixture was then purified using flash column chromatography using (EtOAc 0%- 100%, in CH_2Cl_2) over 20 column volumes to give **46** (10 mg, 0.021 mmol, 15%) as a yellow oil.

^{1}H NMR (400 MHz, DMSO) δ 7.82 (s, 1H), 7.42 – 7.37 (m, 2H), 7.23 – 7.18 (m, 2H), 7.13 – 7.06 (m, 4H), 6.98 – 6.90 (m, 1H), 6.79 – 6.69 (m, 4H), 4.96 (h, *J* = 6.0 Hz, 1H), 2.90 (s, 6H),

1.50 (s, 6H), 1.17 (d, $J = 6.0$ Hz, 6H). **^{13}C NMR** (101 MHz, DMSO) δ 172.56, 154.28, 152.35, 148.92, 139.74, 138.04, 131.31, 128.65, 128.25, 127.47, 122.15, 119.80, 118.91, 112.91, 78.80, 68.55, 25.05 (2C, $C(CH_3)_2$), 21.28 (2C, $CH(CH_3)_2$). **HRMS** (ESI-TOF) calc'd for $C_{28}H_{34}N_3O_4$ $[M+H]^+$: 476.2544, found: 476.2542.

Isopropyl 2-(4-(3-benzyl-1-(4-(dimethylamino)phenyl)ureido)phenoxy)-2-methylpropanoate – (47)

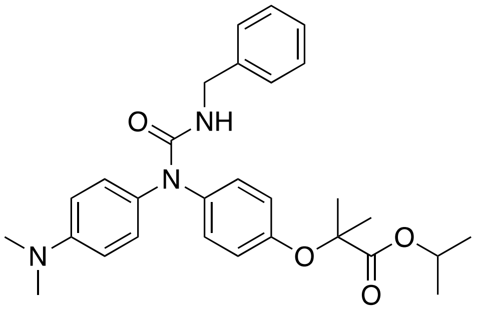

A solution of **40** (50 mg, 0.14 mmol), DIPEA (48 µL, 0.28 mmol) and *N,N*-dimethylacetamide (2 mL) under anhydrous conditions was prepared; benzyl isocyanate (31.5 mg, 0.23 mmol) was then added. The reaction mixture was stirred at room temperature for 16 h. A mixture saturated sodium bicarbonate solution and CH_2Cl_2 (20 mL (1:1)) was added. The organic and aqueous phases were separated, and the aqueous phase was extracted with CH_2Cl_2 (2 x 20 mL). The organic fractions were combined, dried (Na_2SO_4) and the solvent was removed in *vacuo*. The crude mixture was then purified using flash column chromatography using (EtOAc 0% - 100%, in CH_2Cl_2) over 20 column volumes to give **47** (13 mg, 0.0266 mmol, 19%) as a yellow oil.

^{1}H NMR (400 MHz, DMSO) δ 7.35 – 7.16 (m, 5H), 7.12 – 7.06 (m, 4H), 6.74 – 6.68 (m, 4H), 6.23 (t, $J = 6.0$ Hz, 1H), 4.94 (h, $J = 6.0$ Hz, 1H), 4.22 (d, $J = 6.0$ Hz, 2H), 2.89 (s, 6H), 1.48 (s, 6H), 1.17 (d, $J = 6.0$ Hz, 6H). **^{13}C NMR** (101 MHz, DMSO) δ 172.59, 156.36, 151.92, 148.98, 140.91, 138.35, 131.20, 129.07, 128.08, 127.16, 126.84, 126.35, 118.84, 112.98, 78.76, 68.53, 43.60, 25.02 (2C, $C(CH_3)_2$), 21.28 (2C, $CH(CH_3)_2$). **HRMS** (ESI-TOF) calc'd for $C_{29}H_{36}N_3O_4$ $[M+H]^+$: 490.2695, found: 490.2696.

Benzyl 4-(4-(4-((1-(cyclohexylamino)-2-methyl-1-oxopropan-2-yl)oxy)benzoyl)phenyl)piperazine-1-carboxylate – (48)

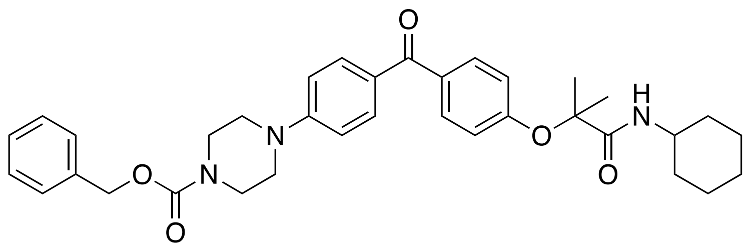

General Procedure B using **12** (70 mg, 0.175 mmol), RuPhosPdG3 (14.5 mg, 0.0175 mmol), Cs_2CO_3 (113 mg, 0.35 mmol), 1-cbz-piperazine (46 mg, 0.21 mmol) in ${}^tBuOH$ (0.1 M) gave **48** (59 mg, 0.101 mmol, 58%) as a yellow oil.

^{1}H NMR (400 MHz, DMSO) δ 7.81 (d, J = 8.3 Hz, 1H), 7.66 – 7.55 (m, 4H), 7.41 – 7.37 (m, 4H), 7.04 – 6.98 (m, 2H), 6.96 – 6.90 (m, 2H), 5.12 (s, 2H), 3.58 (dt, J = 18.3, 6.9 Hz, 6H), 3.41 – 3.35 (m, 5H), 1.70 – 1.55 (m, 4H), 1.50 (s, 6H), 1.24 – 1.15 (m, 4H), 1.08 – 0.99 (m, 1H). **^{13}C NMR** (101 MHz, DMSO) δ 192.64, 171.70, 158.16, 154.45, 153.20, 136.81, 131.71, 131.37, 130.92, 128.44, 127.89, 127.63, 126.56, 118.15, 113.36, 80.47, 66.36, 47.92, 46.42 (2C, $CON(CH_2)_2$), 42.98 (2C, $N(CH_2)_2$), 31.95 (2C, cyclohexane), 25.08 (2C, $C(CH_3)_2$), 24.84 (3C, cyclohexane). **HRMS** (ESI-TOF) calc'd for $C_{35}H_{42}O_5N_3$ $[M+H]^+$: 584.3119, found: 584.3121.

2-(4-(4-(4-Acetylpiperazin-1-yl)benzoyl)phenoxy)-*N*-cyclohexyl-2-methylpropanamide – (49)

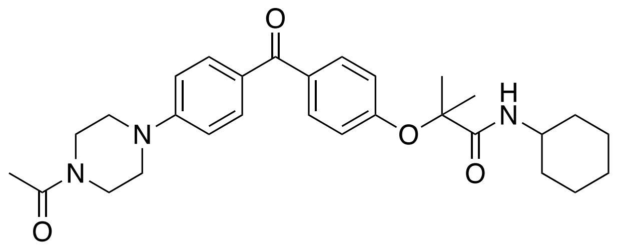

General Procedure B using **12** (70 mg, 0.175 mmol), RuPhosPdG3 (14.5 mg, 0.0175 mmol), Cs_2CO_3 (113 mg, 0.35 mmol), 1-acetly-piperazine (26 mg, 0.21 mmol) in ${}^tBuOH$ (0.1 M) gave **49** (29 mg, 0.0595 mmol, 34%) as a yellow oil.

^{1}H NMR (400 MHz, DMSO) δ 7.67 – 7.59 (m, 4H), 7.06 – 6.98 (m, 2H), 6.97 – 6.91 (m, 2H), 3.65 – 3.55 (m, 4H), 3.44 – 3.38 (m, 4H), 2.05 (s, 3H), 1.64 (dd, *J* = 11.1, 4.7 Hz, 4H), 1.50 (s, 6H), 1.30 – 0.96 (m, 7H). **^{13}C NMR** (101 MHz, DMSO) δ 192.60, 171.70, 168.39, 158.11, 153.15, 131.68, 131.42, 130.86, 129.22, 128.41, 126.46, 118.18, 113.19, 80.48, 47.91, 46.67, 46.37, 45.01, 40.35, 31.92 (2C, cyclohexane), 25.05 (2C, $C(CH_3)_2$), 24.81 (3C, cyclohexane), 21.15. **HRMS** (ESI-TOF) calc'd for $C_{29}H_{38}O_4N_3$ $[M+H]^+$: 492.2857, found: 492.2857.

2-(4-(4-(4-Acetylpiperazin-1-yl)benzoyl)phenoxy)-*N*,*N*,2-trimethylpropanamide – (50)

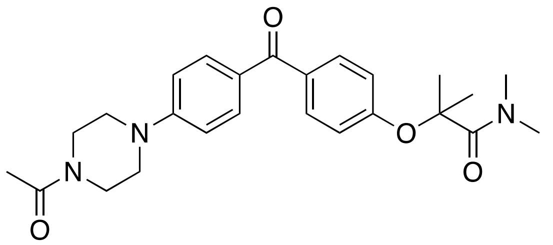

General Procedure B using **14** (100 mg, 0.289 mmol), RuPhosPdG3 (24 mg, 0.0289 mmol), Cs_2CO_3 (187 mg, 0.378 mmol), 1-acetylpiperazine (44 mg, 0.346 mmol) in tBuOH (0.1 M) gave **50** (47 mg, 0.106 mmol, 37%) as a yellow oil.

^{1}H NMR (400 MHz, DMSO) δ 7.69 – 7.60 (m, 4H), 7.05 – 6.97 (m, 2H), 6.92 – 6.83 (m, 2H), 3.62 – 3.53 (m, 4H), 3.40 (dd, *J* = 6.6, 3.9 Hz, 4H), 3.06 (s, 3H), 2.84 (s, 3H), 2.04 (s, 3H), 1.60 (s, 6H). **^{13}C NMR** (101 MHz, DMSO) δ 192.47, 171.19, 168.39, 158.12, 153.16, 131.72, 131.39, 131.29, 126.39, 116.11, 113.20, 80.91, 46.66, 46.37, 45.01, 40.35, 36.78 (2C, $N(CH_3)_2$), 25.49 (2C, $C(CH_3)_2$), 21.16. **HRMS** (ESI-TOF) calc'd for $C_{25}H_{32}O_4N_3$ $[M+H]^+$: 438.2387, found: 438.2390.

Benzyl 4-(4-(4-((1-(dimethylamino)-2-methyl-1-oxopropan-2-yl)oxy)benzoyl)phenyl)piperazine-1-carboxylate – (51)

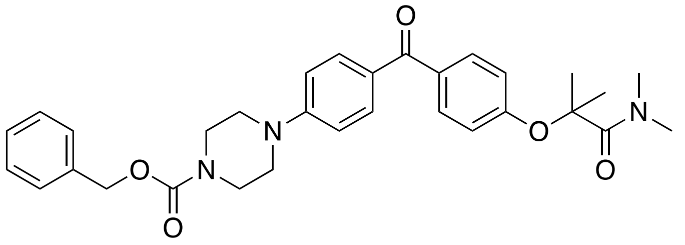

General Procedure B using **14** (100 mg, 0.289 mmol), RuPhosPdG3 (24 mg, 0.0289 mmol), Cs_2CO_3 (187 mg, 0.378 mmol), 1-cbz-piperazine (76 mg, 0.346 mmol) in ^tBuOH (0.1 M) gave **51** (66 mg, 0.124 mmol, 43%) as a yellow oil.

^{1}H NMR (400 MHz, DMSO) δ 7.69 – 7.58 (m, 4H), 7.42 – 7.29 (m, 5H), 7.05 – 6.99 (m, 2H), 6.92 – 6.84 (m, 2H), 5.12 (s, 2H), 3.60 – 3.52 (m, 4H), 3.42 – 3.35 (m, 4H), 3.06 (s, 3H), 2.84 (s, 3H), 1.60 (s, 6H). **^{13}C NMR** (101 MHz, DMSO) δ 192.50, 171.19, 158.13, 154.45, 153.18, 136.80, 131.72, 131.41, 131.27, 128.42, 127.87, 127.60, 126.49, 116.12, 113.34, 80.92, 66.34, 46.40 (2C, $CON(CH_2)_2$), 42.92 (2C, $N(CH_2)_2$), 36.80 (2C, $N(CH_3)_2$), 25.50 (2C, $C(CH_3)_2$). **HRMS** (ESI-TOF) calc'd for $C_{31}H_{36}O_5N_3$ $[M+H]^+$: 530.2649, found: 530.2652.

Benzyl 4-(4-(4-((1-(ethylamino)-2-methyl-1-oxopropan-2-yl)oxy)benzoyl)phenyl)piperazine-1-carboxylate – (52)

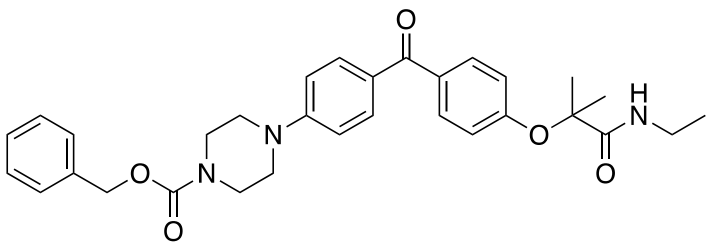

General Procedure B using **10** (70 mg, 0.2 mmol), RuPhosPdG3 (16.9 mg, 0.02 mmol), Cs_2CO_3 (130 mg, 0.40 mmol), 1-cbz-piperazine (52 mg, 0.24 mmol) in ^tBuOH (0.1 M) gave **52** (41 mg, 0.078 mmol, 39%) as a yellow oil.

^{1}H NMR (400 MHz, DMSO) δ 8.14 (t, J = 6.0 Hz, 1H), 7.63 (dd, J = 8.8, 1.6 Hz, 4H), 7.44 – 7.28 (m, 5H), 7.05 – 6.98 (m, 2H), 6.97 – 6.91 (m, 2H), 5.12 (s, 2H), 3.60 – 3.52 (m, 4H), 3.40 – 3.37 (m, 4H), 3.17 – 3.08 (m, 2H), 1.50 (s, 6H), 0.97 (t, J = 7.0 Hz, 3H). **^{13}C NMR** (101 MHz, DMSO) δ 192.65, 172.51, 158.12, 154.46, 153.21, 136.81, 131.74, 131.47, 130.95,

128.45, 127.91, 127.64, 126.53, 118.26, 113.37, 80.51, 66.37, 46.41 (2C, $CON(CH_2)_2$), 42.94 (2C, $N(CH_2)_2$), 33.63, 25.05 (2C, $C(CH_3)_2$), 14.51. **HRMS** (ESI-TOF) calc'd for $C_{31}H_{36}O_5N_3$ $[M+H]^+$: 530.2649, found: 530.2650.

Benzyl 4-(4-(4-((2-methyl-3-oxobutan-2-yl)oxy)benzoyl)phenyl)piperazine-1-carboxylate – (53)

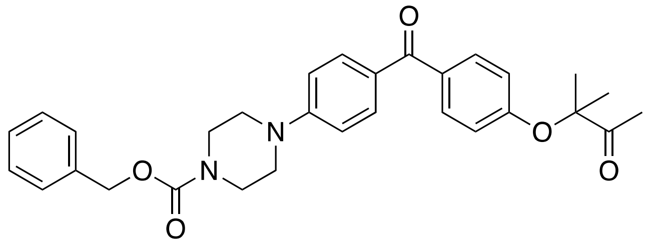

General Procedure B using **8** (100 mg, 0.316 mmol), RuPhosPdG3 (26 mg, 0.0316 mmol), Cs_2CO_3 (205 mg, 0.632 mmol), 1-cbz-piperazine (73 µL, 0.55 mmol) in tert-butanol (0.1 M) gave **53** (84 mg, 0.167 mmol, 53%) as a yellow oil.

^{1}H NMR (400 MHz, DMSO) δ 7.68 – 7.61 (m, 4H), 7.42 – 7.28 (m, 5H), 7.04 – 6.96 (m, 2H), 6.88 – 6.82 (m, 2H), 5.12 (s, 2H), 3.59 – 3.52 (m, 4H), 3.42 – 3.35 (m, 4H), 2.24 (s, 3H), 1.50 (s, 6H). **^{13}C NMR** (101 MHz, DMSO) δ 209.93, 192.53, 158.08, 154.46, 153.23, 136.81, 131.77, 131.48, 131.36, 128.87, 128.45, 127.90, 127.63, 126.47, 117.30, 113.36, 84.22, 66.37, 46.40 (2C, $CON(CH_2)_2$), 43.00 (2C, $N(CH_2)_2$), 26.34, 24.46 (2C, $C(CH_3)_2$). **HRMS** (ESI-TOF) calc'd for $C_{30}H_{33}O_5N_2$ $[M+H]^+$: 501.2384, found: 501.2386.

Benzyl 4-(4-((4-((1-isopropoxy-2-methyl-1-oxopropan-2-yl)oxy)phenyl)carbamoyl)phenyl)piperazine-1-carboxylate – (54)

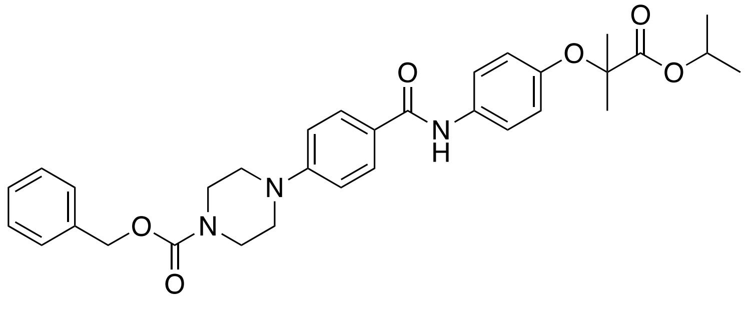

General Procedure B using **39** (100 mg, 0.266 mmol), RuPhos Pd G3 (22 mg, 0.0226 mmol), Cs_2CO_3 (172 mg, 0.53 mmol), 1-Z-piperazine (56 µL, 0.292 mmol) and tert-butanol (3 mL) gave **54** (46 mg, 0.0824 mmol, 31%) as a yellow oil.

^{1}H NMR (400 MHz, DMSO) δ 9.89 (s, 1H), 7.90 – 7.81 (m, 2H), 7.68 – 7.58 (m, 2H), 7.42 – 7.28 (m, 5H), 7.07 – 6.96 (m, 2H), 6.84 – 6.74 (m, 2H), 5.12 (s, 2H), 4.96 (hept, *J* = 6.0 Hz, 1H), 3.55 (s, 4H), 3.33 – 3.28 (m, 4H), 1.49 (s, 6H), 1.18 (d, *J* = 6.0 Hz, 6H). **^{13}C NMR** (101 MHz, DMSO) δ 172.75, 164.68, 154.46, 152.71, 150.74, 136.85, 134.02, 129.00, 128.47, 127.92, 127.64, 124.17, 121.36, 119.21, 113.92, 78.87, 68.50, 66.36, 46.98 (2C, CON(CH_2)$_2$), 43.07 (2C, N(CH_2)$_2$), 25.02 (2C, C(CH_3)$_2$), 21.33 (2C, CH(CH_3)$_2$). **HRMS** (ESI-TOF) calc'd for $C_{32}H_{37}N_3O_6$ $[M+H]^+$: 559.2682, found: 559.2690.

Isopropyl 2-(4-(4-(4-acetylpiperazin-1-yl)benzamido)phenoxy)-2-methylpropanoate – (55)

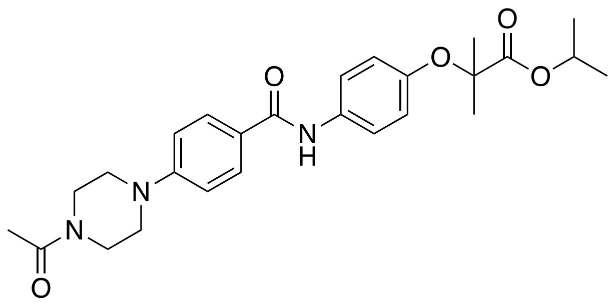

General Procedure B using **39** (40 mg, 0.106 mmol), RuPhos Pd G3 (22 mg, 0.0106 mmol), Cs_2CO_3 (86 mg, 0.265 mmol), 1-acetyl-piperazine (16.2 mg, 0.127 mmol) and tert-butanol (1.5 mL) gave **55** (7 mg, 0.0159 mmol, 15%) as a yellow oil.

^{1}H NMR (400 MHz, DMSO) δ 9.87 (s, 1H), 7.90 – 7.84 (m, 2H), 7.67 – 7.58 (m, 2H), 7.05 – 6.99 (m, 2H), 6.84 – 6.74 (m, 2H), 4.97 (hept, *J* = 6.0 Hz, 1H), 3.64 – 3.55 (m, 4H), 3.29 – 3.23 (m, 4H), 2.05 (s, 3H), 1.49 (s, 6H), 1.19 (d, *J* = 6.0 Hz, 6H). **^{13}C NMR** (101 MHz, DMSO) δ 172.69, 168.34, 164.66, 152.65, 150.71, 134.02, 128.96, 124.03, 121.31, 119.24, 113.73, 78.87, 68.45, 47.27 (2C, CON(CH_2)$_2$), 45.12 (2C, N(CH_2)$_2$), 24.99 (2C, C(CH_3)$_2$), 21.29 (2C, CH(CH_3)$_2$), 21.17. **HRMS** (ESI-TOF) calc'd for $C_{26}H_{34}N_3O_5$ $[M+H]^+$: 468.2493, found: 468.2494.

Ethyl 2-(4-(4-chlorobenzoyl)phenoxy)acetate – (56a)

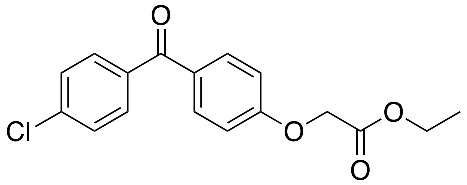

A mixture of (4-chlorophenyl)(4-hydroxyphenyl)methanone (1.1 g, 0.004 mol) and Cs_2CO_3 (3.0 g, 0.0094 mol) in *N,N*-dimethylacetamide (7 mL) was prepared before the addition of ethyl bromoacetate (534 µL, 0.0048 mol). The resultant mixture was heated at 100°C overnight. Water (30 mL) was then added, and the mixture was extracted with EtOAc (3 x 30 mL). The organic fractions were combined, washed with brine, dried (Na_2SO_4) and the solvent was removed in *vacuo*. The crude mixture was then purified using flash column chromatography using (EtOAc 0 %- 25%, in cyclohexane) over 10 column volumes gave **56a** (960 mg, 0.0030 mol, 75%) with the colourless oil.

^{1}H NMR (400 MHz, DMSO) δ 7.79 – 7.67 (m, 4H), 7.65 – 7.56 (m, 2H), 7.13 – 7.05 (m, 2H), 4.92 (s, 2H), 4.18 (q, *J* = 7.0 Hz, 2H), 1.22 (t, *J* = 7.0 Hz, 3H). **^{13}C NMR** (101 MHz, DMSO) δ 193.22, 168.22, 161.38, 137.04, 136.27, 132.04, 131.14, 129.68, 128.57, 114.51, 64.72, 60.78, 13.99. **HRMS** (ESI-TOF) calc'd for $C_{17}H_{16}O_4{}^{35}Cl$ $[M+H]^+$: 319.0732, found: 319.0742.

2-(4-(4-Chlorobenzoyl)phenoxy)acetic acid – (56b)

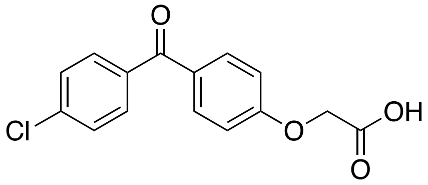

56a (920 mg, 2.89 mmol) was dissolved in a mixture of THF (50 mL) and H_2O (5 mL) before the addition of lithium hydroxide monohydrate (355 mg, 8.67 mmol). The resultant mixture was stirred for 16 hrs. H_2O (50 m:) was added to the reaction mixture before the THF was removed in *vacuo*. The aqueous mixture was extracted with EtOAc (50 mL) before being acidified to pH 3. The aqueous solution was then extracted with EtOAc (3 x 50 mL). The organic fractions were combined, dried (Na_2SO_4) and the solvent was removed in

vacuo. The crude mixture was then purified using flash column chromatography using (0 – 100% EtOAc, in cyclohexane (0.1% formic acid)) over 20 column volumes gave **56b** (746 mg, 2.57 mmol, 89%) with the colourless oil.

^{1}H NMR (400 MHz, DMSO) δ 13.05 (s, 1H), 7.78 – 7.59 (m, 6H), 7.18 – 7.02 (m, 2H), 4.82 (s, 2H). **^{13}C NMR** (101 MHz, DMSO) δ 193.27, 169.68, 161.59, 137.02, 136.34, 132.07, 131.17, 129.51, 128.59, 114.48, 64.59. **HRMS** (ESI-TOF) calc'd for $C_{15}H_{10}O_4{}^{35}Cl$ $[M+H]^-$: 289.0273, found: 289.0270.

2-(4-(4-Chlorobenzoyl)phenoxy)-*N*-cyclohexyl acetamide – (56c)

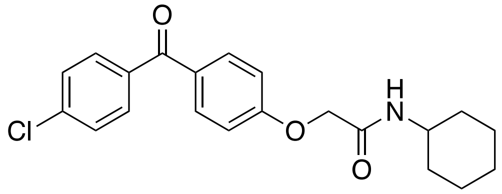

General Procedure A using **56b** (400 mg, 1.37 mmol), HATU (1.04 g, 2.75 mmol), DIPEA (706 µL, 4.11 mmol) and cyclohexylamine (235 µL, 2.05 mmol) gave **56c** (275 mg, 0.74 mmol, 54%) as a colourless oil.

^{1}H NMR (400 MHz, DMSO) δ 7.97 (d, *J* = 8.0 Hz, 1H), 7.79 – 7.64 (m, 4H), 7.64 – 7.54 (m, 2H), 7.13 – 7.02 (m, 2H), 4.58 (s, 2H), 3.63 (qd, *J* = 10.0, 4.0 Hz, 1H), 1.78 – 1.50 (m, 5H), 1.32 – 1.02 (m, 6H). **^{13}C NMR** (101 MHz, DMSO) δ 193.29, 165.89, 161.70, 137.03, 136.35, 132.04, 131.15, 129.51, 128.59, 114.66, 66.95, 47.52, 32.24 (2C, $(CH_2)_2$), 25.15, 24.63 (2C, $(CH_2)_2$). **HRMS** (ESI-TOF) calc'd for $C_{21}H_{23}NO_3{}^{35}Cl$ $[M+H]^+$: 372.1361, found: 372.1363.

Benzyl 4-(4-(4-(2-(cyclohexylamino)-2-oxoethoxy)benzoyl)phenyl)piperazine-1-carboxylate – (56)

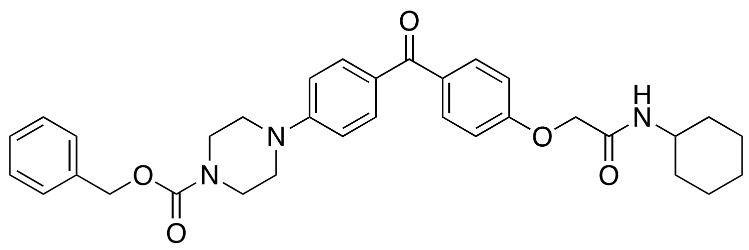

General Procedure B using **56c** (100 mg, 0.269 mmol), RuPhos Pd G3 (23 mg, 0.0269 mmol), Cs_2CO_3 (171 mg, 0.52 mmol), 1-Z-piperazine (62 µL, 0.322 mmol) and tert-butanol (3 mL) gave **57** (48 mg, 0.0860 mmol, 32%) as a yellow oil.

^{1}H NMR (400 MHz, DMSO) δ 7.94 (d, J = 8.0 Hz, 1H), 7.68 – 7.61 (m, 4H), 7.39 – 7.30 (m, 5H), 7.10 – 6.97 (m, 4H), 5.12 (s, 2H), 4.56 (s, 2H), 3.58 – 3.52 (m, 4H), 3.42 – 3.36 (m, 4H), 1.80 – 1.50 (m, 5H), 1.31 – 1.06 (m, 6H). **^{13}C NMR** (101 MHz, DMSO) δ 192.64, 166.02, 160.70, 154.45, 153.16, 136.80, 131.68, 131.36, 131.10, 128.42, 127.87, 127.59, 126.66, 114.33, 113.35, 66.95, 66.34, 47.48, 46.42 (2C, $CON(CH_2)_2$), 42.95 (2C, $N(CH_2)_2$), 32.23 (2C, $(CH_2)_2$), 25.13, 24.61 (2C, $(CH_2)_2$). **HRMS** (ESI-TOF) calc'd for $C_{33}H_{38}N_3O_3$ $[M+H]^+$: 556.2806, found: 556.2809.

2-(4-(4-(4-Acetylpiperazin-1-yl)benzoyl)phenoxy)-*N*-cyclohexylacetamide – (57)

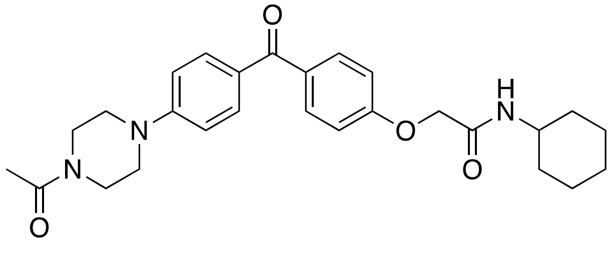

General Procedure B using **56c** (100 mg, 0.269 mmol), RuPhos Pd G3 (23 mg, 0.0269 mmol), Cs_2CO_3 (171 mg, 0.55 mmol), 1-acetyl-piperazine (41 mg, 0.322 mmol) and tert-butanol (3 mL) gave **56** (44 mg, 0.0945 mmol, 35%) as a yellow oil.

^{1}H NMR (400 MHz, DMSO) δ 7.69 – 7.61 (m, 4H), 7.09 – 6.99 (m, 4H), 4.56 (s, 2H), 3.61 – 3.54 (m, 4H), 3.43 – 3.38 (m, 4H), 2.05 (s, 3H), 1.75 – 1.50 (m, 7H), 1.31 – 1.19 (m, 4H). **^{13}C NMR** (101 MHz, DMSO) δ 192.62, 168.41, 166.02, 160.69, 153.13, 131.68, 131.35, 131.12, 126.56, 114.33, 113.21, 66.95, 47.49, 46.68, 46.39, 45.02, 40.36, 32.23 (2C, $(CH_2)_2$), 25.14, 24.62 (2C, $(CH_2)_2$), 21.16. **HRMS** (ESI-TOF) calc'd for $C_{27}H_{34}N_3O_4$ $[M+H]^+$: 464.2544, found: 464.2546.

2-(4-(4-Chlorobenzoyl)phenoxy)-*N*,*N*-dimethylacetamide – (58c)

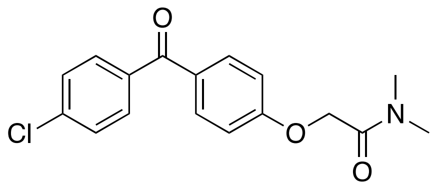

General Procedure A using **56b** (400 mg, 1.37 mmol), HATU (1.04 g, 2.75 mmol), DIPEA (706 µL, 4.11 mmol) and dimethylamine HCl (167 mg, 2.05 mmol) in *N,N*-dimethylacetamide (5 mL) gave **58c** (274 mg, 0.863 mmol, 63%) as a colourless oil.

^{1}H NMR (400 MHz, DMSO) δ 7.71 (m, 4H), 7.65 – 7.55 (m, 2H), 7.09 – 7.00 (m, 2H), 4.96 (s, 2H), 3.00 (s, 3H), 2.85 (s, 3H). **^{13}C NMR** (101 MHz, DMSO) δ 193.27, 166.58, 162.12, 150.90, 137.01, 136.40, 131.94, 131.17, 128.73, 128.58, 120.60, 114.62, 65.67, 35.48 (2C, $N(CH_3)_2$). **HRMS** (ESI-TOF) calc'd for $C_{17}H_{17}NO_3{}^{35}Cl$ $[M+H]^+$: 318.0891, found: 318.0892.

Benzyl 4-(4-(4-(2-(dimethylamino)-2-oxoethoxy)benzoyl)phenyl)piperazine-1-carboxylate – (58)

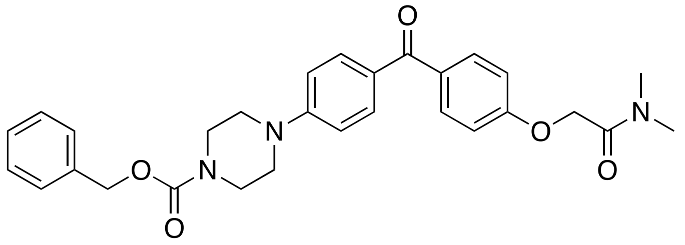

General Procedure B using **58c** (100 mg, 0.315 mmol), RuPhos Pd G3 (26 mg, 0.0315 mmol), Cs_2CO_3 (204 mg, 0.63 mmol), 1-Cbz-piperazine (73 µL, 0.378 mmol) and tert-butanol (3 mL) gave **58** (44 mg, 0.0882 mmol, 28%) as a yellow oil.

^{1}H NMR (400 MHz, DMSO) δ 7.66 – 7.62 (m, 4H), 7.40 – 7.30 (m, 5H), 7.04 – 6.99 (m, 4H), 5.12 (s, 2H), 4.93 (s, 2H), 3.59 – 3.51 (m, 4H), 3.41 – 3.37 (m, 4H), 3.00 (s, 3H), 2.86 (s, 3H). **^{13}C NMR** (101 MHz, DMSO) δ 192.64, 166.69, 161.10, 154.45, 153.15, 136.80, 131.68, 131.26, 130.80, 128.42, 127.87, 127.59, 126.72, 114.26, 113.35, 66.34, 65.65, 46.42 (2C, $CON(CH_2)_2$), 42.97 (2C, $N(CH_2)_2$), 34.95 (2C, $N(CH_3)_2$). **HRMS** (ESI-TOF) calc'd for $C_{29}H_{32}N_3O_5$ $[M+H]^+$: 502.2336, found: 502.2340.

2-(4-(4-(4-Acetylpiperazin-1-yl)benzoyl)phenoxy)-*N*,*N*-dimethylacetamide – (59)

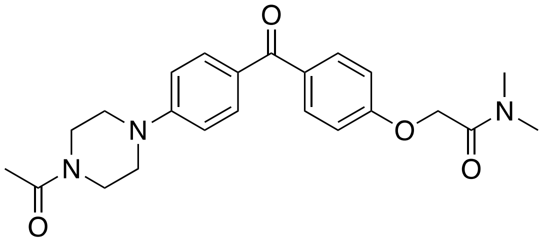

General Procedure B using **58c** (100 mg, 0.315 mmol), RuPhos Pd G3 (26 mg, 0.0315 mmol), Cs_2CO_3 (204 mg, 0.63 mmol), 1-acetyl-piperazine (48 mg, 0.378 mmol) and tert-butanol (3 mL) gave **59** (31 mg, 0.0756 mmol, 24%) as a yellow oil.

^{1}H NMR (400 MHz, DMSO) δ 7.66 – 7.61 (m, 4H), 7.04 – 6.99 (m, 4H), 4.93 (s, 2H), 3.64 – 3.53 (m, 4H), 3.47 – 3.38 (m, 4H), 3.01 (s, 3H), 2.86 (s, 3H), 2.05 (s, 3H). **^{13}C NMR** (101 MHz, DMSO) δ 192.64, 168.43, 166.70, 161.09, 153.14, 131.70, 131.26, 130.83, 126.61,

114.27, 113.22, 65.65, 46.69 (2C, CON(CH_2)$_2$), 45.02 (2C, N(CH_2)$_2$), 35.49, 34.96, 21.18. **HRMS** (ESI-TOF) calc'd for $C_{23}H_{28}N_3O_4$ [M+H]$^+$: 410.2074, found: 410.2077.

2-(4-Aminophenoxy)-*N*,*N*,2-trimethylpropanamide – (60b)

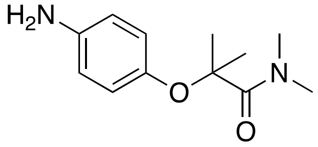

General Procedure A using 2-(4-Aminophenoxy)-2-methylpropanoic acid (3.165 g, 16.23 mmol), HATU (7.40 g, 19.45 mmol), DIPEA (8.37 mL, 48.6 mmol) and dimethylamine HCl (2.62 g, 32.46 mmol) gave **60b** (1981 mg, 8.92 mmol, 55%) as a brown oil.

^{1}H NMR (400 MHz, $CDCl_3$) δ 7.24 (d, *J* = 8.5 Hz, 2H), 6.75 (d, *J* = 8.5 Hz, 2H), 3.10 (s, 3H), 2.88 (s, 3H), 1.56 (s, 6H). **^{13}C NMR** (101 MHz, $CDCl_3$) δ 173.23, 155.51, 124.39, 118.16, 81.22, 37.67, 37.49, 25.71 (2C, C(CH_3)$_2$). **HRMS** (ESI-TOF) calc'd for $C_{12}H_{19}N_2O_2$ [M+H]$^+$: 223.1441, found: 223.1443.

Benzyl 4-(4-((4-((1-(dimethylamino)-2-methyl-1-oxopropan-2-yl)oxy)phenyl)carbamoyl)-phenyl)piperazine-1-carboxylate – (60)

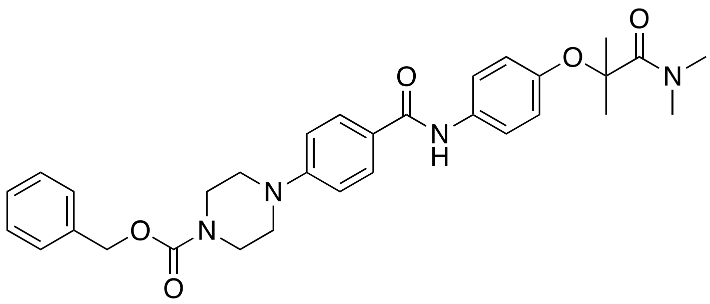

General Procedure A using 4-(4-((Benzyloxy)carbonyl)piperazin-1-yl)benzoic acid (100 mg, 0.29 mmol), HATU (134 mg, 0.35 mmol), DIPEA (150 µL, 0.87

mmol), **60b** (128 mg, 0.58 mmol) in *N,N*-dimethylformamide (50 mL) gave **60** (65 mg, 0.1189 mmol, 41%) as a yellow oil.

^{1}H NMR (400 MHz, DMSO) δ 9.85 (s, 1H), 7.89 – 7.82 (m, 2H), 7.65 – 7.58 (m, 2H), 7.41 – 7.29 (m, 5H), 7.05 – 6.98 (m, 2H), 6.77 – 6.71 (m, 2H), 5.12 (s, 2H), 3.62 – 3.51 (m, 4H), 3.14 (s, 3H), 2.86 (s, 3H), 1.51 (s, 6H). **^{13}C NMR** (101 MHz, DMSO) δ 171.82, 164.65, 154.44, 152.67, 150.82, 136.81, 133.55, 128.95, 128.42, 127.86, 127.58, 124.17, 121.73, 117.28, 113.88, 80.41, 68.50, 66.31, 46.96 (2C, CON(CH_2)$_2$), 43,07 (2C, N(CH_2)$_2$), 36.90 (2C, N(CH_3)$_2$), 25.55 (2C, C(CH_3)$_2$). **HRMS** (ESI-TOF) calc'd for $C_{31}H_{37}N_4O_5$ [M+H]$^+$: 545.2753, found: 545.2755.

4-(4-Acetylpiperazin-1-yl)-*N*-(4-((1-(dimethylamino)-2-methyl-1-oxopropan-2-yl)oxy)phenyl)benzamide – (61)

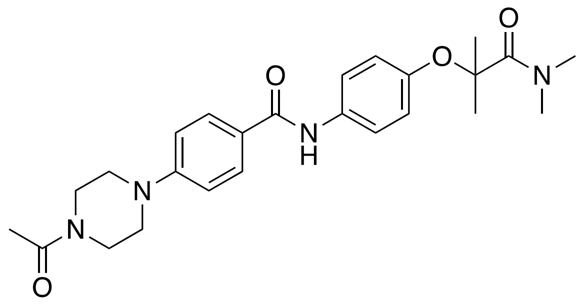

General Procedure A using 4-(4-Acetylpiperazin-1-yl)benzoic acid (100 mg, 0.40 mmol), HATU (183 mg, 0.48 mmol), DIPEA (206.4 µL, 1.2 mmol), **60b** (178 mg, 0.80 mmol) in *N,N*-dimethylformamide (50 mL) gave **61** (61 mg, 0.136 mmol, 34%) as a yellow oil.

^{1}H NMR (400 MHz, DMSO) δ 9.86 (s, 1H), 7.90 – 7.81 (m, 2H), 7.67 – 7.57 (m, 2H), 7.05 – 6.98 (m, 2H), 6.79 – 6.71 (m, 2H), 3.61 – 3.52 (m, 4H), 3.30 – 3.24 (m, 4H), 3.14 (s, 3H), 2.86 (s, 3H), 2.05 (s, 3H), 1.52 (s, 6H). **^{13}C NMR** (101 MHz, DMSO) δ 171.81, 168.32, 164.66, 152.64, 150.81, 133.56, 128.94, 124.05, 121.71, 117.28, 113.74, 80.41, 47.27, 46.92, 45.11, 40.39, 36.90 (2C, N(CH_3)$_2$), 25.54 (2C, C(CH_3)$_2$), 21.16. **HRMS** (ESI-TOF) calc'd for $C_{25}H_{33}N_4O_4$ [M+H]$^+$: 453.2491, found: 453.2493.

2-(4-Aminophenoxy)-*N*-cyclohexyl-2-methylpropanamide – (62b)

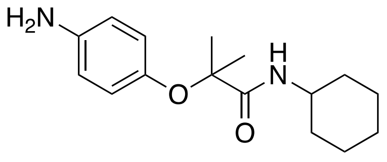

General Procedure A using 2-(4-Aminophenoxy)-2-methylpropanoic acid (500 mg, 2.56 mmol), HATU (1169 mg, 3.07 mmol), DIPEA (661 µL, 3.84 mmol), cyclohexylamine (1.2 mL, 12.8 mmol) in *N,N*-dimethylformamide (20 mL) gave **62b** (473 mg, 1.71 mmol, 67%) as a colourless oil.

^{1}H NMR (400 MHz, DMSO) δ 10.20 (s, 2H), 7.79 (d, *J* = 8.3 Hz, 1H), 7.33 – 7.25 (m, 2H), 7.00 – 6.90 (m, 2H), 3.58 (td, *J* = 9.5, 4.9 Hz, 1H), 1.70 – 1.60 (m, 4H), 1.55 (dt, *J* = 15.4, 4.5 Hz, 1H), 1.42 (s, 6H), 1.29 – 1.14 (m, 4H), 1.08 – 0.97 (m, 1H). **^{13}C NMR** (101 MHz, DMSO) δ 171.89, 154.24, 125.75, 123.88, 120.30, 80.38, 47.86, 31.98 (2C, $(CH_2)_2$), 25.10 (2C, $C(CH_3)_2$), 24.93, 24.84. **HRMS** (ESI-TOF) calc'd for $C_{16}H_{25}N_2O_2$ $[M+H]^+$: 277.1911, found: 277.1912.

4-(4-Acetylpiperazin-1-yl)-*N*-(4-((1-(cyclohexylamino)-2-methyl-1-oxopropan-2-yl)oxy)phenyl)-benzamide – (62)

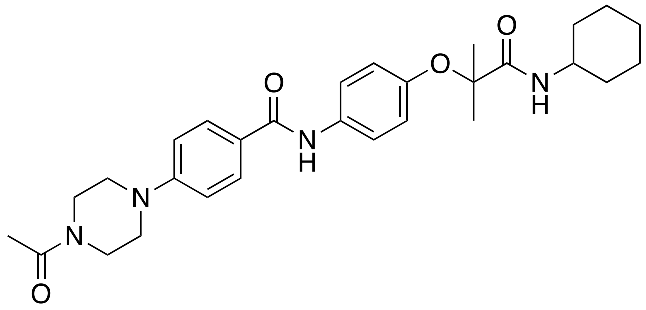

General Procedure A using 4-(4-Acetylpiperazin-1-yl)benzoic acid (250 mg, 1.01 mmol), HATU (766 mg, 2.01 mmol), DIPEA (434 µL, 2.52 mmol), **62b** (418 mg, 1.51 mmol) in *N,N*-dimethylformamide (10 mL) gave **62** (184 mg, 0.363 mmol, 36%) as a colourless oil.

^{1}H NMR (400 MHz, DMSO) δ 9.86 (s, 1H), 7.90 – 7.83 (m, 2H), 7.69 – 7.59 (m, 2H), 7.08 – 6.98 (m, 2H), 6.90 – 6.78 (m, 2H), 3.63 – 3.56 (m, 5H), 3.29 – 3.25 (m, 2H), 2.05 (s, 3H), 1.75 – 1.50 (m, 7H), 1.39 (s, 6H), 1.30 – 1.20 (m, 4H). **^{13}C NMR** (101 MHz, DMSO) δ 172.48,

168.34, 164.67, 152.66, 150.34, 134.20, 128.96, 124.03, 121.17, 120.31, 113.74, 80.22, 47.79, 47.26, 46.92, 45.13, 40.40, 32.03 (2C, $(CH_2)_2$), 25.12 (2C, $N(CH_2)_2$), 24.99, 24.84, 21.72. **HRMS** (ESI-TOF) calc'd for $C_{29}H_{39}N_4O_4$ $[M+H]^+$: 507.2960, found: 507.2983.

2-(4-Chlorophenoxy)-*N*,*N*,2-trimethylpropanamide – (63b)

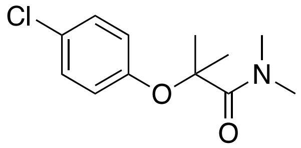

General Procedure A using **63a** (500 mg, 2.33 mmol), HATU (1.70 g, 4.67 mmol), DIPEA (1.2 mL, 0.87 mmol), dimethylamine (128 mg, 0.58 mmol) in *N,N*-dimethylformamide (50 mL) gave **63b** (470 mg, 1.95 mmol, 84%) as a colourless oil.

^{1}H NMR (400 MHz, DMSO) δ 7.34 – 7.28 (m, 2H), 6.81 – 6.75 (m, 2H), 3.07 (s, 3H), 2.83 (s, 3H), 1.52 (s, 6H). **^{13}C NMR** (101 MHz, DMSO) δ 171.30, 153.94, 129.35, 129.01, 125.23, 120.18, 118.60, 80.71, 36.78 (2C, $N(CH_3)_2$), 25.37 (2C, $C(CH_3)_2$). **HRMS** (ESI-TOF) calc'd for $C_{12}H_{17}NO_2{}^{35}Cl$ $[M+H]^+$: 242.0942, found: 242.0942.

Benzyl 4-(4-((4-((1-(dimethylamino)-2-methyl-1-oxopropan-2-yl)oxy)phenyl)amino)-phenyl)piperazine-1-carboxylate – (63c)

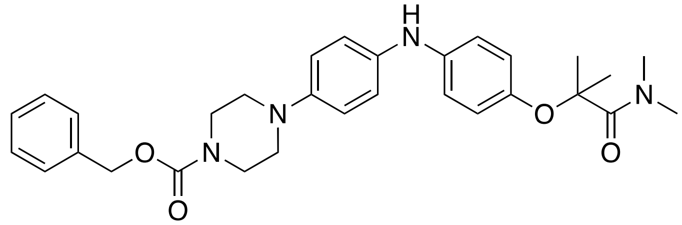

General Procedure B using Benzyl 4-(4-aminophenyl)piperazine-1-

carboxylate (250 mg, 0.80 mmol), BrettPhos Pd G3 (72 mg, 0.080 mmol), Cs_2CO_3 (520 mg, 1.6 mmol), **63b** (290 mg, 1.20 mmol) and tert-butanol (10 mL) gave **63c** (239 mg, 0.464 mmol, 58%) as a blue oil.

^{1}H NMR (400 MHz, $CDCl_3$) δ 7.41 – 7.30 (m, 5H), 6.98 – 6.90 (m, 2H), 6.88 – 6.83 (m, 4H), 6.73 – 6.68 (m, 2H), 5.34 (s, 1H), 5.16 (s, 2H), 3.70 – 3.63 (m, 4H), 3.24 (s, 3H), 3.09 – 3.01 (m, 4H), 2.97 (s, 3H), 1.59 (s, 6H). **^{13}C NMR** (151 MHz, DMSO) δ 172.01, 154.24, 149.50, 144.36, 128.51, 128.03, 127.78, 121.78, 120.19, 118.71, 115.41, 80.54, 66.79, 53.47 (2C, $CON(CH_2)_2$), 41.26 (2C, $N(CH_2)_2$), 36.98 (2C, $N(CH_3)_2$), 25.63 (2C, $C(CH_3)_2$). **HRMS** (ESI-TOF) calc'd for $C_{30}H_{36}N_4O_4$ $[M+H]^+$: 516.2737, found: 516.2738.

Benzyl 4-(4-((4-((1-(dimethylamino)-2-methyl-1-oxopropan-2-yl)oxy)phenyl)(methyl)amino)phenyl)piperazine-1-carboxylate – (63)

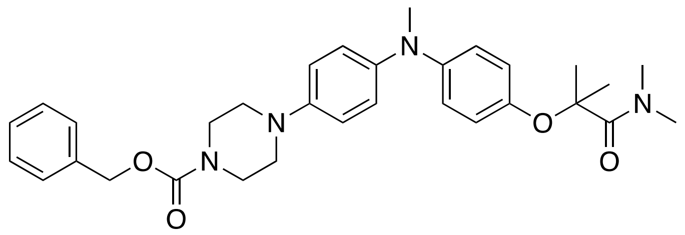

A solution of **63c** (70 mg, 0.135 mmol), CH_2Cl_2 (2 mL) and acetic acid (0.01 mL) was prepared; paraformaldehyde (6 mg, 0.20 mmol) was then added. The resultant mixture was heated to 50°C for 10 minutes before the addition of sodium triacetoxyborohydride (88 mg, 0.42 mmol). The reaction mixture was stirred at room temperature for 16 h. A mixture saturated sodium bicarbonate solution and CH_2Cl_2 (20 mL (1:1)) was added. The organic and aqueous phases were separated, and the aqueous phase was extracted with CH_2Cl_2 (2 x 20 mL). The organic fractions were combined, dried (Na_2SO_4) and the solvent was removed in *vacuo*. The crude mixture was then purified using flash column chromatography using (MeOH 0% - 20%, in CH_2Cl_2) over 20 column volumes to give **63** (44 mg, 0.0837 mmol, 62%) as a purple oil.

1H NMR (400 MHz, DMSO) δ 7.38 (d, *J* = 4.0 Hz, 5H), 6.90 (s, 4H), 6.77 – 6.73 (m, 2H), 6.68 – 6.63 (m, 2H), 5.10 (s, 2H), 3.53 (d, *J* = 7.0 Hz, 4H), 3.18 (s, 3H), 3.12 (s, 3H), 3.04 (t, *J* = 5.2 Hz, 4H), 2.85 (s, 3H), 1.46 (s, 6H). **^{13}C NMR** (101 MHz, DMSO) δ 172.03, 154.41, 148.33, 146.30, 144.27, 141.92, 136.86, 128.44, 127.87, 127.59, 122.62, 119.00, 118.73, 117.47, 80.49, 66.28, 49.13 (2C, $CON(CH_2)_2$), 43.47 (2C, $N(CH_2)_2$), 40.41, 36.93 (2C, $N(CH_3)_2$), 25.60 (2C, $C(CH_3)_2$). **HRMS** (ESI-TOF) calc'd for $C_{31}H_{39}N_4O_4$ $[M+H]^+$: 531.2971, found: 531.2973.

2-(4-((4-(4-Acetylpiperazin-1-yl)phenyl)amino)phenoxy)-*N*,*N*,2-trimethylpropanamide – (64c)

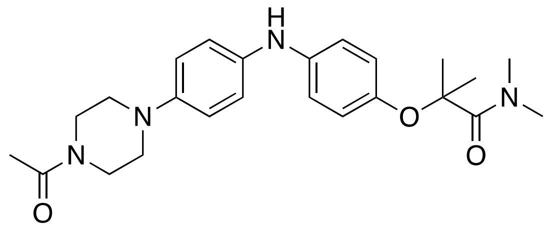

General Procedure B using 1-(4-(4-Aminophenyl)piperazin-1-yl)ethan-1-one (250 mg, 1.14 mmol), BrettPhos Pd G3 (103 mg, 0.114 mmol), Cs_2CO_3 (741 mg, 2.28 mmol), **64c** (412 mg, 1.71 mmol) and tert-butanol (10 mL) gave **1150** (309 mg, 0.729 mmol, 64%) as a purple oil.

^{1}H NMR (400 MHz, DMSO) δ 7.78 – 7.45 (m, 1H), 7.14 – 6.88 (m, 4H), 6.84 – 6.36 (m, 2H), 4.04 – 3.78 (m, 4H), 3.57 – 3.29 (m, 4H), 3.17 (s, 3H), 2.86 (s, 3H), 2.08 (s, 2H), 1.49 (s, 6H). **^{13}C NMR** (151 MHz, DMSO) δ 172.03, 168.71, 149.80, 145.37, 136.33, 133.36, 122.23, 120.60, 119.26, 118.70, 115.79, 115.08, 80.59, 54.30 (2C, $CON(CH_2)_2$), 43.23 (2C, $N(CH_2)_2$), 37.07 (2C, $N(CH_3$ (2C, $N(CH_2)_2$), 25.66 (2C, $C(CH_3)_2$), 21.12. **HRMS** (ESI-TOF) calc'd for $C_{24}H_{33}N_4O_3$ $[M+H]^+$: 425.2553, found: 425.2554.

2-(4-((4-(4-Acetylpiperazin-1-yl)phenyl)(methyl)amino)phenoxy)-*N*,*N*,2-trimethylpropanamide – (64)

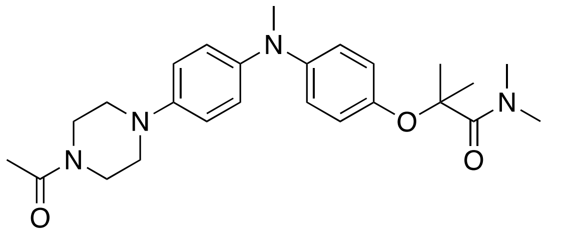

A solution of **64c** (70 mg, 0.165 mmol), CH_2Cl_2 (2 mL) and acetic acid (0.1 mL) was prepared; paraformaldehyde (6.3 mg, 0.21 mmol) was then added. The resultant mixture was heated to 50°C for 10 minutes before the addition of sodium triacetoxyborohydride (104 mg, 0.495 mmol). The reaction mixture was stirred at room temperature for 16 h. A mixture saturated sodium bicarbonate solution and CH_2Cl_2 (20 mL (1:1)) was added. The organic and aqueous phases were separated, and the aqueous phase was extracted with CH_2Cl_2 (2 x 20 mL). The organic fractions were combined, dried (Na_2SO_4) and the solvent was removed in *vacuo*. The crude mixture was then purified using flash column chromatography using (MeOH 0% - 20%, in CH_2Cl_2) over 20 column volumes to give **64** (31 mg, 0.070 mmol, 43%) as a colourless oil. **^{1}H NMR** (400 MHz, DMSO) δ 6.91 (s, 4H), 6.78 – 6.72 (m, 2H), 6.69 – 6.62 (m, 2H), 3.56 (q, *J* = 5.0 Hz, 4H), 3.18 (s, 3H), 3.13 (s, 3H), 3.07 (t, *J* = 5.0 Hz, 2H), 3.00 (t, *J* = 5.0 Hz, 2H), 2.85 (s, 3H), 2.03 (s, 3H), 1.46 (s, 6H). **^{13}C NMR** (101 MHz, DMSO) δ 172.01, 168.20, 148.29, 146.27, 144.28, 141.81, 122.65, 118.93, 118.73, 117.31, 80.48, 49.44 (2C, $CON(CH_2)_2$), 45.56 (2C, $N(CH_2)_2$), 40.75, 37.00 (2C, $N(CH_3)_2$), 25.58 (2C, $C(CH_3)_2$), 21.16. **HRMS** (ESI-TOF) calc'd for $C_{25}H_{35}N_4O_4$ $[M+H]^+$: 439.2709, found: 439.2709.

2-(4-((4-(Dimethylamino)phenyl)(methyl)amino)phenoxy)-*N*,*N*,2-trimethylpropanamide – (65)

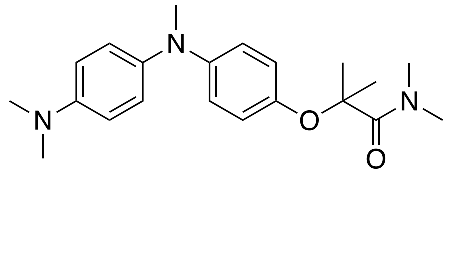

General Procedure B using N^1,N^1,N^4-Trimethylbenzene-1,4-diamine (50 mg, 0.33 mmol), RuPhos Pd G3 (25 mg, 0.033 mmol), Cs_2CO_3 (214 mg,

0.66 mmol), **1140** (119 mg, 0.495 mmol) and tert-butanol (3 mL) gave **65** (43.37 mg, 0.122 mmol, 37%) as a yellow oil.

^{1}H NMR (400 MHz, $CDCl_3$) δ 6.96 – 6.90 (m, 2H), 6.75 – 6.70 (m, 2H), 6.62 (s, 4H), 3.20 (s, 3H), 3.11 (s, 3H), 2.86 (s, 9H), 1.45 (s, 6H). **^{13}C NMR** (101 MHz, $CDCl_3$) δ 172.10, 147.27, 147.16, 145.03, 138.74, 124.93, 118.81, 116.55, 113.63, 80.44, 40.54 (3C, $N(CH_3)_2$ & NCH_3), 37.02 (2C, $N(CH_3)_2$), 25.58 (2C, $C(CH_3)_2$). **HRMS** (ESI-TOF) calc'd for $C_{21}H_{30}N_3O_2$ $[M+H]^+$: 356.2327, found: 356.2329.

2-(4-Chlorophenoxy)-*N*-cyclohexyl-2-methylpropanamide – (66b)

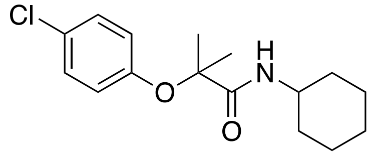

General Procedure A using **63a** (500 mg, 2.33 mmol), HATU (1700 mg, 4.67 mmol), DIPEA (1.2 mL, 6.99 mmol), cyclohexylamine (319 µL, 2.796 mmol) in N,N-dimethylacetamide (10 mL) gave **66b** (467 mg, 1.58 mmol, 68%) as a colourless oil.

^{1}H NMR (400 MHz, DMSO) δ 7.77 (d, *J* = 8.5 Hz, 1H), 7.35 – 7.26 (m, 2H), 6.92 – 6.83 (m, 2H), 3.58 (td, *J* = 14.0, 8.0 Hz, 1H), 1.71 – 1.49 (m, 5H), 1.41 (s, 6H), 1.31 – 0.97 (m, 5H). **^{13}C NMR** (101 MHz, DMSO) δ 171.91, 153.73, 128.88, 125.78, 121.22, 80.41, 47.84, 31.96 (2C, $(CH_2)_2$), 24.88 (2C, $C(CH_3)_2$), 24.83 (2C, $(CH_2)_2$). **HRMS** (ESI-TOF) calc'd for $C_{16}H_{23}NO_2{}^{35}Cl$ $[M+H]^+$: 296.1412, found: 296.1417.

***N*-Cyclohexyl-2-(4-((4-(dimethylamino)phenyl)(methyl)amino)phenoxy)-2-methylpropanamide – (66)**

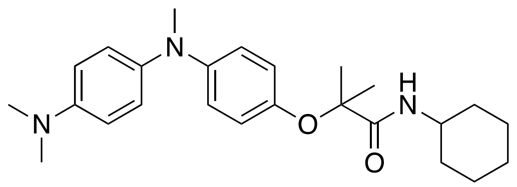

General Procedure B using *N*^{1},*N*^{1},*N*^{4}-Trimethylbenzene-1,4-diamine (50 mg, 0.33 mmol), RuPhos Pd G3 (25 mg, 0.033 mmol),

Cs_2CO_3 (214 mg, 0.66 mmol), **66b** (146 mg, 0.495 mmol) and tert-butanol (3 mL) gave **66** (76 mg, 0184 mmol, 56%) as an yellow oil.

^{1}H NMR (400 MHz, DMSO) δ 7.63 (d, *J* = 8.0 Hz, 1H), 6.97 – 6.91 (m, 2H), 6.78 – 6.69 (m, 4H), 6.64 – 6.59 (m, 2H), 3.64 – 3.53 (m, 1H), 3.11 (s, 3H), 2.86 (s, 6H), 1.74 – 1.52 (m, 6H), 1.32 (s, 6H), 1.30 – 1.01 (m, 4H). **^{13}C NMR** (101 MHz, DMSO) δ 172.76, 147.33, 146.39, 145.60, 138.65, 125.12, 121.81, 115.85, 113.60, 80.26, 47.70, 40.50 (2C, $N(CH_3)_2$), 32.05 (2C, $(CH_2)_2$), 25.12 (2C, $C(CH_3)_2$), 24.99, 24.82. **HRMS** (ESI-TOF) calc'd for $C_{25}H_{36}N_3O_2$ $[M+H]^+$: 410.2802, found: 410.2808.

2-(4-((4-(4-Acetylpiperazin-1-yl)phenyl)amino)phenoxy)-*N*-cyclohexyl-2-methylpropanamide – (67c)

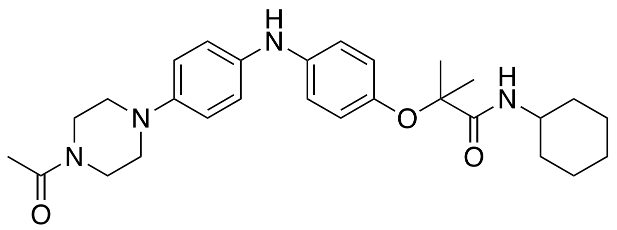

General Procedure B using 1-(4-(4-Aminophenyl)piperazin-1-yl)ethan-1-one (250 mg, 1.14 mmol), BrettPhos Pd G3 (103 mg, 0.114 mmol), Cs_2CO_3 (741 mg, 2.28 mmol), **66b** (504 mg, 1.71 mmol) and tert-butanol (10 mL) gave **67c** (266 mg, 0.558 mmol, 49%) as a purple oil.

^{1}H NMR (400 MHz, $CDCl_3$) δ 7.03 – 6.97 (m, 2H), 6.92 – 6.79 (m, 6H), 6.71 (d, *J* = 8.0 Hz, 1H), 5.48 (s, 1H), 3.84 – 3.71 (m, 3H), 3.68 – 3.56 (m, 2H), 3.08 (dq, *J* = 13.7, 5.0 Hz, 4H), 2.13 (s, 3H), 1.97 – 1.57 (m, 6H), 1.44 (s, 6H), 1.40 – 1.12 (m, 4H). **^{13}C NMR** (101 MHz, $CDCl_3$) δ 173.84, 168.80, 147.46, 145.77, 140.16, 137.00, 122.71, 119.87, 118.28, 117.38, 81.38, 50.59, 50.23, 47.72, 46.19, 41.30, 32.81 (2C, $(CH_2)_2$), 25.34 (2C, $C(CH_3)_2$), 24.84, 24.65 (2C, $(CH_2)_2$), 21.14. **HRMS** (ESI-TOF) calc'd for $C_{28}H_{39}N_4O_3$ $[M+H]^+$: 479.3022, found: 479.3024.

2-(4-((4-(4-Acetylpiperazin-1-yl)phenyl)(methyl)amino)phenoxy)-*N*-cyclohexyl-2-methylpropanamide – (67)

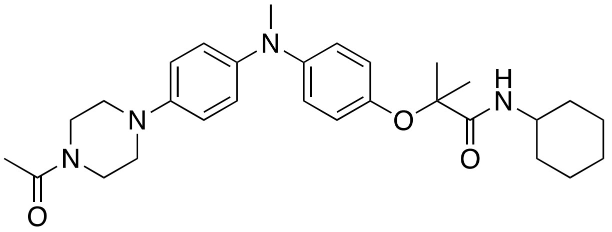

A solution of **67c** (70 mg, 0.165 mmol), CH_2Cl_2 (2 mL) and acetic acid (0.01 mL) was prepared; paraformaldehyde (6.3 mg, 0.21 mmol) was then added. The resultant mixture was heated to 50°C for 10 minutes before the addition of sodium triacetoxyborohydride (104 mg, 0.495 mmol). The reaction mixture was stirred at room temperature for 16 h. A mixture saturated sodium bicarbonate solution and CH_2Cl_2 (20 mL (1:1)) was added. The organic and aqueous phases were separated, and the aqueous phase was extracted with CH_2Cl_2 (2 x 20 mL). The organic fractions were combined, dried (Na_2SO_4) and the solvent was removed in *vacuo*. The crude mixture was then purified using flash column chromatography using (MeOH 0% - 20%, in CH_2Cl_2) over 20 column volumes to give **1155** (28 mg, 0.0561 mmol, 34%) as a colourless oil.

^{1}H NMR (400 MHz, DMSO) δ 7.65 (d, *J* = 8.0 Hz, 1H), 6.92 (s, 4H), 6.83 – 6.71 (m, 4H), 3.56 (q, *J* = 5.0 Hz, 4H), 3.13 (s, 3H), 3.07 (t, *J* = 5.0 Hz, 2H), 3.01 (t, *J* = 5.0 Hz, 2H), 2.03 (s, 3H), 1.71 – 1.51 (m, 6H), 1.34 (s, 6H), 1.30 – 1.01 (m, 5H). **^{13}C NMR** (101 MHz, DMSO) δ 172.66, 168.21, 147.59, 146.39, 144.85, 141.73, 122.92, 121.65, 118.17, 117.28, 80.27, 49.39, 49.02, 47.72, 45.56, 40.75, 32.04 (2C, $(CH_2)_2$), 25.12 (2C, $C(CH_3)_2$), 25.02, 24.82 (2C, $(CH_2)_2$), 21.16. **HRMS** (ESI-TOF) calc'd for $C_{29}H_{41}N_4O_3$ $[M+H]^+$: 493.3179, found: 493.3180.

***tert*-Butyl 4-(4-((4-((1-(dimethylamino)-2-methyl-1-oxopropan-2-yl)oxy)phenyl)-carbamoyl)-phenyl)-piperazine-1-carboxylate – (68b)**

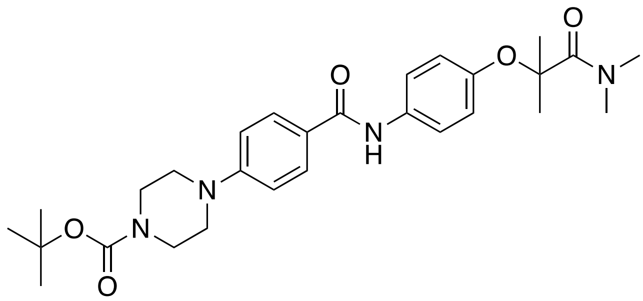

General Procedure A using 4-(4-(*tert*-Butoxycarbonyl)piperazin-1-yl)benzoic

acid (2.5 g, 8.17 mmol), HATU (6.2 g, 16.3 mmol), DIPEA (2.80 mL, 16.3 mmol), Feno-19 (2.17 g, 9.80 mmol) in *N,N*-dimethylformamide (100 mL) gave **68b** (1875 mg, 3.67 mmol, 45%) as a colourless oil.

^{1}H NMR (400 MHz, DMSO) δ 8.75 (s, 1H), 7.96 – 7.80 (m, 2H), 7.67 – 7.56 (m, 2H), 7.11 – 6.99 (m, 2H), 6.78 – 6.70 (m, 2H), 3.55 – 3.40 (m, 4H), 3.30 – 3.21 (m, 4H), 3.14 (s, 3H), 2.86 (s, 3H), 1.52 (d, J = 1.0 Hz, 6H), 1.42 (s, 9H). **^{13}C NMR** (151 MHz, DMSO) δ 171.86, 164.70, 153.88, 152.78, 151.99, 150.85, 133.58, 128.98, 124.14, 121.84, 121.77, 117.30, 114.30, 113.92, 80.44, 79.09, 47.06 (2C, CON(CH_2)$_2$), 42.55 (2C, N(CH_2)$_2$), 36.94 (2C, N(CH_3)$_2$), 28.08 (3C, C(CH_3)$_3$), 25.58 (2C, C(CH_3)$_2$). **HRMS** (ESI-TOF) calc'd for $C_{28}H_{39}N_4O_5$ [M+H]$^+$: 511.2915, found: 511.2914.

***N*-(4-((1-(Dimethylamino)-2-methyl-1-oxopropan-2-yl)oxy)phenyl)-4-(piperazin-1-yl)benzamide – (68c)**

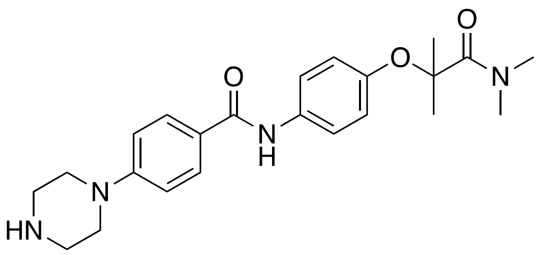

68b (1.85 g, 3.67 mmol) was dissolved in dichloromethane (CH_2Cl_2, 50 mL) and trifluoroacetic acid (25 mL). The resultant mixture was stirred at room temperature for 3 h. The mixture was concentrated under vacuum and was purified using flash reverse phase chromatography (95% - 5% H_2O (0.05N HCl), 5% - 95% acetonitrile) to give **68c** (1.41 g, 3.44 mmol, 94%).

^{1}H NMR (400 MHz, DMSO) δ 9.91 (s, 1H), 9.04 – 8.92 (m, 1H), 7.91 – 7.87 (m, 2H), 7.64 – 7.58 (m, 2H), 7.12 – 7.02 (m, 2H), 6.78 – 6.70 (m, 2H), 3.55 – 3.47 (m, 4H), 3.27 – 3.19 (m, 4H), 3.14 (s, 3H), 2.86 (s, 3H), 1.52 (s, 6H). **^{13}C NMR** (101 MHz, DMSO) δ 171.82, 164.54, 151.97, 150.90, 133.47, 129.03, 125.02, 121.81, 117.30, 114.26, 80.43, 53.48, 44.43, 42.44,

41.72, 36.92 (2C, $N(CH_2)_2$), 25.56 (2C, $C(CH_3)_2$). **HRMS** (ESI-TOF) calc'd for $C_{23}H_{31}N_4O_3$ $[M+H]^+$: 411.2391, found: 411.2393.

N-(4-((1-(Dimethylamino)-2-methyl-1-oxopropan-2-yl)oxy)phenyl)-4-(4-(2-(4-(trifluoromethyl)phenyl)acetyl)piperazin-1-yl)benzamide – (68)

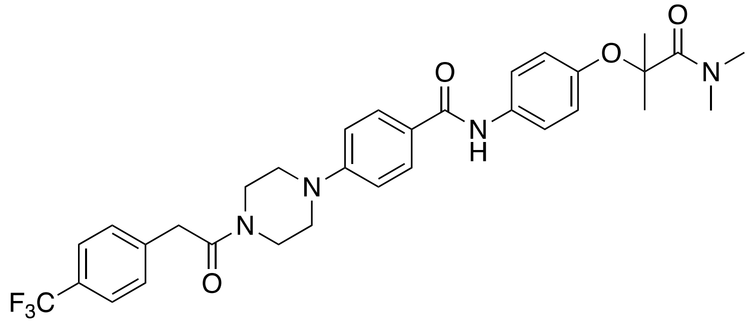

General Procedure A using **68c** (856 mg, 2.08 mmol), HATU (1586 mg, 4.17 mmol), DIPEA (1795 µL, 10.43 mmol), 2-(4-(trifluoromethyl)phenyl)acetic acid (851 mg, 4.17 mmol) gave **68** (434 mg, 0.728 mmol, 35%) as a colourless oil.

^{1}H NMR (400 MHz, DMSO) δ 9.90 (s, 1H), 7.87 (d, *J* = 8.5 Hz, 2H), 7.72 – 7.60 (m, 4H), 7.47 (d, *J* = 7.8 Hz, 2H), 7.03 (d, *J* = 8.6 Hz, 2H), 6.77 – 6.69 (m, 2H), 3.91 (s, 2H), 3.66 (dt, *J* = 18.3, 5.5 Hz, 4H), 3.30 (dt, *J* = 7.2, 3.8 Hz, 4H), 3.14 (s, 3H), 2.86 (s, 3H), 1.51 (s, 6H). **^{13}C NMR** (101 MHz, DMSO) δ 171.82, 168.38, 164.66, 152.57, 150.82, 140.86, 133.57, 130.13, 128.96, 127.26 (q, ^{2}J_CF = 31.8 Hz), 126.94, 125.73, 125.02, 124.98, 124.95, 124.91, 124.13, 123.03, 121.72, 120.24, 117.28, 113.74, 80.40, 79.15, 47.24, 46.86, 44.79, 40.88, 36.89 (2C, $N(CH_3)_2$), 25.54 (2C, $C(CH_3)_2$). **^{19}F NMR** (376 MHz, DMSO) δ -60.78. **HRMS** (ESI-TOF) calc'd for $C_{32}H_{36}N_4O_4F_3$ $[M+H]^+$: 597.2683, found: 597.2690.

tert-Butyl 4-(4-((4-((1-(dimethylamino)-2-methyl-1-oxopropan-2-yl)oxy)phenyl)(methyl)amino)phenyl)piperazine-1-carboxylate – (69b)

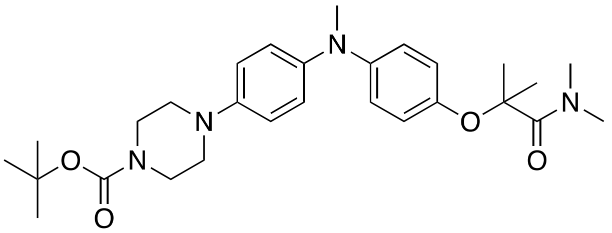

General Procedure B using **63b** (1000 mg, 4.14 mmol), RuPhosPdG3 (350 mg, 0.041 mmol), Cs_2CO_3 (4.05 g, 12.46 mmol) and *tert*-butyl 4-(4-(methylamino)phenyl)piperazine-1-carboxylate (1448 mg, 4.975 mmol) gave **69b** (1314 mg, 2.64 mmol, 64%) as a purple oil.

^{1}H NMR (400 MHz, DMSO) δ 7.54 (s, 2H), 7.04 (s, 2H), 6.85 – 6.72 (m, 4H), 3.85 – 3.39 (m, 6H), 3.28 – 3.09 (m, 8H), 2.86 (s, 3H), 1.52 (s, 6H), 1.47 (s, 3H), 1.43 (s, 6H). **^{13}C NMR** (151 MHz, DMSO) δ 171.82, 153.53, 151.70, 149.18, 143.74, 141.90, 125.28, 121.26, 120.54, 118.67, 118.11, 116.11, 80.56, 79.81, 53.44 (2C, $CON(CH_2)_2$), 46.84 (2C, $N(CH_2)_2$), 42.43, 36.99 (2C, $N(CH_3)_2$), 28.03 (3C, $C(CH_3)_3$), 25.62 (2C, $C(CH_3)_2$). **HRMS** (ESI-TOF) calc'd for $C_{28}H_{41}N_4O_4$ $[M+H]^+$: 497.3128, found: 497.3135.

***N*,*N*,2-Trimethyl-2-(4-(methyl(4-(piperazin-1-yl)phenyl)amino)phenoxy)propenamide – (69c)**

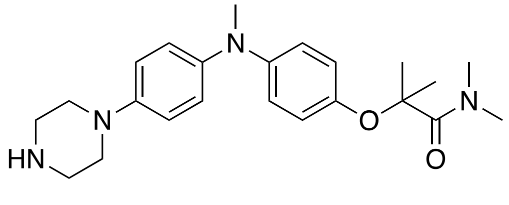

A solution of **69b** (1275 mg, 2.57 mmol) in CH_2Cl_2 (45 mL) was prepared; trifluoroacetic acid (30 mL) was then added. The resultant mixture was stirred for 3 h. The solvent was removed under vacuum. The crude mixture was purified using flash reverse phase chromatography (5% - 95% ACN, in H_2O (0.005 N HCl)) to give **69c** (875 mg, 2.21 mmol, 86%).

^{1}H NMR (400 MHz, DMSO) δ 6.94 – 6.84 (m, 4H), 6.77 – 6.70 (m, 2H), 6.67 – 6.60 (m, 2H), 3.27 (dd, *J* = 6.9, 3.5 Hz, 4H), 3.17 (d, *J* = 7.6 Hz, 7H), 3.10 (s, 3H), 2.84 (s, 3H), 1.45 (s, 6H). **^{13}C NMR** (101 MHz, DMSO) δ 172.15, 148.62, 145.44, 144.19, 142.37, 122.29, 119.52, 118.79, 117.54, 80.56, 46.51 (2C, $N(CH_2)_2$), 42.86, 40.46 (2C, $NH(CH_2)_2$), 37.09, 37.01, 25.65 (2C, $C(CH_3)_2$). **HRMS** (ESI-TOF) calc'd for $C_{23}H_{33}N_4O_2$ $[M+H]^+$: 397.2604, found: 397.2606.

***N,N*,2-Trimethyl-2-(4-(methyl(4-(4-(2-(4-(trifluoromethyl)phenyl)acetyl)piperazin-1-yl)phenyl)amino)phenoxy)propenamide – (IOX7, 69)**

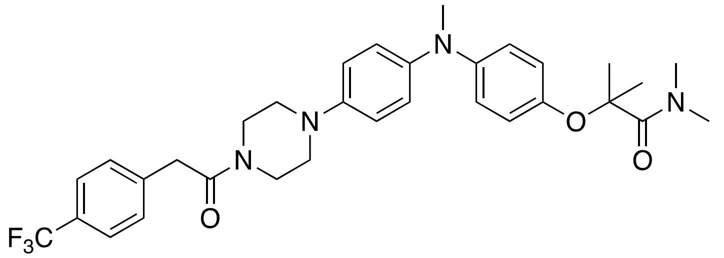

General Procedure A using **69c** (476 mg, 1.20 mmol), DIPEA (1033 μL, 6.01 mmol), HATU (1370 mg, 3.60 mmol), 4-(trifluoromethyl)phenyl acetic acid (367 mg, 1.80 mmol) gave **69** (220 mg, 0.37 mmol, 32%) as a yellow oil.

^{1}H NMR (400 MHz, DMSO) δ 7.73 – 7.63 (m, 2H), 7.51 – 7.44 (m, 2H), 6.90 (s, 4H), 6.78 – 6.71 (m, 2H), 6.68 – 6.62 (m, 2H), 3.89 (s, 2H), 3.63 (dt, *J* = 14.7, 5.1 Hz, 4H), 3.18 (s, 3H), 3.12 (s, 3H), 3.03 (t, *J* = 5.2 Hz, 4H), 2.85 (s, 3H), 1.46 (s, 6H). **^{13}C NMR** (101 MHz, DMSO) δ 172.01, 168.27, 149.71, 148.32, 146.17, 144.26, 141.85, 140.97, 130.14, 124.98, 122.64, 118.98, 118.71, 117.30, 80.48, 54.91, 49.42, 49.01, 45.24, 41.24, 36.92 (2C, $N(CH_3)_2$), 25.60 (2C, $C(CH_3)_2$). **^{19}F NMR** (376 MHz, DMSO) δ -60.78. **HRMS** (ESI-TOF) calc'd for $C_{32}H_{38}N_4O_3F_3$ $[M+H]^+$: 583.2896, found: 583.2899.

NMR Spectra of Key Compounds

12 – ^{1}H

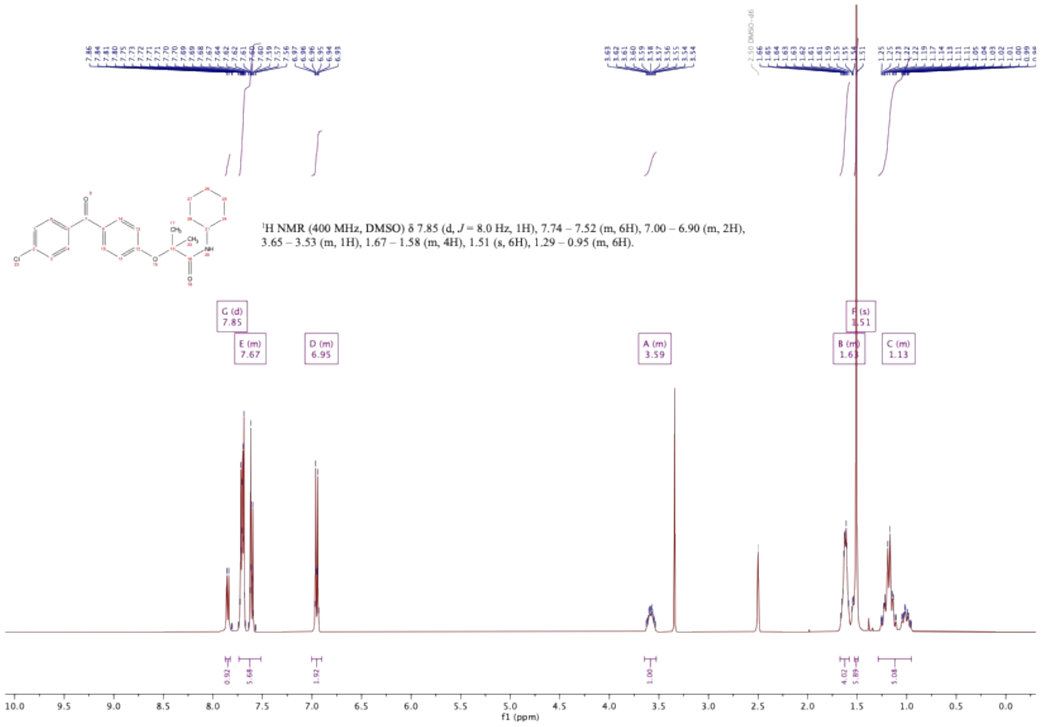

12 – ^{13}C

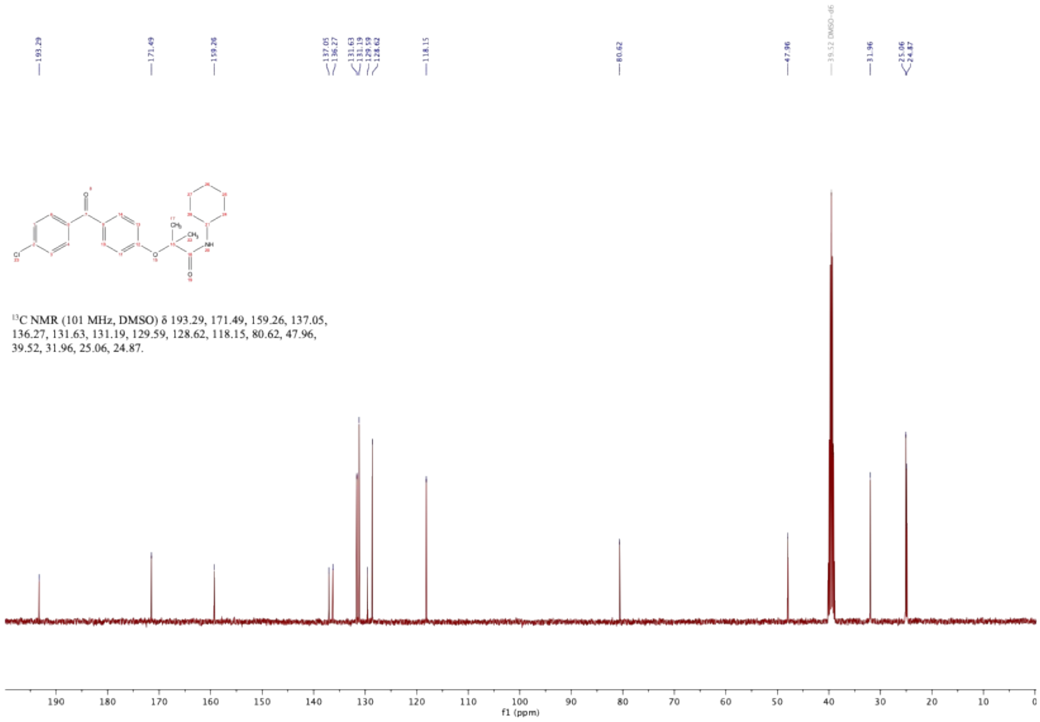
13C NMR (101 MHz, DMSO) δ 193.29, 171.49, 159.26, 137.05, 136.27, 131.63, 131.19, 129.59, 128.62, 118.15, 80.62, 47.96, 39.52, 31.96, 25.06, 24.87.
f1 (ppm)

28 – ¹H

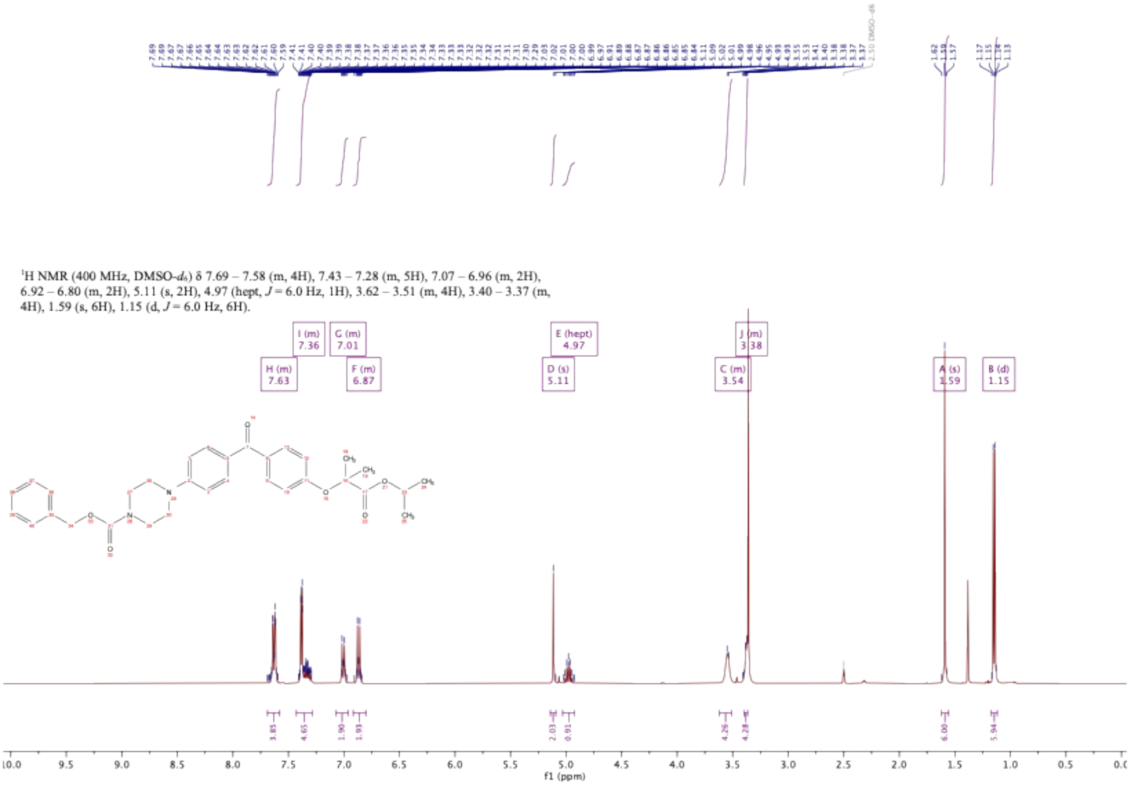
1H NMR (400 MHz, DMSO-d6) δ 7.69 – 7.58 (m, 4H), 7.43 – 7.28 (m, 5H), 7.07 – 6.96 (m, 2H), 6.92 – 6.80 (m, 2H), 5.11 (s, 2H), 4.97 (hept, J = 6.0 Hz, 1H), 3.62 – 3.51 (m, 4H), 3.40 – 3.37 (m, 4H), 1.59 (s, 6H), 1.15 (d, J = 6.0 Hz, 6H).
I (m) 7.36
G (m) 7.01
H (m) 7.63
F (m) 6.87
E (hept) 4.97
D (s) 5.11
J (m) 3.38
C (m) 3.54
A (s) 1.59
B (d) 1.15
f1 (ppm)

28 – 13C

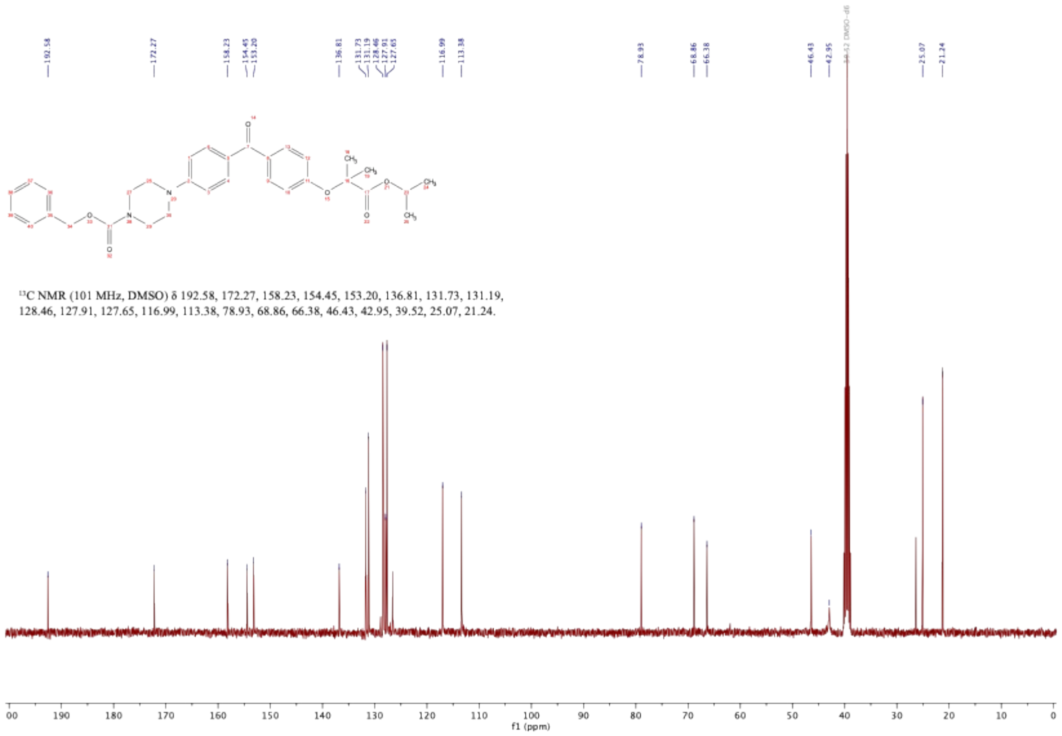
13C NMR (101 MHz, DMSO) δ 192.58, 172.27, 158.23, 154.45, 153.20, 136.81, 131.73, 131.19, 128.46, 127.91, 127.65, 116.99, 113.38, 78.93, 68.86, 66.38, 46.43, 42.95, 39.52, 25.07, 21.24.
f1 (ppm)

35 – ¹H

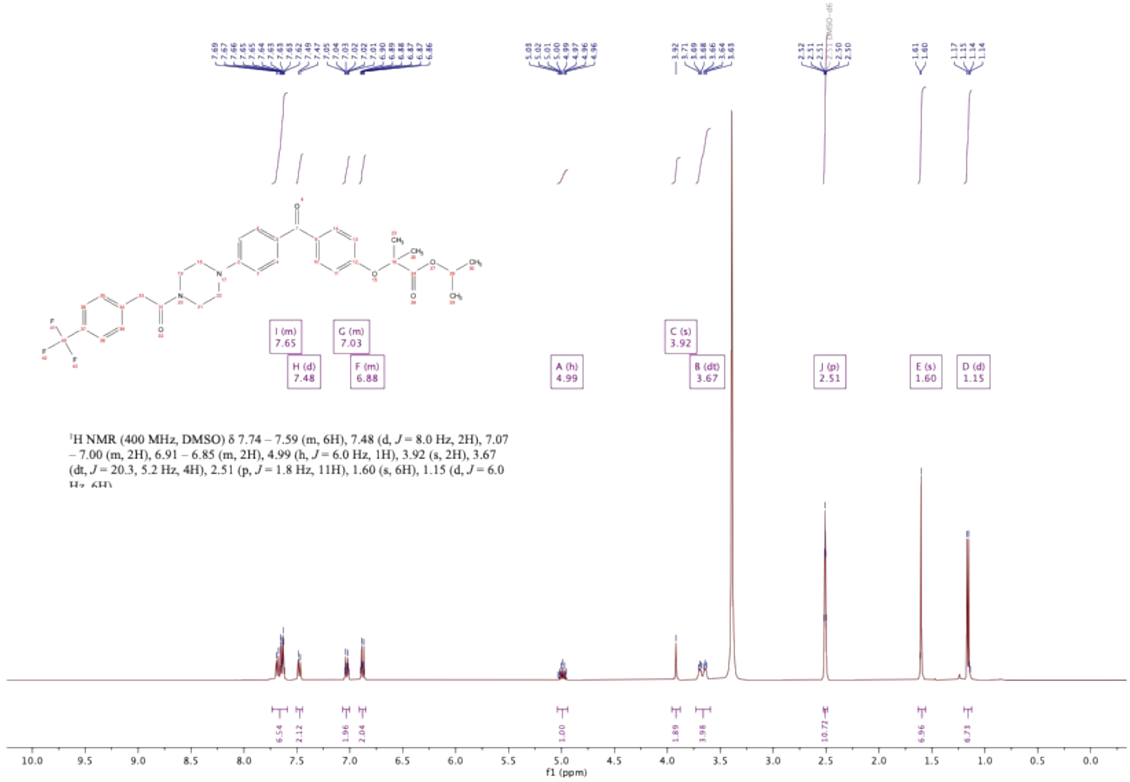
I (m) 7.65
H (d) 7.48
G (m) 7.03
F (m) 6.88
A (h) 4.99
C (s) 3.92
B (dt) 3.67
J (p) 2.51
E (s) 1.60
D (d) 1.15
1H NMR (400 MHz, DMSO) δ 7.74 – 7.59 (m, 6H), 7.48 (d, J = 8.0 Hz, 2H), 7.07 – 7.00 (m, 2H), 6.91 – 6.85 (m, 2H), 4.99 (h, J = 6.0 Hz, 1H), 3.92 (s, 2H), 3.67 (dt, J = 20.3, 5.2 Hz, 4H), 2.51 (p, J = 1.8 Hz, 11H), 1.60 (s, 6H), 1.15 (d, J = 6.0
f1 (ppm)

35 – ¹³C

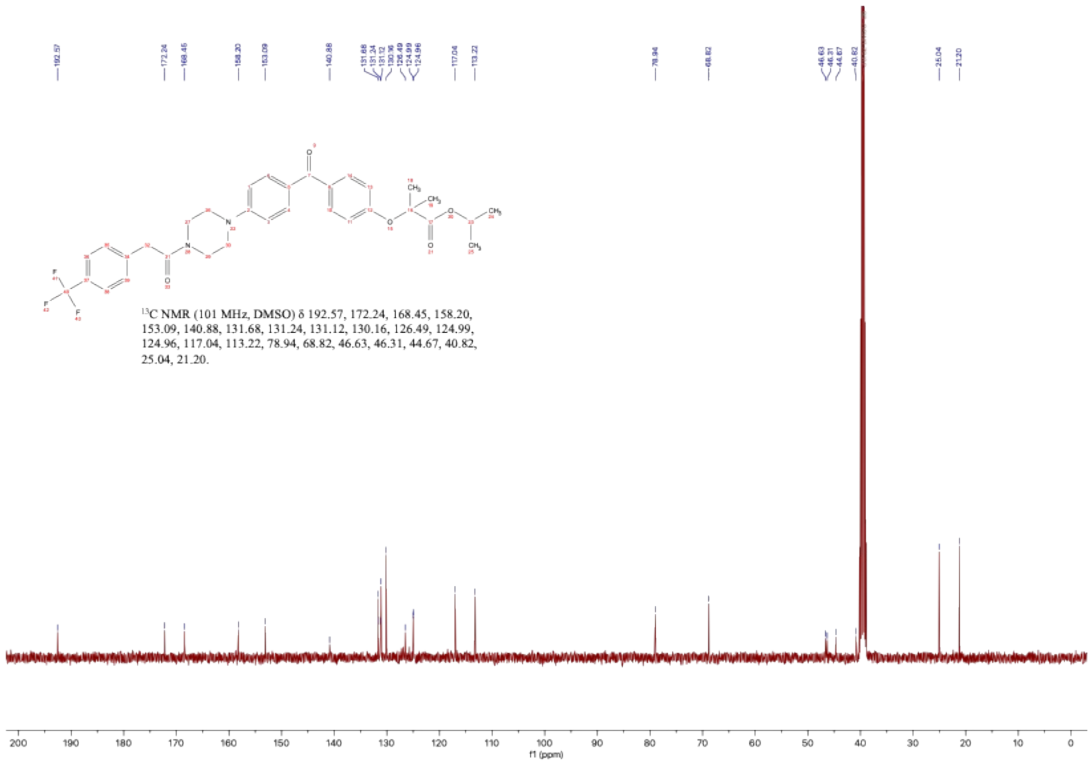
13C NMR (101 MHz, DMSO) δ 192.57, 172.24, 168.45, 158.20, 153.09, 140.88, 131.68, 131.24, 131.12, 130.16, 126.49, 124.99, 124.96, 117.04, 113.22, 78.94, 68.82, 46.63, 46.31, 44.67, 40.82, 25.04, 21.20.
f1 (ppm)

35 – ^{19}F

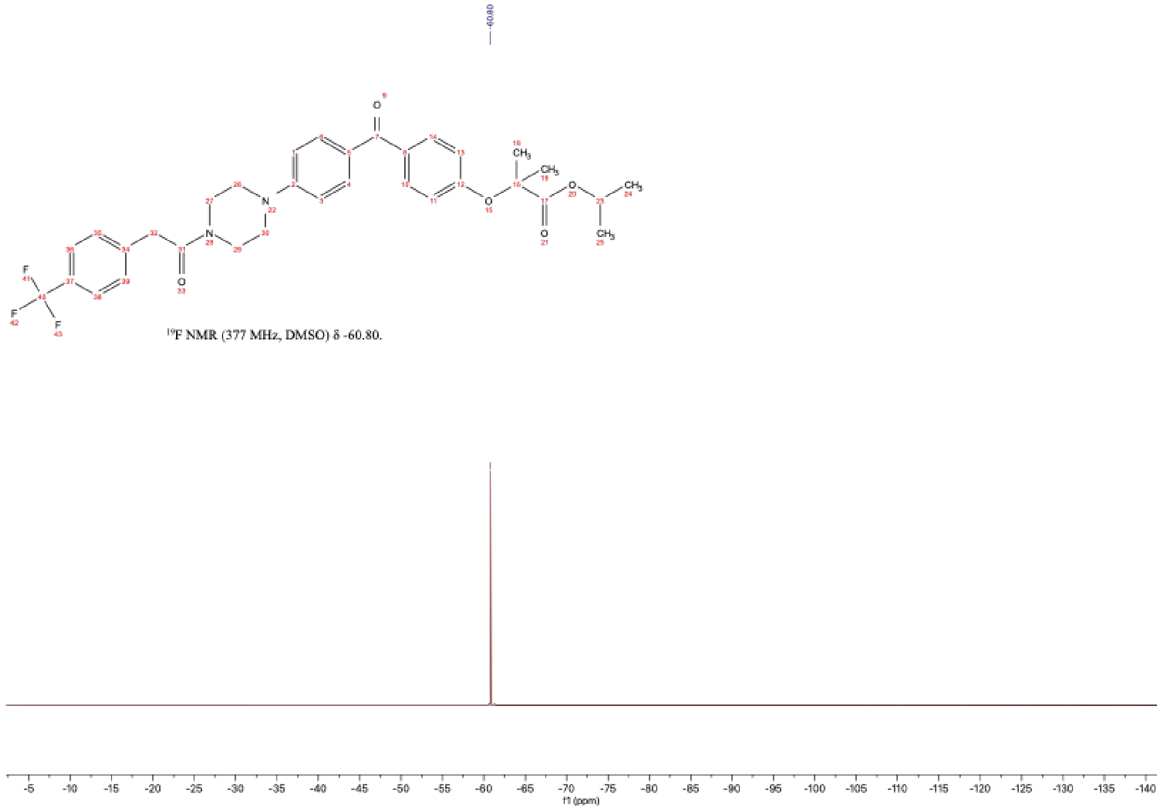
-60.80
19F NMR (377 MHz, DMSO) δ -60.80.
f1 (ppm)

41 – ^{1}H

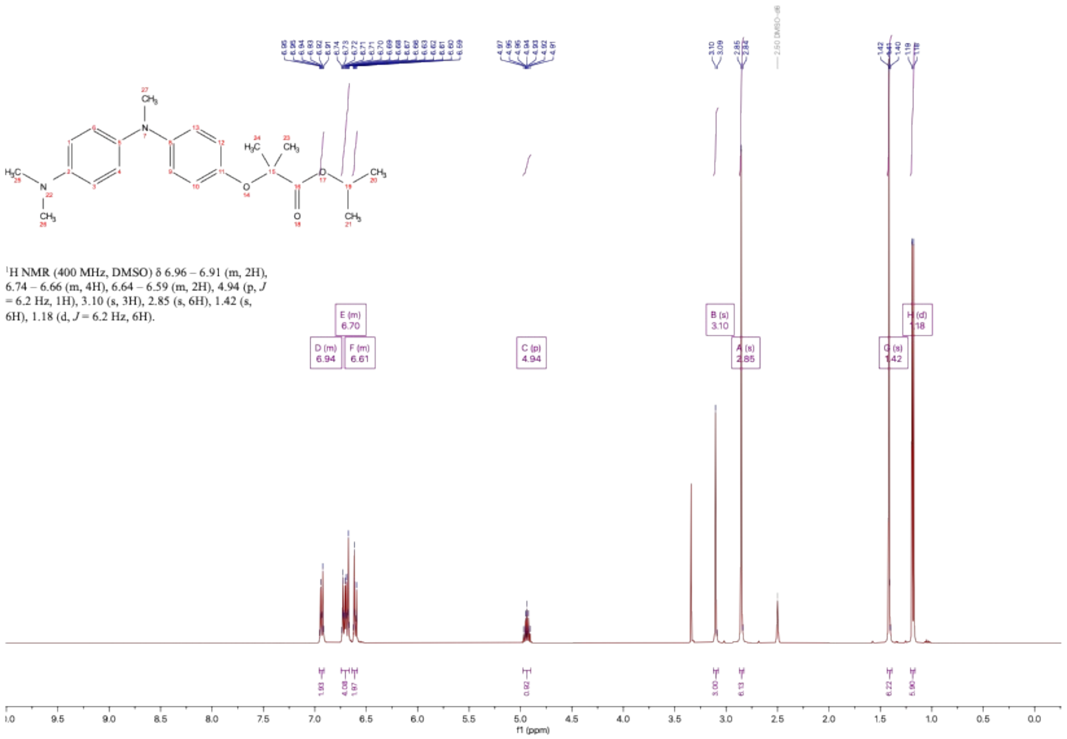
¹H NMR (400 MHz, DMSO) δ 6.96 – 6.91 (m, 2H), 6.74 – 6.66 (m, 4H), 6.64 – 6.59 (m, 2H), 4.94 (p, J = 6.2 Hz, 1H), 3.10 (s, 3H), 2.85 (s, 6H), 1.42 (s, 6H), 1.18 (d, J = 6.2 Hz, 6H).
f1 (ppm)

41 – ^{13}C

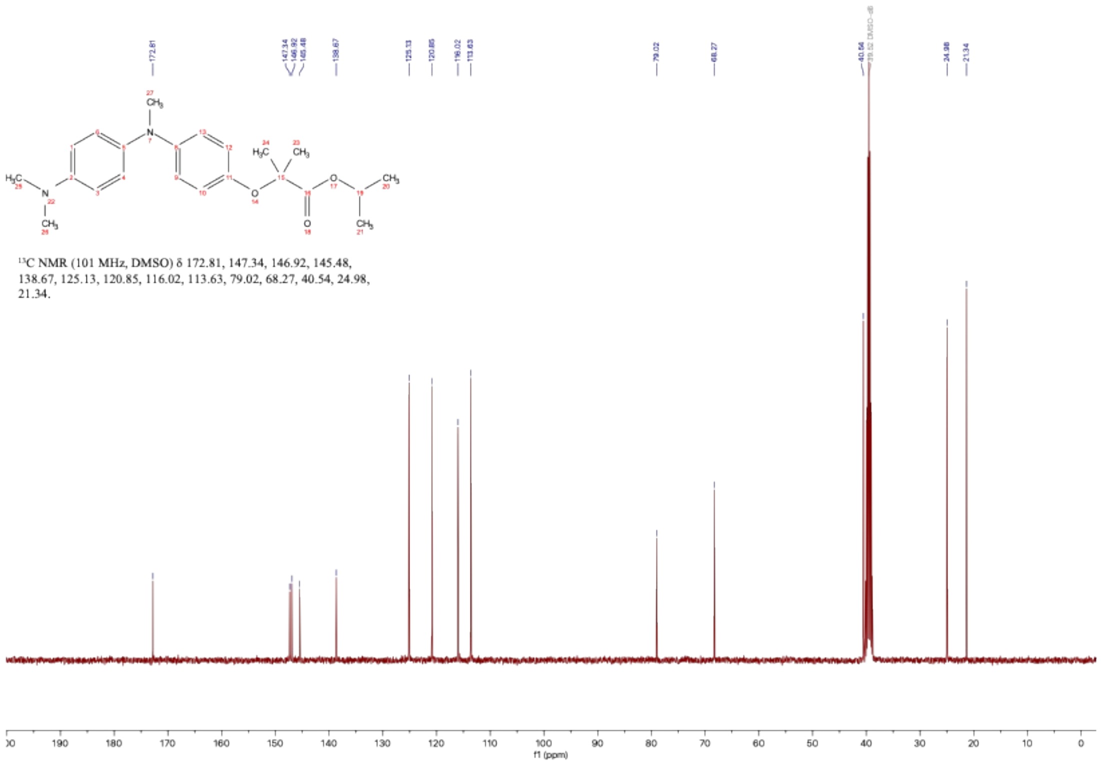
¹³C NMR (101 MHz, DMSO) δ 172.81, 147.34, 146.92, 145.48, 138.67, 125.13, 120.85, 116.02, 113.63, 79.02, 68.27, 40.54, 24.98, 21.34.
f1 (ppm)

42 – ^{1}H

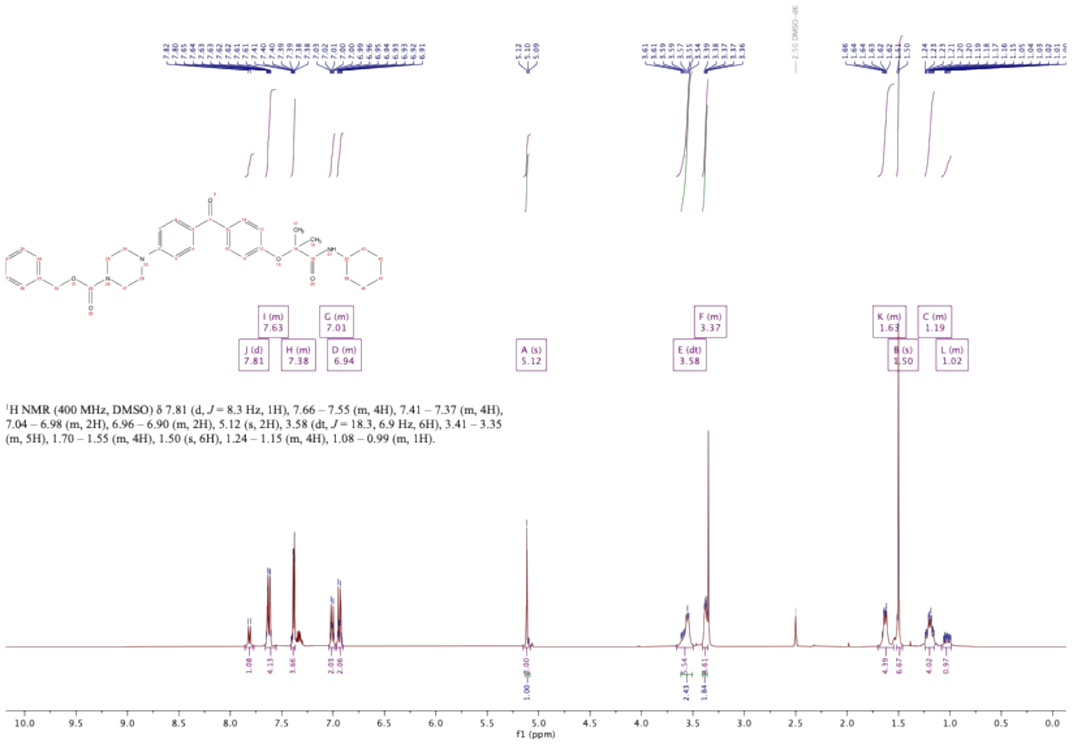

^{1}H NMR (400 MHz, DMSO) δ 7.81 (d, J = 8.3 Hz, 1H), 7.66 – 7.55 (m, 4H), 7.41 – 7.37 (m, 4H), 7.04 – 6.98 (m, 2H), 6.96 – 6.90 (m, 2H), 5.12 (s, 2H), 3.58 (dt, J = 18.3, 6.9 Hz, 6H), 3.41 – 3.35 (m, 5H), 1.70 – 1.55 (m, 4H), 1.50 (s, 6H), 1.24 – 1.15 (m, 4H), 1.08 – 0.99 (m, 1H).
f1 (ppm)

42 - ^{13}C

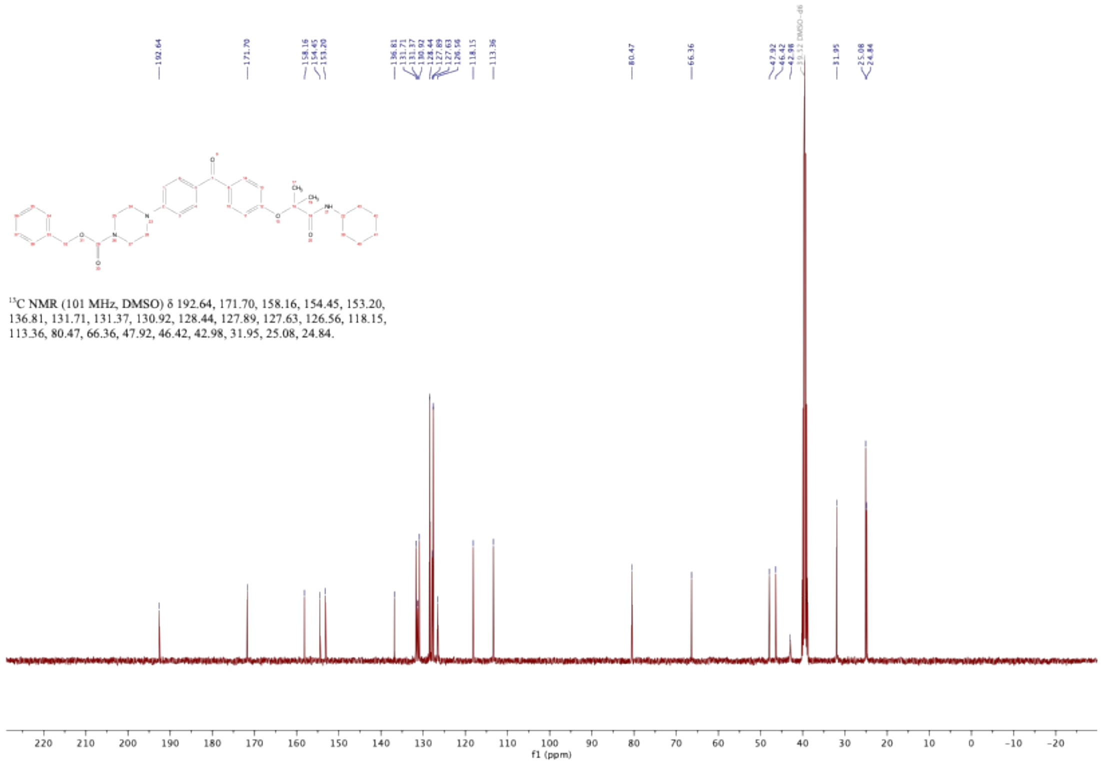

^{13}C NMR (101 MHz, DMSO) δ 192.64, 171.70, 158.16, 154.45, 153.20, 136.81, 131.71, 131.37, 130.92, 128.44, 127.89, 127.63, 126.56, 118.15, 113.36, 80.47, 66.36, 47.92, 46.42, 42.98, 31.95, 25.08, 24.84.
f1 (ppm)

66 – ^{1}H

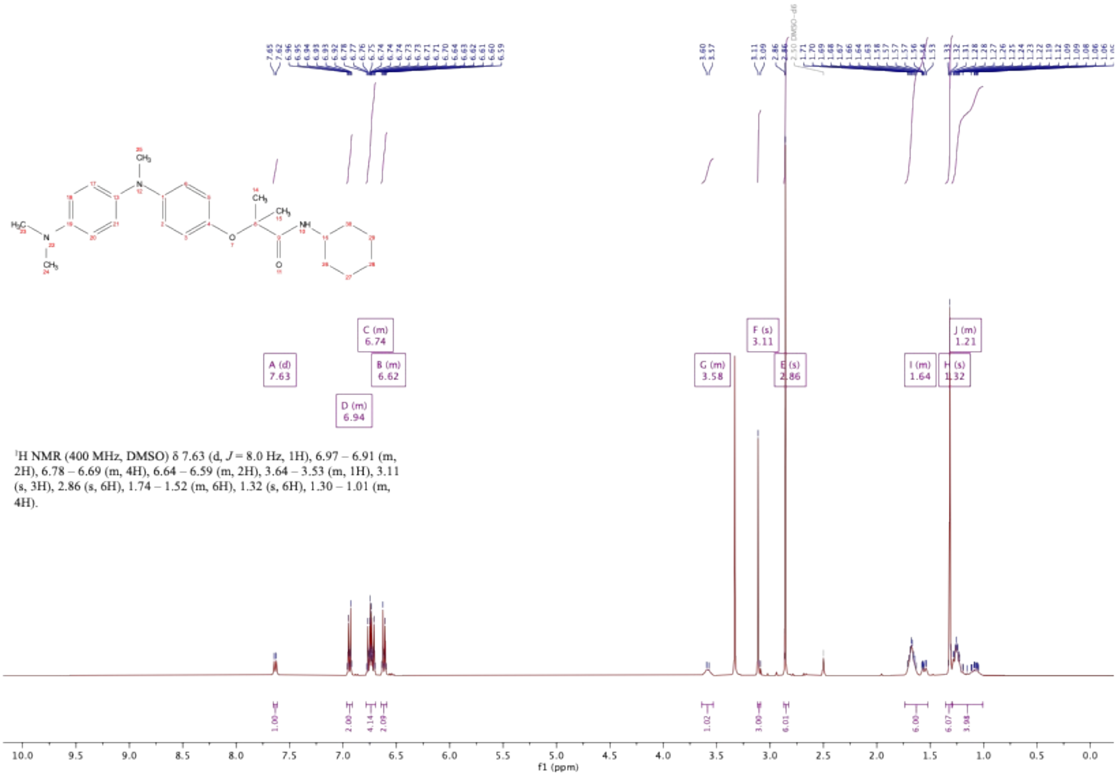
^{1}H NMR (400 MHz, DMSO) δ 7.63 (d, J = 8.0 Hz, 1H), 6.97 – 6.91 (m, 2H), 6.78 – 6.69 (m, 4H), 6.64 – 6.59 (m, 2H), 3.64 – 3.53 (m, 1H), 3.11 (s, 3H), 2.86 (s, 6H), 1.74 – 1.52 (m, 6H), 1.32 (s, 6H), 1.30 – 1.01 (m, 4H).
f1 (ppm)

66 – ^{13}C

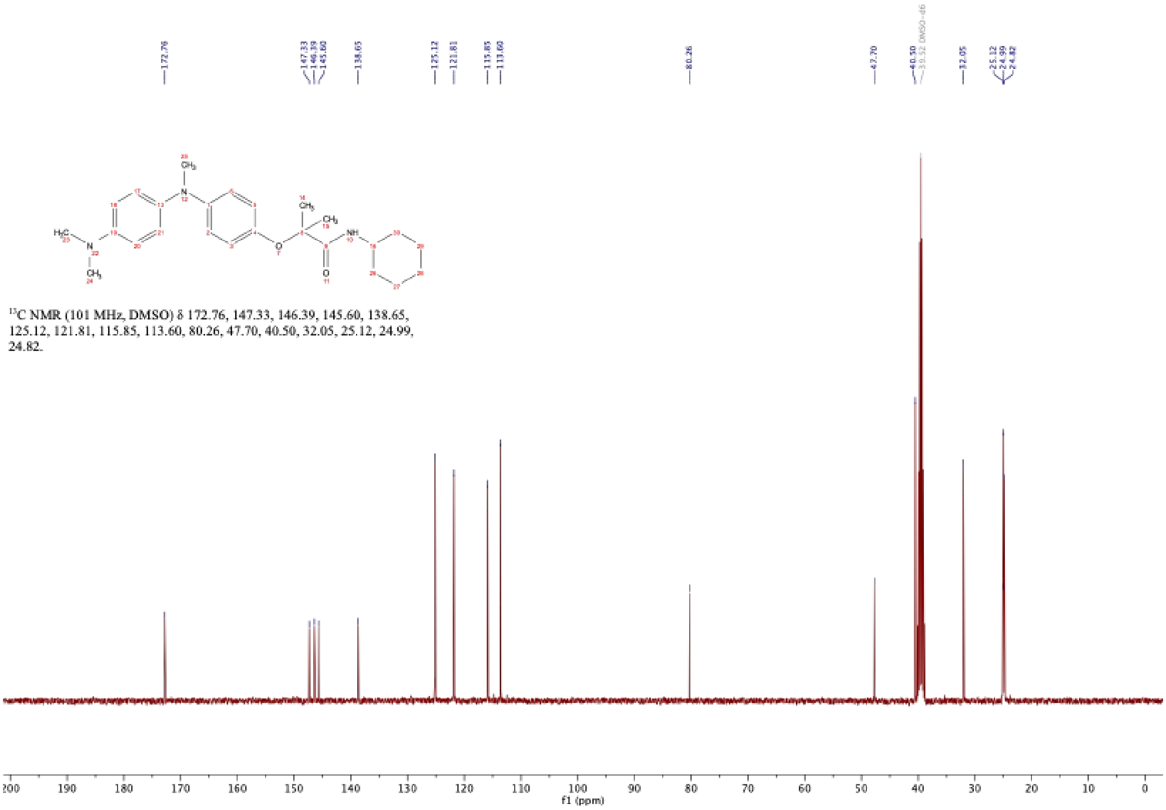
^{13}C NMR (101 MHz, DMSO) δ 172.76, 147.33, 146.39, 145.60, 138.65, 125.12, 121.81, 115.85, 113.60, 80.26, 47.70, 40.50, 32.05, 25.12, 24.99, 24.82.
f1 (ppm)

68 – ^{1}H

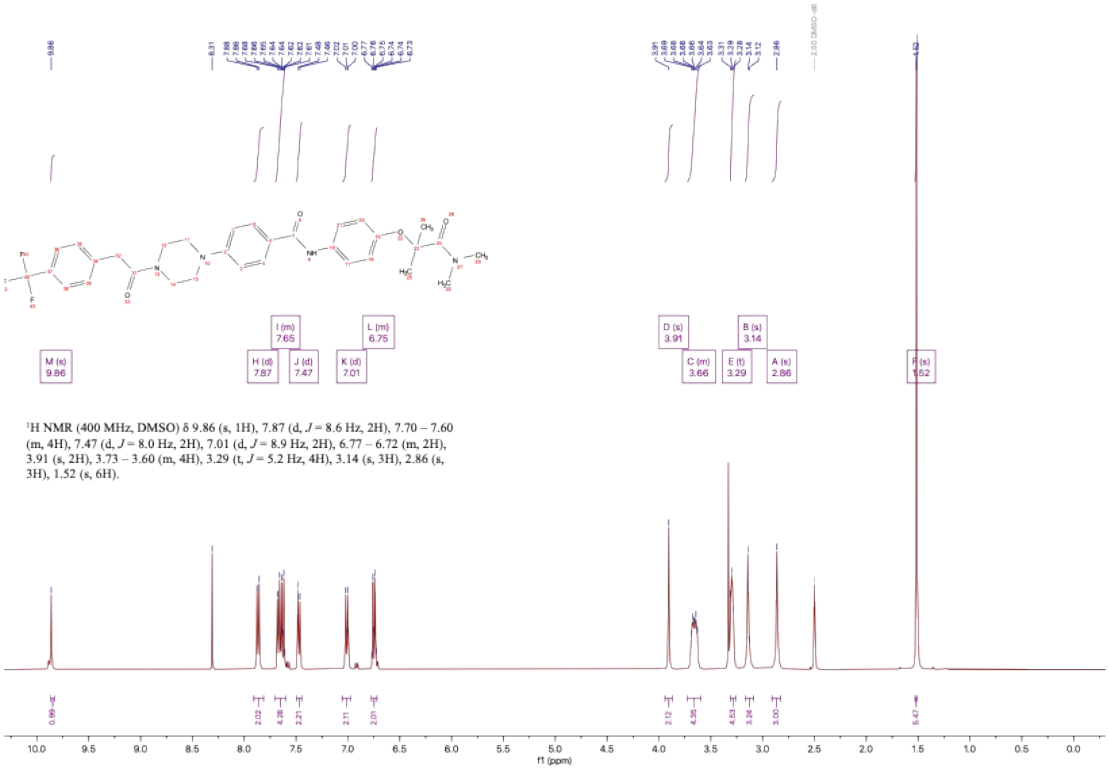

1H NMR (400 MHz, DMSO) δ 9.86 (s, 1H), 7.87 (d, J = 8.6 Hz, 2H), 7.70 – 7.60 (m, 4H), 7.47 (d, J = 8.0 Hz, 2H), 7.01 (d, J = 8.9 Hz, 2H), 6.77 – 6.72 (m, 2H), 3.91 (s, 2H), 3.73 – 3.60 (m, 4H), 3.29 (t, J = 5.2 Hz, 4H), 3.14 (s, 3H), 2.86 (s, 3H), 1.52 (s, 6H).
f1 (ppm)

68 – ^{13}C

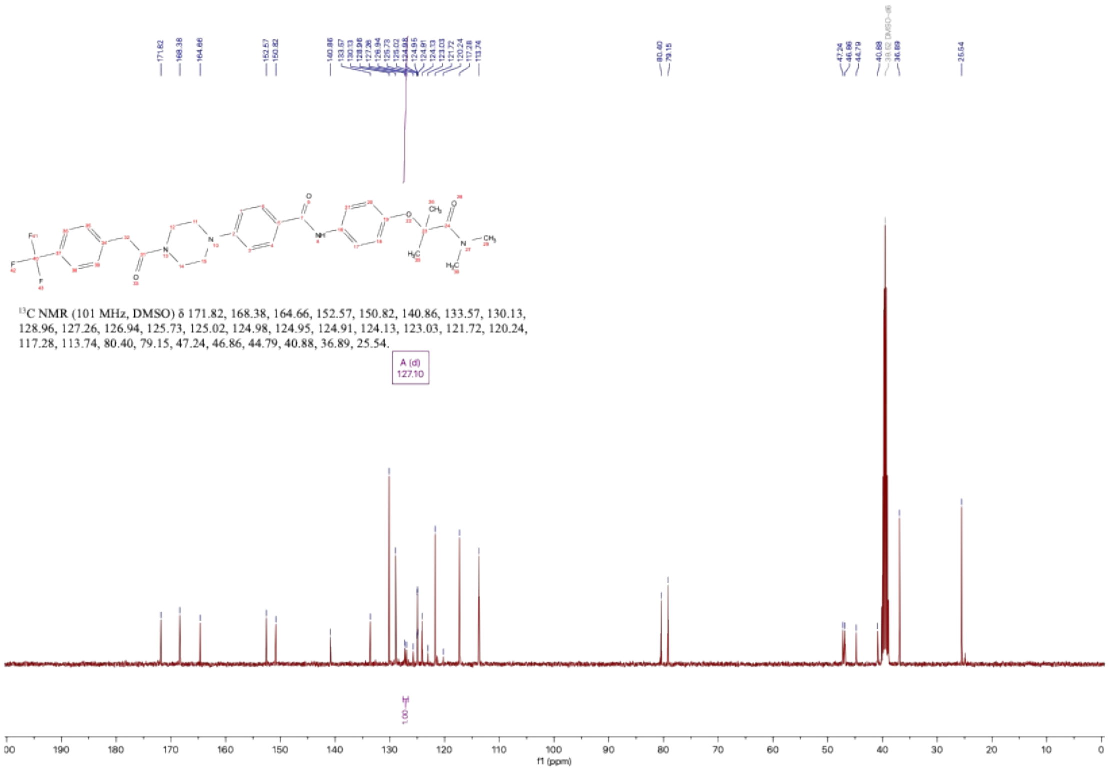

13C NMR (101 MHz, DMSO) δ 171.82, 168.38, 164.66, 152.57, 150.82, 140.86, 133.57, 130.13, 128.96, 127.26, 126.94, 125.73, 125.02, 124.98, 124.95, 124.91, 124.13, 123.03, 121.72, 120.24, 117.28, 113.74, 80.40, 79.15, 47.24, 46.86, 44.79, 40.88, 36.89, 25.54.
f1 (ppm)

68 – ^{19}F

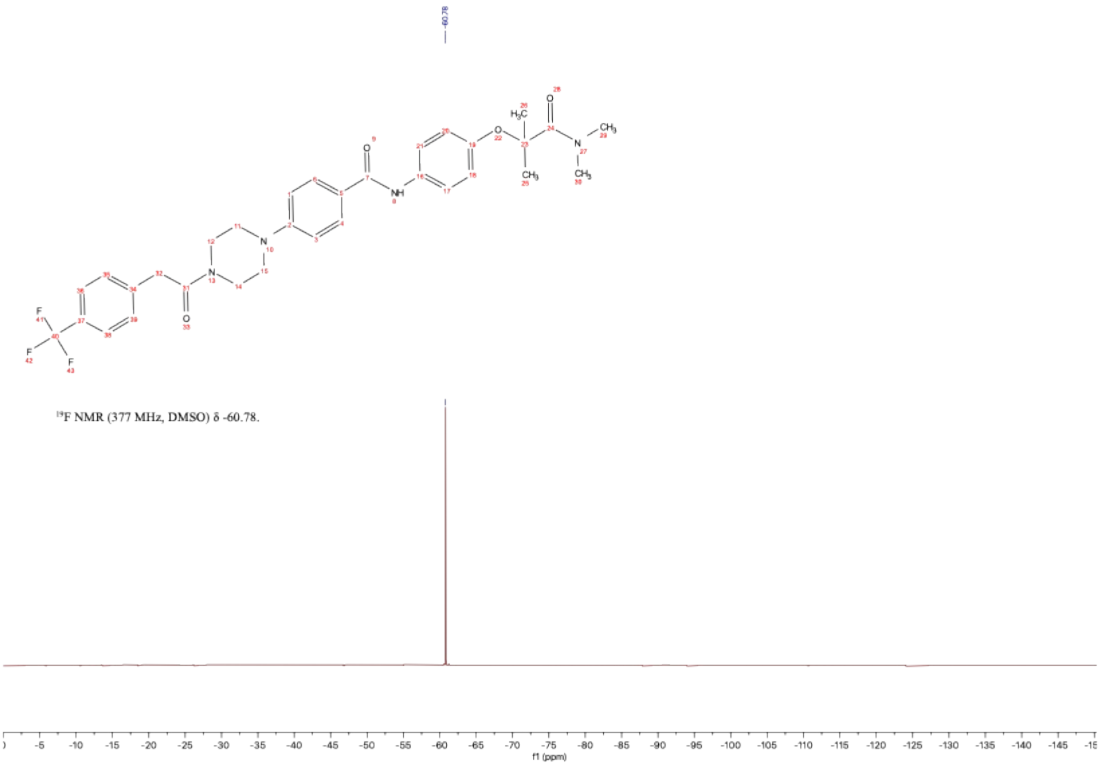

69 (IOX7) – ^{1}H

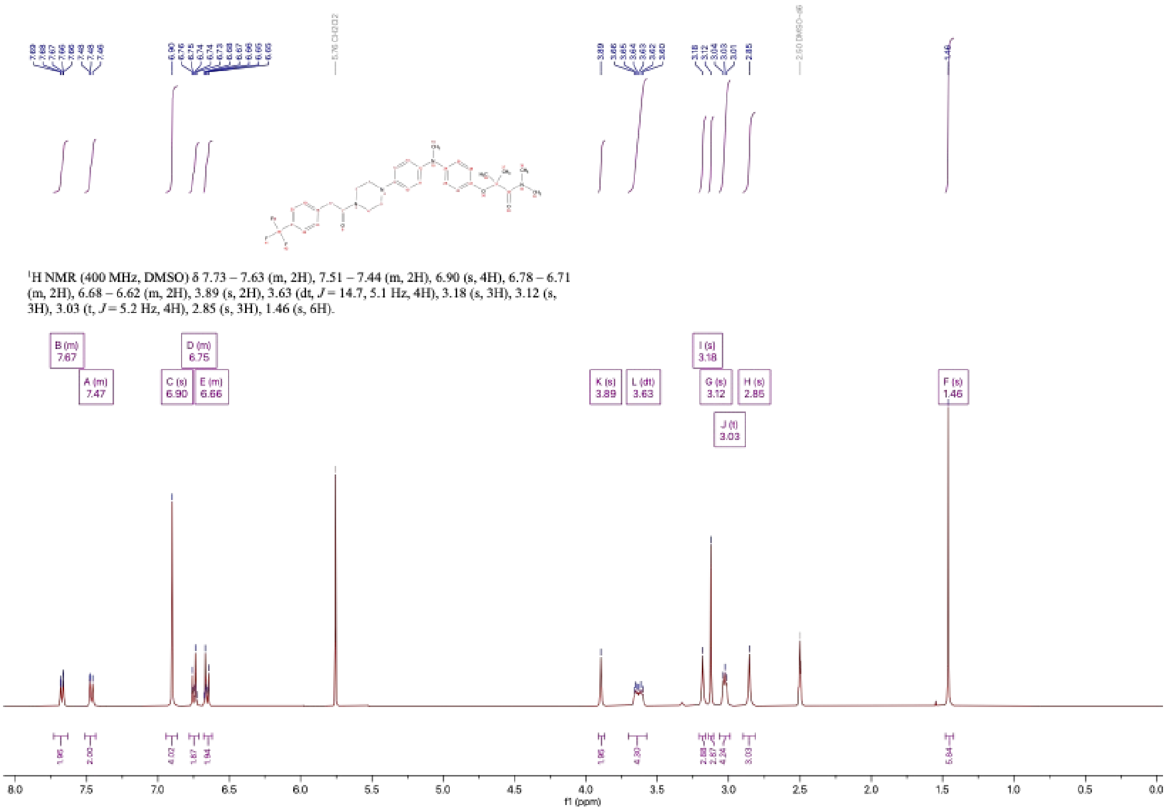

69 (IOX7) – ^{13}C

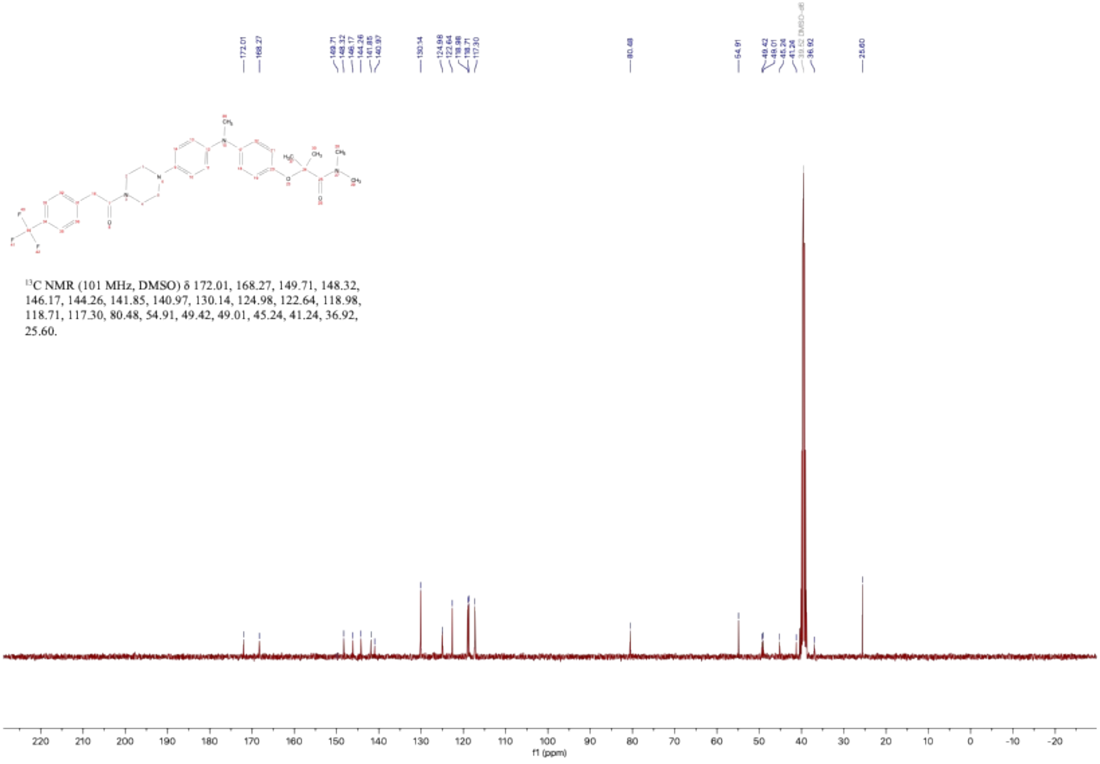
172.01
168.27
149.71
148.32
146.17
144.26
141.85
140.97
130.14
124.98
122.64
118.98
118.71
117.30
80.48
54.91
49.42
49.01
45.24
41.24
39.52 DMSO-d6
36.92
25.60
^{13}C NMR (101 MHz, DMSO) δ 172.01, 168.27, 149.71, 148.32, 146.17, 144.26, 141.85, 140.97, 130.14, 124.98, 122.64, 118.98, 118.71, 117.30, 80.48, 54.91, 49.42, 49.01, 45.24, 41.24, 36.92, 25.60.
f1 (ppm)

69 (IOX7) – ^{19}F

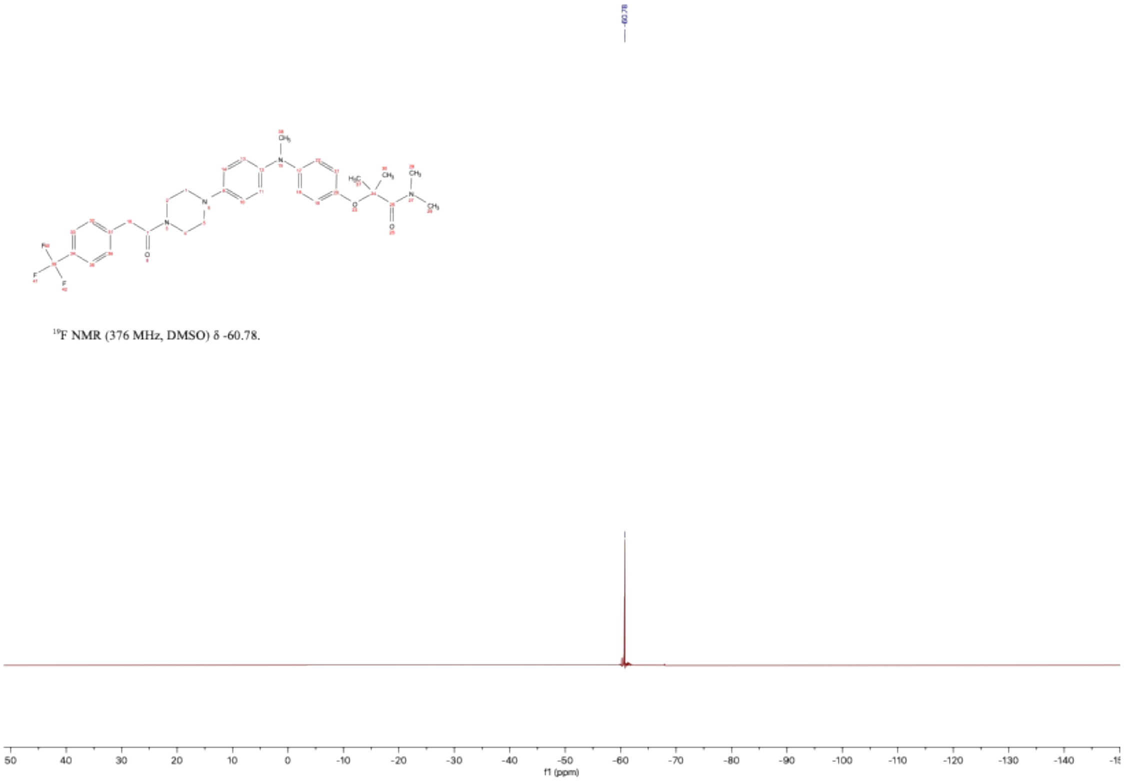
-60.78
^{19}F NMR (376 MHz, DMSO) δ -60.78.
f1 (ppm)

UPLC Traces of Key Compounds

Samples were analysed using an ACQUITY H-Class PLUS UPLC instrument (Waters) equipped with a pre-equilibrated ACQUITY BEH C18 column (2.1 × 50 mm, 1.7 μM pore size, Waters) using a gradient of 1–99%v/v acetonitrile with 0.1%v/v formic acid in water with 0.1%v/v formic acid over 4.0 minutes at a flow rate of 0.5 mL min−1. Instrument control and data processing were performed using MassLynx V4.1 software. The resultant file was then processed in MestreNova.

12

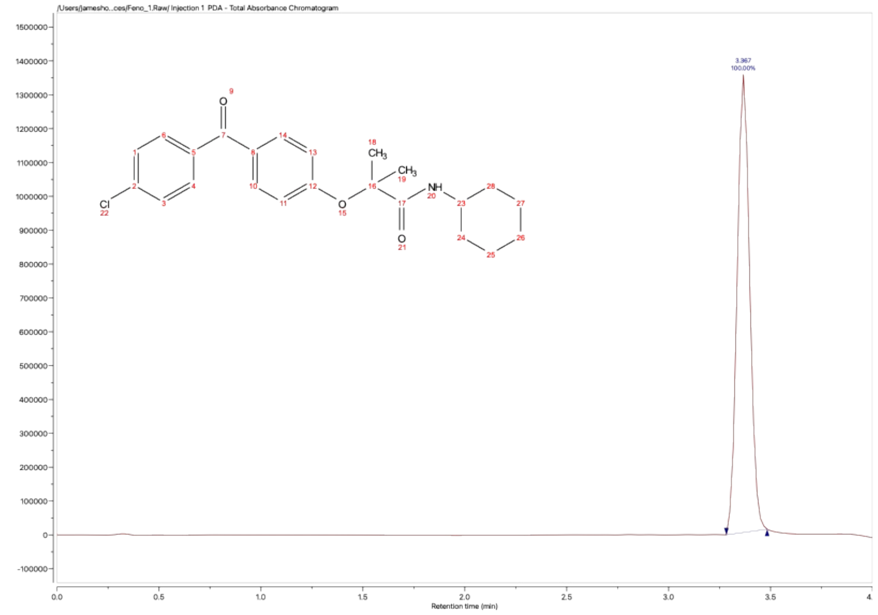
/Users/jamesho...ces/Feno_1.Raw/ Injection 1 PDA - Total Absorbance Chromatogram
3.367
100.00%
Retention time (min)

35

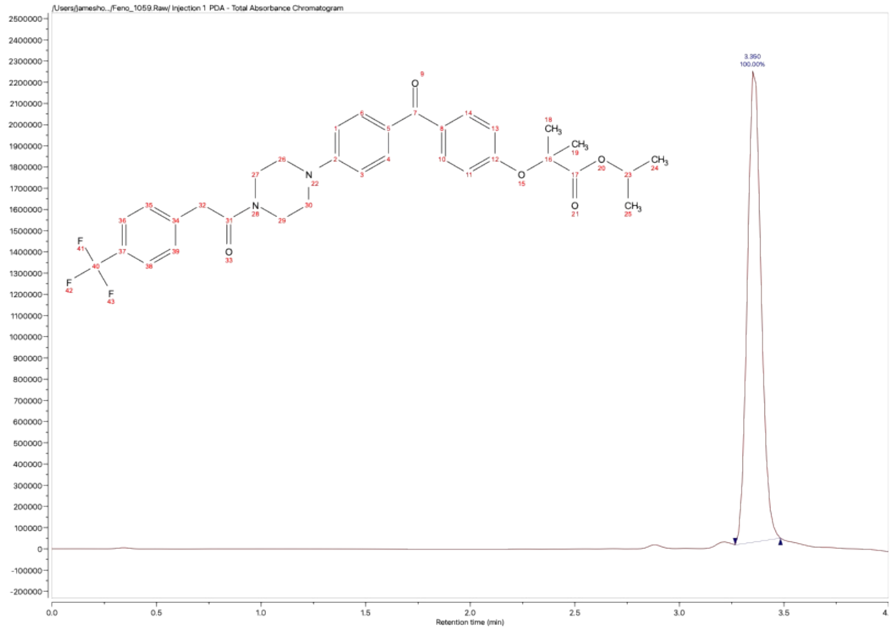
/Users/jamesho.../Feno_1059.Raw/ Injection 1 PDA - Total Absorbance Chromatogram
3.350
100.00%
Retention time (min)

66

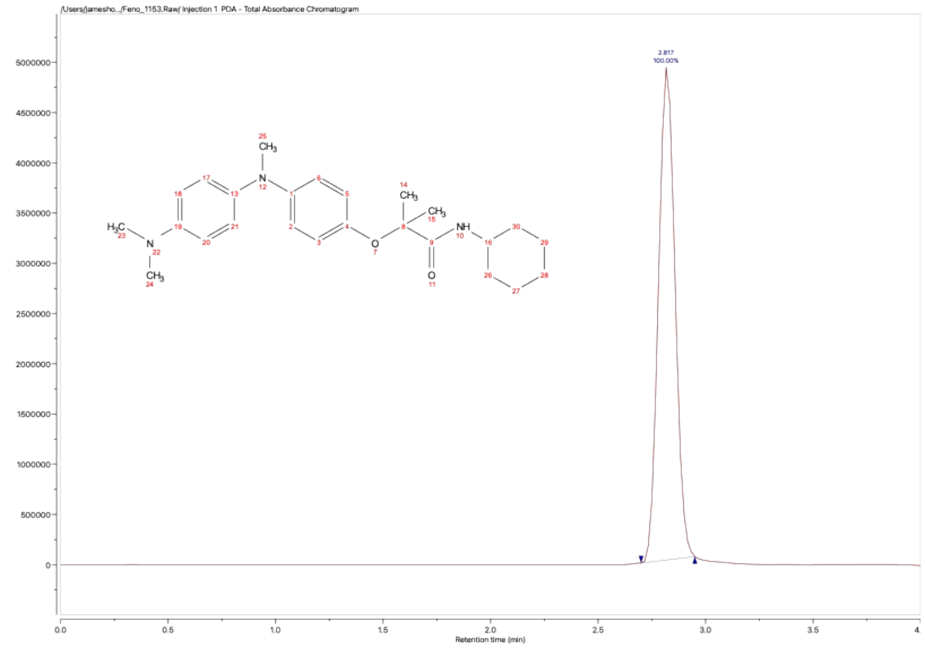
/Users/jamesho.../Feno_1153.Raw/ Injection 1 PDA - Total Absorbance Chromatogram
2.817
100.00%
Retention time (min)

68

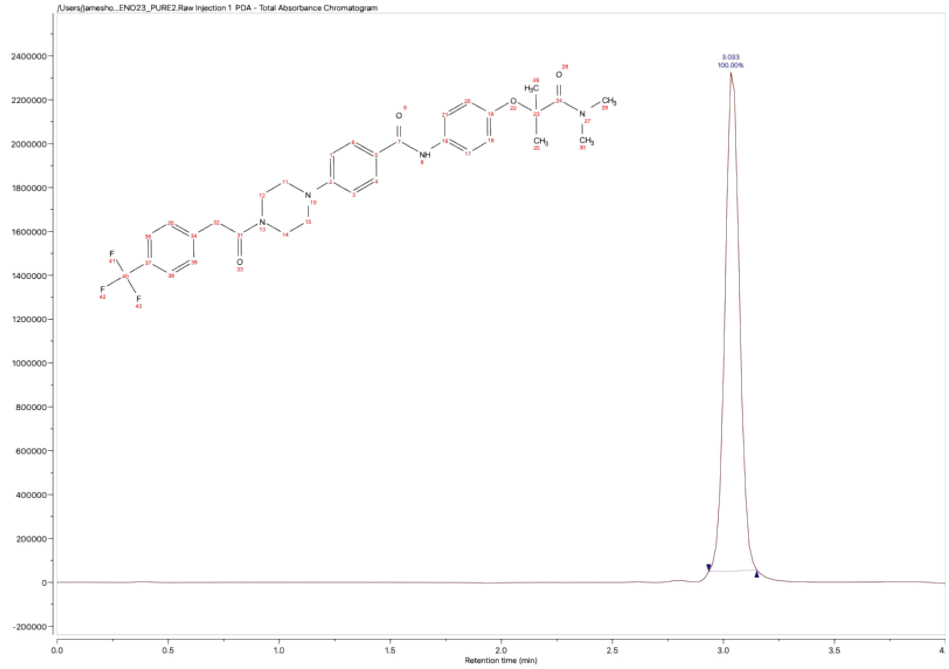
/Users/jamesho...ENO23_PURE2.Raw Injection 1 PDA - Total Absorbance Chromatogram
3.033
100.00%
Retention time (min)

69 (IOX7)

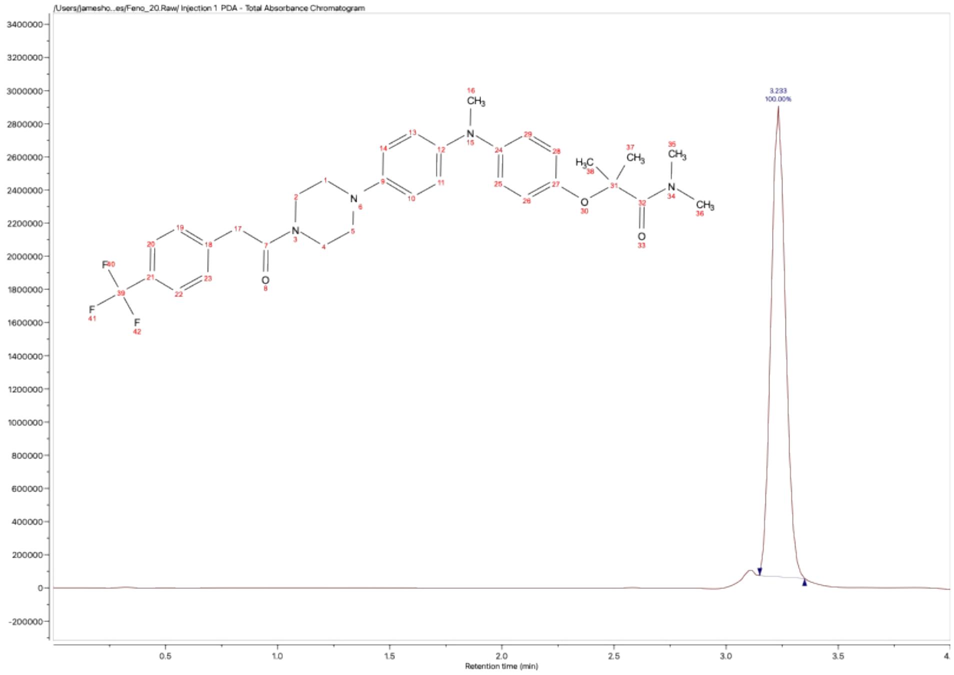
/Users/jamesho...es/Feno_20.Raw/ Injection 1 PDA - Total Absorbance Chromatogram
3.233
100.00%
Retention time (min)

References

1. Divakaruni AS, Rogers GW, Murphy AN. Measuring Mitochondrial Function in Permeabilized Cells Using the Seahorse XF Analyzer or a Clark-Type Oxygen Electrode. Current Protocols in Toxicology. 2014/05/01;60(1).
